# Thermal Processing Shapes Phenolic Profiles in Iodine-Biofortified Kale: Cultivar- and Iodine-Treatment-Associated Patterns Revealed by LC–HRMS

**DOI:** 10.3390/molecules31203660

**Published:** 2026-10-13

**Authors:** Barbara Kusznierewicz, Justyna Waśniowska, Aneta Koronowicz

**Affiliations:** 1 Department of Chemistry, Technology and Biotechnology of Food, Faculty of Chemistry, Gdańsk University of Technology, 11/12 G. Narutowicza St., 80-233 Gdansk, Poland; 2 Department of Human Nutrition and Dietetics, Faculty of Food Technology, University of Agriculture in Krakow, Al. Mickiewicza 21, 31-120 Krakow, Poland; aneta.koronowicz@urk.edu.pl

**Keywords:** curly kale, biofortification, iodine, 5,7-diiodo-8-quinolinol, thermal processing, phenolic compounds

## Abstract

The impact of culinary processing on the phenolic profile of iodine-biofortified kale remains insufficiently resolved at the compound level. Green- and red-leaved kale grown without iodine supplementation or biofortified with KIO_3_ or 5,7-diiodo-8-quinolinol (DIQ) was subjected to blanching, boiling, or steaming. LC–HRMS profiling resulted in 135 phenolic annotations, including three compounds confirmed using authentic standards, while selected major phenolics were additionally quantified or semi-quantified by PDA detection. Multivariate analysis revealed pronounced cultivar-related differences. Among processed samples, thermal processing accounted for the largest proportion of phenolic variation (61.6% in green and 68.5% in red kale), while iodine treatment accounted for 9.3% and 7.2%, and the Processing × Iodine component for 24.2% and 19.6%, respectively. Compound-level analysis revealed pronounced differences between steamed and boiled samples, with predominantly higher relative LC–HRMS signals after steaming, especially in red-leaved kale. Processing-related phenolic patterns also differed among Control, KIO_3_, and DIQ sample pools. Because each cultivar × iodine treatment combination represented a single pooled biological source, iodine-associated differences were interpreted descriptively rather than as inferential treatment effects. Overall, thermal processing was a major determinant of phenolic composition, while processing-related patterns varied among iodine-treatment pools.

## 1. Introduction

Kale is commonly consumed after culinary processing, which can substantially alter its phytochemical profile. The net effect depends on the method and intensity of the process and reflects the balance between several mechanisms, including increased extractability due to loosening of the plant matrix and potential losses due to thermal-oxidative degradation and (in the case of water-based treatments) leaching into the processing medium. Review studies indicate that processes such as boiling, blanching or steaming can alter the polyphenol content in fruit and vegetables in various ways, and a comparison of methods must take into account both degradation and changes in bioavailability/release from the matrix [1,2,3].

Blanching, boiling, and steaming were selected as representative moist-heat treatments relevant to leafy *Brassica* vegetables. These methods differ in the degree of direct contact with water and therefore allow processing effects associated with heating and matrix softening to be considered together with the potential contribution of aqueous leaching. Dry-heat methods, such as baking and stir-frying, involve additional factors, including dehydration, different surface-temperature conditions, and, in the case of stir-frying, oil uptake; they were therefore outside the predefined scope of the present study.

At the same time, there is growing interest in iodine biofortification as a food strategy to support the supply of this element. Iodine was selected in the present study because it is an essential micronutrient required for thyroid hormone synthesis, whereas terrestrial plant foods generally contain relatively low iodine concentrations. Agronomic biofortification of leafy vegetables has therefore been investigated as a complementary approach to improving dietary iodine supply [4,5]. In the case of kale, both the classic approach using potassium iodate (KIO_3_) and newer solutions based on iodine quinoline derivatives have been described, which are effective in enrichment under hydroponic cultivation conditions and may affect the chemical composition parameters of the raw material [6]. At the same time, the literature on plant physiology indicates that iodine application may be associated with changes in oxidative–antioxidative metabolism and in the accumulation of phenolic compounds, and the response depends on the form of iodine and stress conditions [7]. From the point of view of the consumer and food technology, this means that the assessment of the ‘biofortification effect’ should take into account not only the raw material, but also real culinary processing scenarios that may alter the final profile of bioactive compounds. In this context, reports on iodine-enriched kale are relevant, as they demonstrate the impact of heat treatment parameters on iodine content and selected quality/antioxidant activity indicators depending on the cultivar and enrichment method [8].

An additional dimension to the problem is genotypic diversity: transcriptomic analyses and metabolite profiling have revealed different metabolic backgrounds between green and red kale forms, including differences related to the phenylpropanoid pathway and pigmentation (e.g., anthocyanins), which may translate into different sensitivity of the phenolic profile to environmental and technological stimuli [9]. The present study extends our previous work demonstrating effective iodine enrichment of kale using KIO_3_ and 5,7-diiodo-8-quinolinol (DIQ).

Therefore, the aim of this study was to investigate how cultivar, iodine biofortification, and culinary thermal processing jointly shape the phenolic profile of kale. Green- and red-leaved cultivars, biofortified with KIO_3_ or 5,7-diiodo-8-quinolinol (DIQ), were compared with non-biofortified controls after blanching, boiling, and steaming using LC–HRMS-based phenolic profiling. Particular emphasis was placed on distinguishing processing-related variation from differences associated with the iodine-treatment pools and on characterizing whether processing-related patterns in individual phenolic compounds differed among Control, KIO_3_, and DIQ samples in the two cultivars. This approach provides a compound-resolved assessment of phenolic responses in iodine-biofortified kale under culinary processing conditions relevant to consumption.

## 2. Results and Discussion

### 2.1. LC–HRMS Characterization and Annotation of Phenolic Compounds

**Figure 1:**
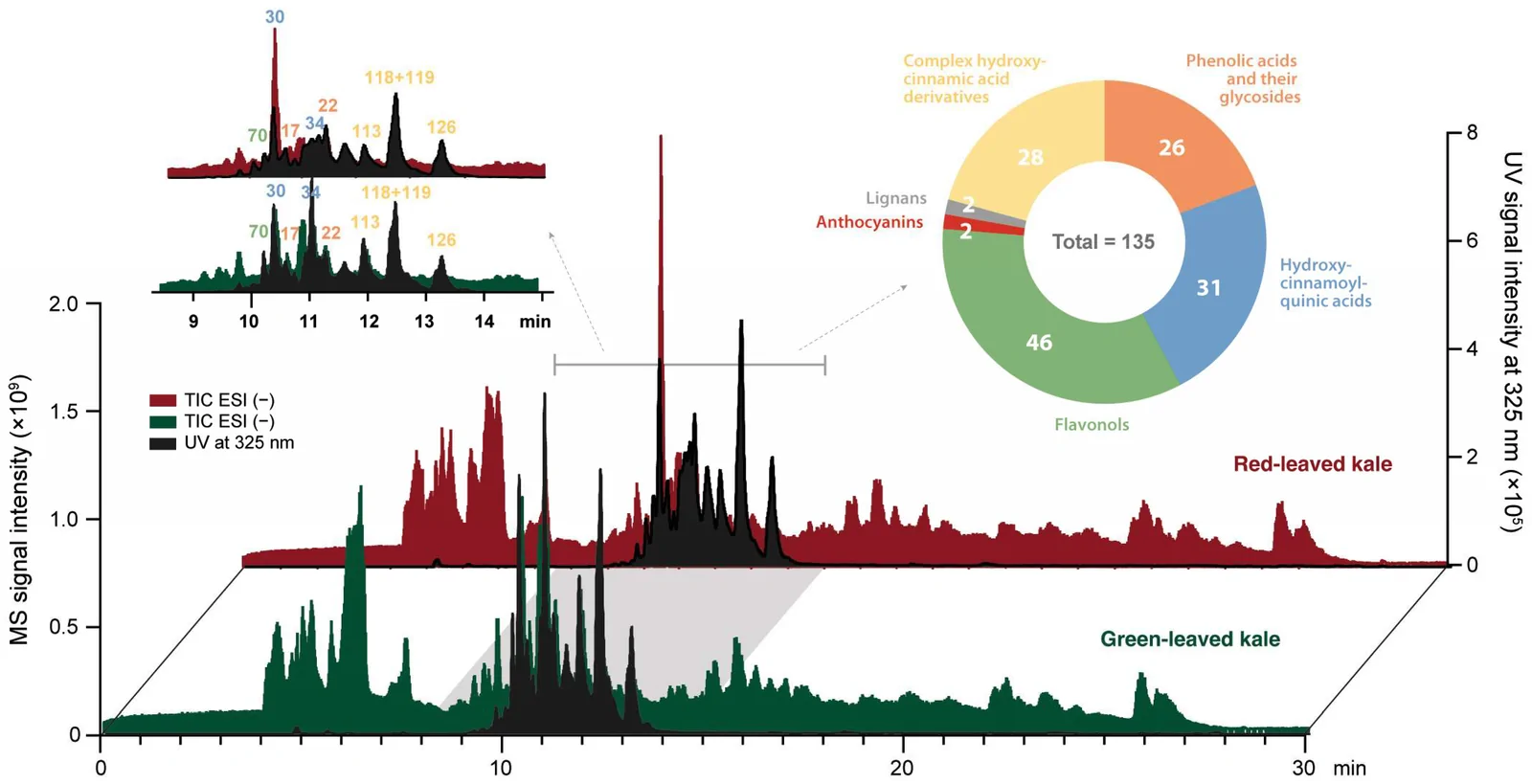
Representative total ion chromatograms (TICs) and UV chromatographic profiles, together with the distribution of annotated phenolic compounds in green- and red-leaved kale. TICs were acquired by LC–HRMS in negative electrospray ionization mode (ESI−), while UV chromatograms were recorded at 325 nm. The enlarged chromatographic region shows the corresponding TIC and UV traces, with numbered peaks indicating compounds selected for subsequent quantitative and semi-quantitative determination based on PDA detection. The donut chart shows the distribution of the 135 annotated compounds among six phenolic classes. Compound numbers correspond to those listed in Table 1 and Supplementary Table S1.

Phenolic profiling by LC–HRMS in negative ionization mode resulted in the annotation of 135 compounds, classified into six major groups: phenolic acids and their glycosides (26), hydroxycinnamoylquinic acid derivatives (31), flavonols (46), anthocyanins (2), lignans (2), and complex hydroxycinnamic acid derivatives (28) (Table 1, Figure 1). Representative LC–MS chromatograms of green- and red-leaved kale illustrate the overall complexity of the phenolic profiles, while the corresponding PDA chromatograms highlight the major chromatographic peaks subsequently selected for quantitative and semi-quantitative analysis.

**Figure 2:**
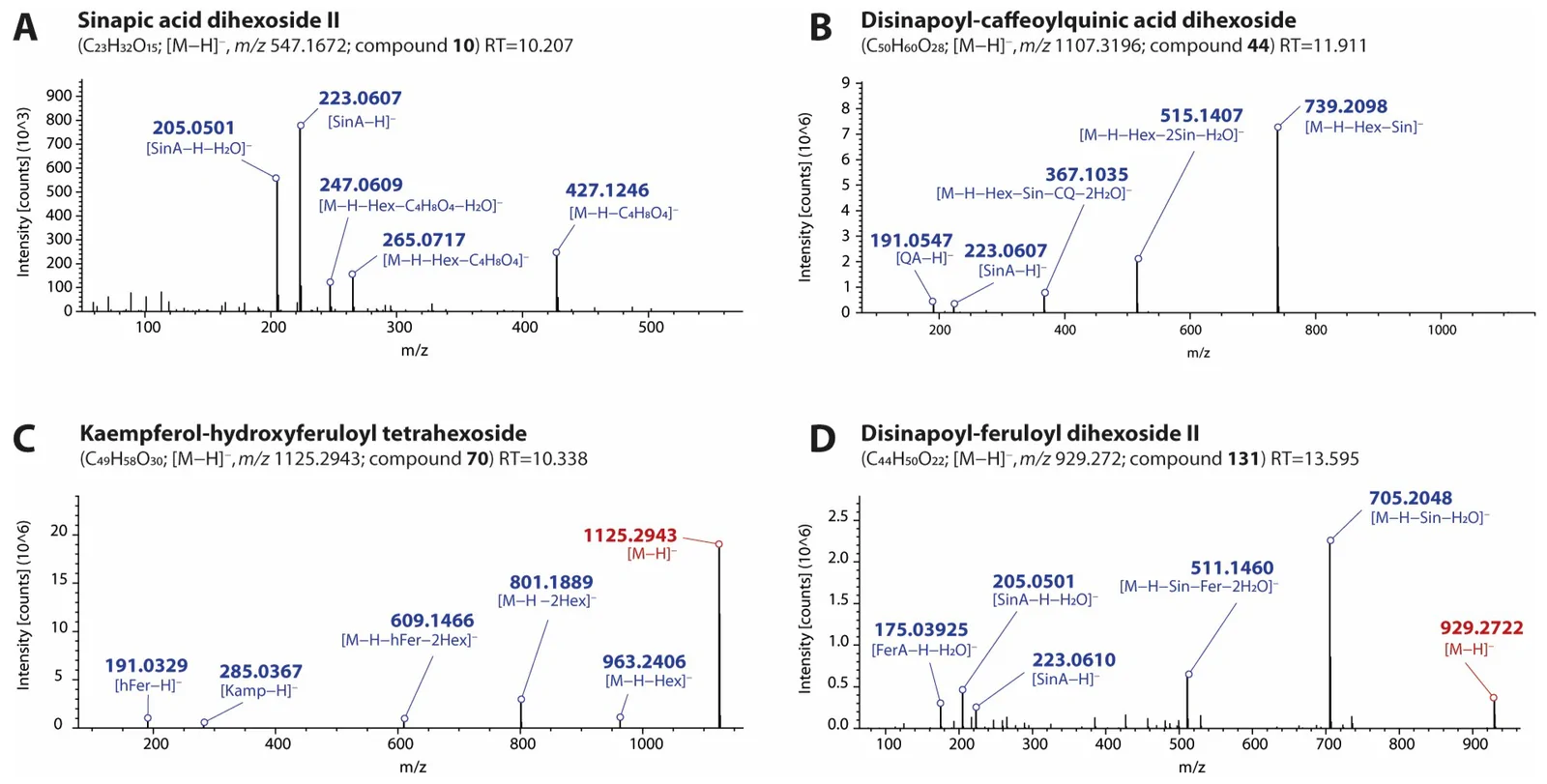
Representative HRMS/MS spectra supporting the annotation of selected phenolic compounds in kale: (**A**) sinapic acid dihexoside II (compound 10), (**B**) disinapoyl-caffeoylquinic acid dihexoside (compound 44), (**C**) kaempferol-hydroxyferuloyl tetrahexoside (compound 70), and (**D**) disinapoyl-feruloyl dihexoside II (compound **131**). Diagnostic product ions and their proposed assignments are indicated. Spectra were acquired in negative ionization mode using HCD at a normalized collision energy of 30. Complete MS/MS data and proposed fragment assignments are provided in Supplementary Table S1.

Compound annotation was based on accurate mass measurements, isotopic patterns, diagnostic MS/MS product ions and neutral losses, supported by comparison with literature data. Three compounds—sinapic acid, neochlorogenic acid, and chlorogenic acid—were confirmed using authentic reference standards and assigned identification confidence Level 1 according to Schymanski et al. (2014) [10]. Most remaining compounds were assigned Level 2b based on diagnostic HRMS/MS evidence, whereas eight compounds for which the available fragmentation data did not permit sufficiently specific structural assignment were retained at Level 3. Detailed precursor and product ion data, proposed fragment assignments, identification confidence levels, and supporting literature references are provided in Supplementary Table S1. Representative HRMS/MS spectra illustrating the diagnostic product ions used for annotation of selected compounds from the major phenolic groups are presented in Figure 2.

Phenolic acids and their glycosides (compounds **1**–**26**) were characterized by neutral losses corresponding predominantly to hexose (−162.053 Da) and pentose (−132.042 Da), yielding product ions attributable to the respective phenolic acid moieties. Additional losses of CO_2_ (−44 Da), CH_3_ (−15 Da), and H_2_O (−18 Da) provided complementary structural information. Sequential sugar losses supported the assignment of mono- and diglycosylated derivatives, while diagnostic aglycone-related ions allowed differentiation among caffeic, ferulic, sinapic, and *p*-coumaric acid derivatives.

Hydroxycinnamoylquinic acid derivatives (compounds **27**–**57**) constituted a structurally diverse group, including mono-, di-, and tri-acylated conjugates. Their annotation relied on characteristic ions and neutral losses associated with quinic acid and hydroxycinnamoyl residues, including caffeoyl, feruloyl, sinapoyl, and *p*-coumaroyl moieties. Sequential combinations of these losses with those of sugar residues provided evidence for the proposed highly substituted structures. Such structural complexity is consistent with the extensive occurrence of hydroxycinnamic acid conjugates previously reported in kale [11,12,13].

Flavonols represented the largest group of annotated phenolics (compounds **58**–**103**). Their MS/MS spectra were characterized by product ions corresponding to kaempferol, quercetin, and isorhamnetin following sequential losses of sugar and acyl residues. Neutral losses of hexose (−162 Da), together with losses corresponding to caffeoyl, feruloyl, hydroxyferuloyl, and sinapoyl moieties, supported the annotation of numerous highly glycosylated and acylated derivatives. This structural diversity is consistent with previous detailed LC–MS studies of kale. Schmidt et al. (2010) [13], for example, characterized 71 flavonol glycosides derived from quercetin, kaempferol, and isorhamnetin, including 44 compounds acylated with p-coumaric, caffeic, ferulic, hydroxyferulic, or sinapic acid residues, whereas Olsen et al. (2010) [12] reported 47 acylated and non-acylated flavonoid glycosides and complex hydroxycinnamic acid derivatives in the red-leaved cultivar ‘Redbor F1’. Two features (compounds **87** and **88**) showed molecular formulas differing by +18.011 Da from the corresponding kaempferol–sinapoyl and kaempferol–feruloyl trihexosides (compounds **82** and **83**, respectively). Their MS/MS spectra included loss of H_2_O (−18.011 Da) and yielded product ions consistent with those of the corresponding lower-mass compounds. They were therefore conservatively assigned at Level 3 as putative hydrated forms rather than definitive structures.

Two anthocyanin derivatives (compounds **104** and **105**) were annotated based on characteristic product ions related to the cyanidin moiety and sequential losses of sugar and acyl residues. Diagnostic neutral losses included hexose (−162 Da), sinapoyl (−206 Da), and feruloyl (−176 Da) moieties, supporting the assignment of acylated cyanidin glycosides. The occurrence of structurally complex acylated anthocyanins is consistent with previous reports on the phenolic composition of red kale, including the ‘Redbor’ cultivar [12].

Two lignan glycosides (compounds **106** and **107**) were annotated based on sequential losses of hexose residues followed by characteristic fragmentation of the lignan aglycone. Lariciresinol dihexoside (**106**) showed particularly strong literature support, as a corresponding compound with matching precursor mass and sequential fragmentation has recently been reported in cruciferous vegetables by Baky et al. (2025) [14]. Pinoresinol dihexoside (**107**) was supported by previously reported lignan fragmentation data [15].

Complex hydroxycinnamic acid derivatives (compounds **108**–**135**) were characterized by the presence of multiple hydroxycinnamoyl residues conjugated to sugar moieties. Their MS/MS spectra showed sequential losses of hexose units together with diagnostic losses corresponding to sinapoyl, feruloyl, hydroxyferuloyl, and *p*-coumaroyl residues. The occurrence of several diagnostic product ions within individual MS/MS spectra supported the annotation of highly substituted conjugates containing two or three hydroxycinnamic acid-derived moieties. Owing to their structural complexity and the presence of multiple possible positional isomers, assignments were based primarily on the composition of the conjugates and their diagnostic fragmentation patterns rather than on unequivocal localization of individual substituents.

Overall, the present LC–HRMS workflow revealed substantial structural diversity within the kale phenolic profile, particularly among highly glycosylated and acylated flavonols and complex hydroxycinnamic acid derivatives. This complexity is consistent with previous detailed studies of kale phenolics. Olsen et al. (2009) [16] tentatively identified 32 phenolic compounds in curly kale, whereas Lin and Harnly (2009) [11] reported an extensive range of flavonoids and hydroxycinnamic acid derivatives in kale and related *Brassica* vegetables. Subsequent structure-oriented studies demonstrated an even greater diversity, including 71 flavonol glycosides in different kale cultivars [13] and 47 acylated and non-acylated flavonoid glycosides and complex hydroxycinnamic acid derivatives in the red-leaved cultivar ‘Redbor F1’ [12]. The broader coverage obtained in the present study may partly reflect the analysis of two cultivars across multiple biofortification and processing conditions, as well as the high-resolution MS/MS workflow and inclusion of chromatographically resolved putative isomeric features. Nevertheless, direct comparison of the numbers of annotated compounds among studies should be made cautiously because extraction procedures, chromatographic resolution, MS acquisition strategies, analytical scope, and annotation criteria differ substantially. Accordingly, the 135 annotations reported here should not be interpreted as 135 unequivocally identified chemical structures: three compounds were confirmed at Level 1, most annotations represented probable structures (Level 2b), and eight were retained as tentative structural candidates (Level 3).

### 2.2. Global Variation in Phenolic Profiles Across Cultivars, Thermal Processing and Iodine Treatments

**Figure 3:**
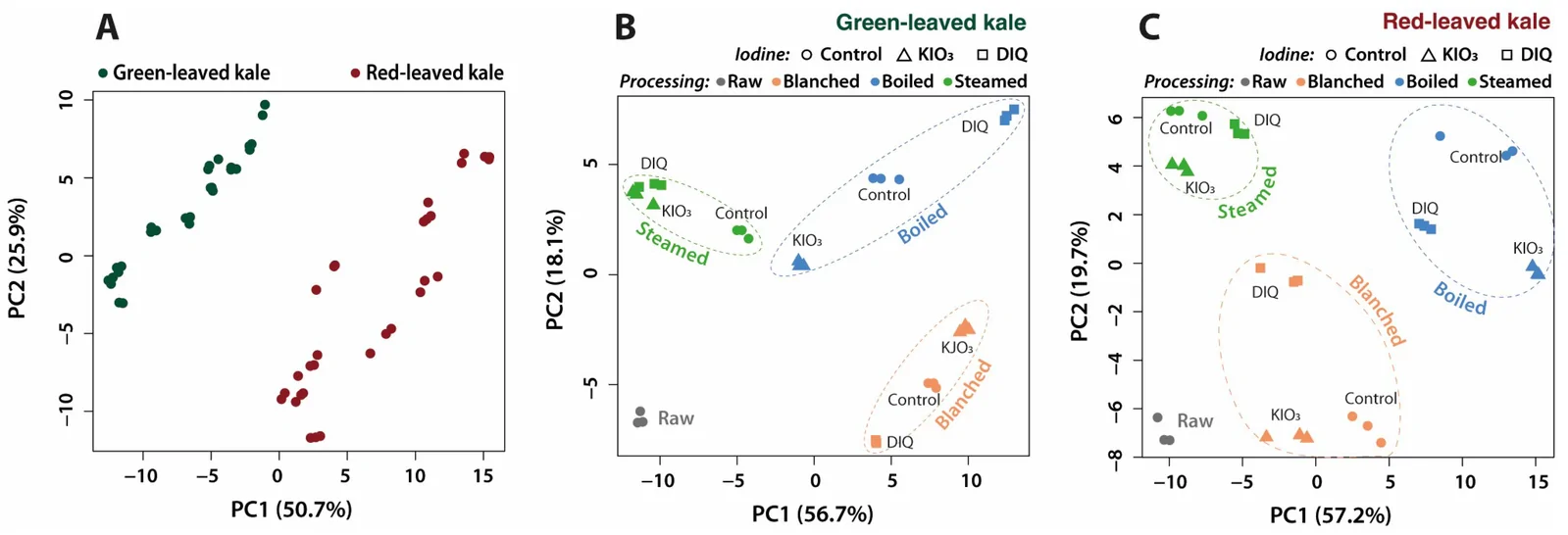
Principal component analysis (PCA) of LC–HRMS phenolic profiles of green- and red-leaved kale subjected to iodine biofortification and thermal processing. (**A**) PCA of the complete dataset showing the overall separation between green- and red-leaved kale. PCA performed separately for (**B**) green-leaved and (**C**) red-leaved kale. In panels B and C, colours indicate thermal processing conditions (raw, blanched, boiled, and steamed), whereas symbols indicate iodine treatment (Control, KIO_3_, and DIQ). Raw samples represent untreated controls. Dashed outlines were added as visual guides to facilitate recognition of samples subjected to the same thermal processing treatment and do not represent statistical confidence regions. PC1 and PC2 percentages indicate the proportion of total variance explained by each principal component.

To evaluate global variation in phenolic profiles across kale cultivars and treatments, principal component analysis (PCA) was performed using the LC–HRMS dataset. PCA of the complete dataset revealed a clear separation between green- and red-leaved kale (Figure 3A), with the first two principal components explaining 50.7% and 25.9% of the total variance, respectively. This separation indicates pronounced cultivar-related differences in phenolic composition and is consistent with previous studies demonstrating substantial genotype-dependent variation in the phytochemical composition of kale and other *Brassica* vegetables [17,18]. Such cultivar dependence is particularly relevant when evaluating responses to external treatments, because differences in the initial phytochemical composition may influence the magnitude and direction of subsequent processing- or biofortification-associated changes.

When the cultivars were analysed separately, treatment-related variation became more apparent (Figure 3B,C). In green-leaved kale, PC1 and PC2 explained 56.7% and 18.1% of the variance, respectively, whereas in red-leaved kale they accounted for 57.1% and 19.7%. In both cultivars, samples showed clear processing-related structuring, while additional variation associated with iodine treatment was apparent within processing groups. These PCA patterns suggested that thermal processing was a major source of within-cultivar variation; however, PCA alone does not provide a formal partitioning of the effects attributable to processing, iodine treatment, and their interaction.

Therefore, the relative contributions of thermal processing, iodine treatment, and their interaction to multivariate phenolic variation were evaluated separately for each cultivar using variance partitioning based on the PERMANOVA model. Because raw samples were available only for untreated controls, the analysis was restricted to the complete 3 × 3 subset comprising three processing treatments (blanching, boiling, and steaming) and three iodine treatments (Control, KIO_3_, and DIQ). In green-leaved kale, thermal processing accounted for the largest proportion of multivariate variation (R^2^ = 0.616), whereas iodine treatment accounted for 9.3% (R^2^ = 0.093) and the Processing × Iodine component accounted for a further 24.2% (R^2^ = 0.242). A similar pattern was observed in red-leaved kale, where thermal processing explained 68.5% of the multivariate variation (R^2^ = 0.685), compared with 7.2% for iodine treatment (R^2^ = 0.072) and 19.6% for the Processing × Iodine component (R^2^ = 0.196). Because the processing replicates within each cultivar × iodine combination were derived from the same pooled plant material, these R^2^ values are interpreted as descriptive measures of variance partitioning within the present dataset rather than as inferential tests of biologically replicated iodine effects. Full variance-partitioning results are provided in Table S2.

Thus, within the complete factorial subset of processed samples, thermal processing represented the largest source of multivariate variation in the phenolic profiles of both cultivars. Notably, the Processing × Iodine component accounted for a larger proportion of variation than the main iodine component in both cultivars. This pattern indicates that differences among the iodine-treatment pools were not expressed uniformly across processing conditions, but varied according to the subsequent thermal treatment. These observations are consistent with the processing-related patterns apparent in the PCA and with previous reports showing cultivar- and treatment-dependent responses of iodine-biofortified kale to culinary processing. Previous studies using KIO_3_ and iodine-containing organic compounds reported treatment- and cultivar-dependent differences in iodine retention, total polyphenol content, and antioxidant properties following boiling, blanching, and steaming [8,19,20]. However, these studies primarily evaluated bulk compositional or antioxidant parameters rather than compound-resolved phenolic profiles. The present LC–HRMS data extend these observations by showing that differences associated with iodine treatment and thermal processing are detectable across the multivariate phenolic profile, while the lack of independent biological replication of the iodine treatments limits population-level inference regarding iodine effects and their interaction with processing.

**Figure 4:**
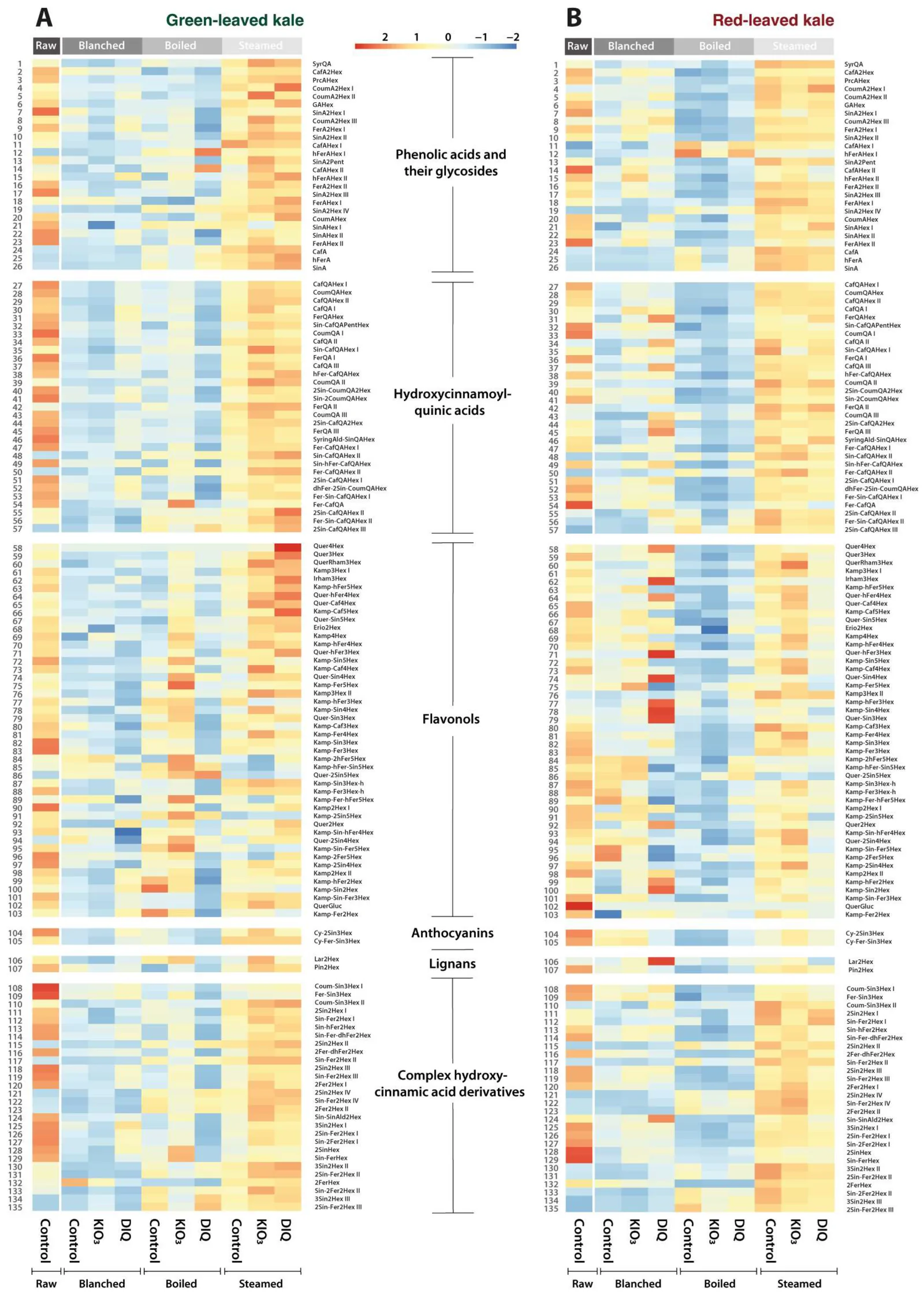
Heatmaps of LC–HRMS phenolic profiles in green- and red-leaved kale subjected to iodine biofortification and thermal processing. Heatmaps are shown separately for (**A**) green-leaved and (**B**) red-leaved kale. Rows correspond to the 135 annotated phenolic compounds, grouped according to phenolic class, whereas columns represent the experimental treatments. Within each processing condition, samples are arranged according to iodine treatment (Control, KIO_3_, and DIQ); raw samples represent untreated controls. Heatmaps were generated from mean LC–HRMS signal intensities of three independently processed portions and autoscaled by row. The colour scale therefore represents the autoscaled signal intensity of each compound across treatments.

The compound-level patterns underlying these global effects are visualized in the heatmaps shown in Figure 4.

The heatmaps, generated separately for green- and red-leaved kale from the mean LC–HRMS signal intensities of three independently processed portions and autoscaled by compound, revealed extensive but heterogeneous responses across the 135 annotated phenolics. Processing-related changes were apparent across several phenolic classes, particularly among hydroxycinnamic acid derivatives, flavonols, and complex hydroxycinnamic acid conjugates. Differences among Control, KIO_3_, and DIQ samples within individual processing treatments were also evident for subsets of compounds, consistent with the descriptive variance-partitioning results showing a substantial Processing × Iodine component. Hierarchical clustering of individual phenolic features further illustrated groups of compounds showing similar treatment-associated response patterns in both cultivars (Figure S1).

Overall, the multivariate analyses showed that thermal processing accounted for the largest proportion of variation among the factorially balanced processed samples. Differences among the Control, KIO_3_, and DIQ source pools and the relatively large Processing × Iodine component of the variance partitioning indicate that these between-pool patterns were not uniform across processing conditions. However, because each iodine treatment within a cultivar was represented by a single pooled biological source, these patterns are interpreted descriptively and do not provide inferential evidence of iodine treatment effects or their interaction with processing. The compound-specific patterns apparent in the heatmaps provide the basis for the subsequent analyses of processing effects and descriptive iodine-associated differences.

### 2.3. Effects of Thermal Processing and Iodine Treatment on Phenolic Classes

**Figure 5:**
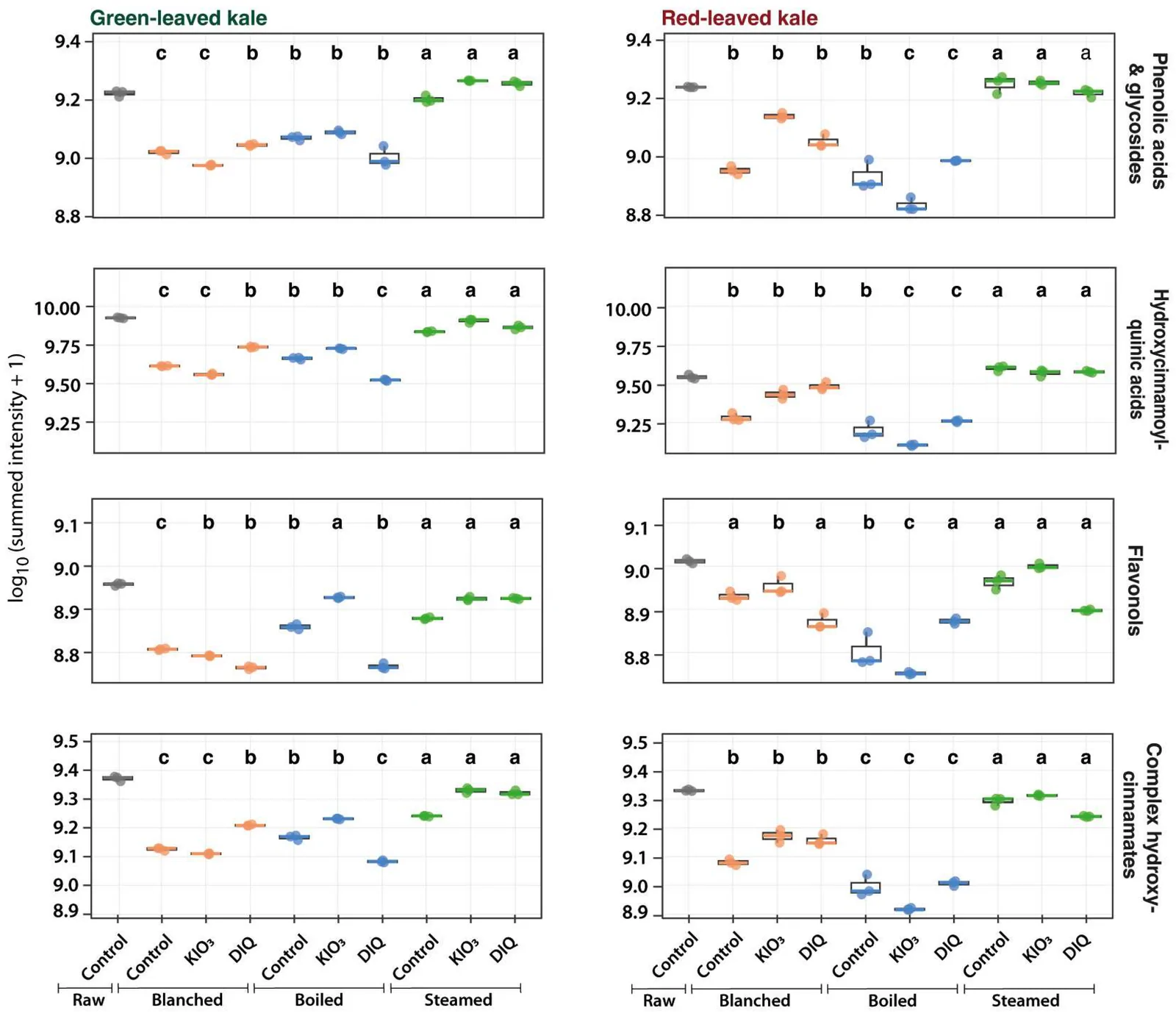
Effects of thermal processing on major phenolic classes in green- and red-leaved kale under different iodine treatments. Box plots show log_10_-transformed summed LC–HRMS signal intensities [log_10_(summed intensity + 1)] for four major phenolic classes under different thermal processing and iodine treatments. Boxes indicate interquartile ranges with median values, and points represent independently processed portions derived from the same pooled plant material (*n* = 3). For each cultivar, phenolic class, and iodine treatment (Control, KIO_3_, or DIQ), the effect of thermal processing was evaluated separately by one-way ANOVA across blanched, boiled, and steamed samples, followed by Tukey-adjusted post-hoc comparisons (*p* < 0.05). Different lowercase letters indicate significant differences among processing treatments within the same iodine treatment only; letters should not be compared across Control, KIO_3_, and DIQ groups. Raw controls are shown as descriptive reference values and were not included in the statistical analyses.

To examine how treatment effects were reflected at the phenolic-class level, summed LC–HRMS signal intensities were evaluated separately for four predominant phenolic classes: phenolic acids and their glycosides, hydroxycinnamoylquinic acids, flavonols, and complex hydroxycinnamic acid derivatives (Figure 5). Relative summed LC–HRMS signal intensities, expressed as percentages of the corresponding raw control (Raw Control = 100%), are provided in Supplementary Table S3.

Class-level profiles showed pronounced processing-related differences in both cultivars, although the magnitude of these changes varied among phenolic classes and treatment combinations. In untreated green-leaved kale, summed signals after steaming corresponded to 73.98–95.40% of the raw-control values across the four classes, whereas boiling resulted in lower relative signals of 54.52–79.74%. In iodine-treated samples, steamed green kale showed relative summed signals ranging from 90.94 to 110.50% of the raw-control values. In red-leaved kale, boiling was likewise associated with the lowest relative signals, ranging from 45.12 to 82.91% in untreated samples, whereas steamed untreated samples ranged from 89.61 to 113.93% of the corresponding raw-control values.

Analysis of the processed samples showed significant effects of thermal processing on all four phenolic classes in both cultivars, although the magnitude and pattern of these differences varied among iodine treatments. Because the processing replicates within each iodine treatment were derived from the same pooled plant material, the effect of thermal processing was evaluated separately within each iodine treatment rather than by factorial comparison of iodine treatments. One-way ANOVA showed a significant overall effect of processing (*p* < 0.05) for all phenolic classes within each cultivar and iodine treatment. Tukey-adjusted post-hoc comparisons generally indicated higher summed phenolic signals after steaming than after boiling, although the pattern of pairwise differences varied among phenolic classes and iodine treatments (Figure 5). For example, in green-leaved kale, steamed samples showed significantly higher flavonol signals than blanched samples under all three iodine treatments, whereas steamed and boiled samples did not differ significantly under KIO_3_ treatment. In red-leaved kale, processing-related differences in flavonols were less consistent; under DIQ treatment, the overall processing effect was significant (*p* = 0.045), but no individual pairwise comparison remained significant after Tukey adjustment. Differences among Control, KIO_3_, and DIQ groups are therefore presented descriptively and were not subjected to inferential statistical testing.

The overall tendency towards higher phenolic-class signals after steaming than after boiling is consistent with previous studies of kale. Murador et al. (2016) [21] reported higher extractable phenolic contents after steaming than after boiling, whereas Armesto et al. (2016) [22] similarly observed greater losses of total phenolics during boiling than during steaming in Galega kale. Studies examining individual phenolic constituents have further demonstrated that the response to thermal processing may differ considerably among compounds, indicating that changes in bulk phenolic content can reflect heterogeneous responses of individual metabolites. These observations are consistent with the present results, in which the general processing-related pattern was apparent across phenolic classes but its magnitude varied among classes, cultivars, and iodine treatments.

Several concurrent processes may contribute to these differences. In particular, boiling allows direct transfer of water-soluble phenolic constituents from the plant tissue into the cooking medium. Studies of leafy *Brassica* vegetables have demonstrated substantial leaching during boiling; notably, Giambanelli et al. (2016) [23] showed that changes measured in cooked plant tissue did not necessarily reflect losses from the complete food–water system when the cooking water was also considered. Reduced direct contact with water during steaming may therefore contribute to the generally higher phenolic signals observed in steamed kale. Thermal processing may additionally affect phenolic compounds through degradation or transformation and through changes in their extractability from the plant matrix [2]. However, the relative contribution of these processes cannot be determined from the present data. Moreover, steaming and boiling differed not only in direct contact with water but also in treatment duration (15 and 20 min, respectively); consequently, the observed differences cannot be attributed exclusively to the processing mode.

The present compound-class results also extend our previous observations obtained from bulk measurements in the same experimental system, in which thermal processing and iodine biofortification affected total polyphenol content in a cultivar- and treatment-dependent manner [8,19,20]. The LC–HRMS data reported here show that these overall responses involve multiple phenolic classes and that processing-related patterns differed among the Control, KIO_3_, and DIQ sample pools. Because independent biological replication of the iodine treatments was not available after pooling, these between-treatment differences should be interpreted descriptively.

Importantly, the LC–HRMS data represent relative extractable signal intensities rather than absolute phenolic concentrations, and the cooking water was not analysed. Consequently, decreases in signal cannot be interpreted directly as compound loss, nor can higher signals after steaming be considered evidence of greater absolute phenolic retention. Nevertheless, the consistent differences among processing treatments demonstrate that thermal treatment substantially modifies the extractable phenolic-class profiles of kale. The magnitude of these processing-related differences varied among the Control, KIO_3_, and DIQ sample pools; however, because each iodine treatment within a cultivar was represented by a single pooled biological source, these between-treatment patterns are interpreted descriptively rather than as inferential evidence of an iodine × processing interaction.

### 2.4. Compound-Level Effects of Thermal Processing

To further resolve the processing-related changes observed at the phenolic-class level, individual phenolic compounds were evaluated by comparing steamed and boiled samples. For each compound, the steamed-versus-boiled contrast was estimated within each of the three pooled source materials corresponding to the Control, KIO_3_, and DIQ treatments and then averaged across these pools to characterize the overall processing-related difference. Because each iodine treatment within a cultivar was represented by a single pooled biological source, this analysis was restricted to inference on the processing contrast and was not used to test iodine effects or Processing × Iodine interactions. Raw samples were not included because iodine-treated raw samples were not available.

**Figure 6:**
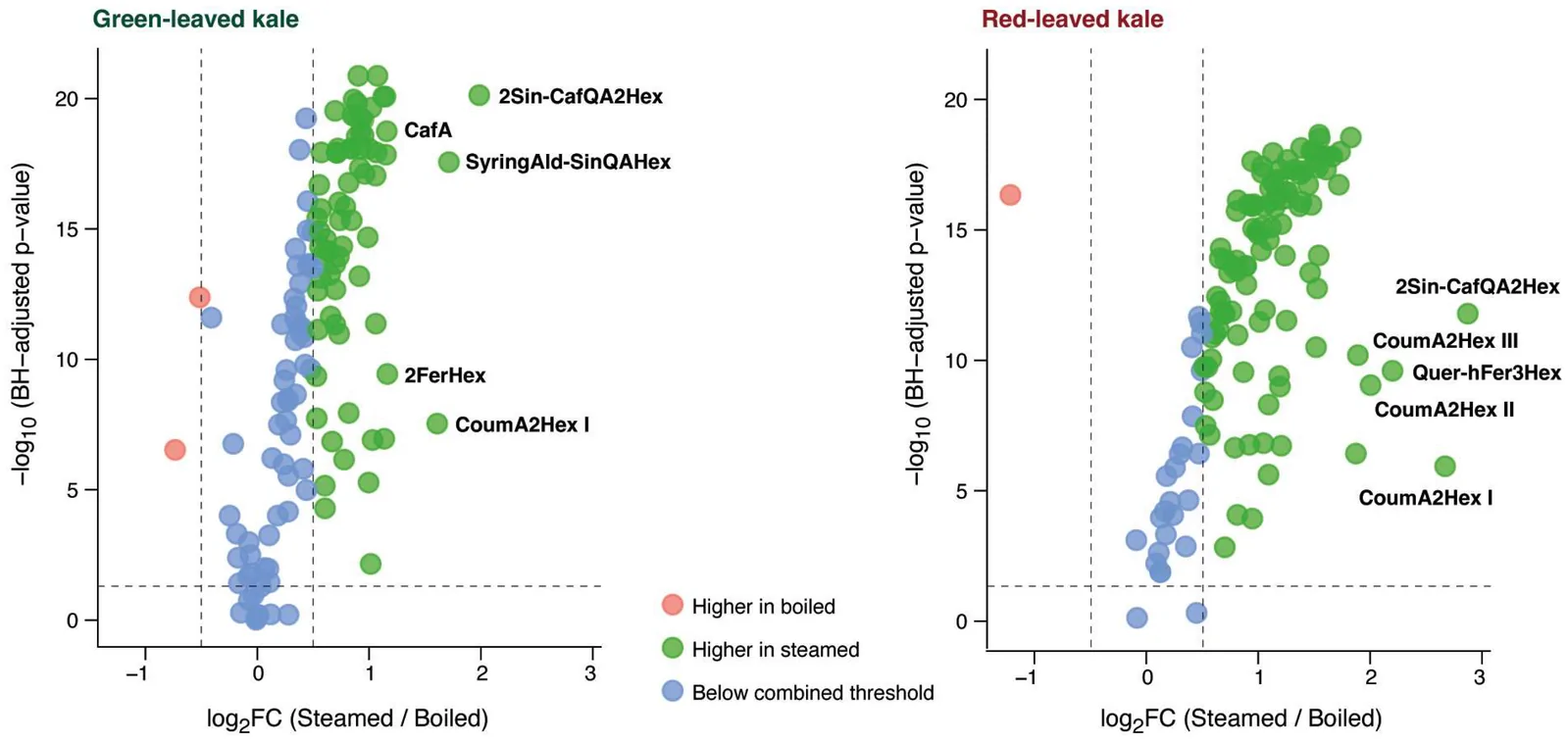
Compound-level differences between steamed and boiled kale phenolic profiles. Volcano plots show the overall steamed-versus-boiled processing contrast for individual phenolic compounds in green- and red-leaved kale. For each compound, the steamed-versus-boiled contrast was estimated within each of the three pooled source materials corresponding to the Control, KIO_3_, and DIQ treatments and then averaged across these pools. Positive log_2_FC values indicate higher relative LC–HRMS signal intensities in steamed samples, whereas negative values indicate higher signals in boiled samples. *p*-values for the processing contrast were adjusted separately within each cultivar across the 135 compounds using the Benjamini–Hochberg false discovery rate (FDR) procedure. Compounds with BH-adjusted *p* < 0.05 and |log_2_FC| ≥ 0.5 are highlighted. The fold-change threshold was used as an effect-size criterion for visualization. Because each iodine treatment within a cultivar was represented by a single pooled biological source, this analysis evaluates the overall processing contrast across the three source pools and does not provide inferential tests of iodine treatment or Processing × Iodine interaction.

The steamed-versus-boiled contrast revealed widespread differences in relative LC–HRMS signal intensities in both cultivars. After Benjamini–Hochberg correction, 125 of 135 compounds in green-leaved kale and 133 of 135 compounds in red-leaved kale showed a significant overall difference between the two processing treatments (p_adj < 0.05; Table S4). When an additional effect-size criterion of |log_2_FC| ≥ 0.5 was applied, 73 compounds in green-leaved kale and 110 compounds in red-leaved kale met both criteria (Table S4; Figure 6). The effect-size threshold was used to facilitate prioritization and visualization of the most pronounced compound-level processing differences rather than as an additional inferential test.

The direction of the steamed-versus-boiled contrast was strongly skewed towards positive log_2_FC values (Table S4; Figure 6). In green-leaved kale, 118 of 135 compounds showed positive log_2_FC values, with a median log_2_FC of 0.542, whereas only 17 compounds showed negative values. This directional pattern was even more pronounced in red-leaved kale, where 132 of 135 compounds showed positive log_2_FC values and the median log_2_FC reached 0.954. The overall log_2_FC ranges were −0.734 to 1.985 and −1.221 to 2.867 for green- and red-leaved kale, respectively. Thus, although compound-specific responses varied considerably, relative LC–HRMS signals were generally higher after steaming than after boiling, particularly in the red-leaved cultivar.

The largest overall steamed-versus-boiled differences were observed for structurally diverse phenolic metabolites rather than for a single phenolic subclass. In green-leaved kale, the five compounds with the largest absolute log_2_FC values were 2Sin-CafQA2Hex, SyringAld-SinQAHex, CoumA2Hex I, 2FerHex, and CafA, whereas in red-leaved kale the largest differences were observed for 2Sin-CafQA2Hex, CoumA2Hex I, Quer-hFer3Hex, CoumA2Hex II, and CoumA2Hex III (Figure 6). Notably, all of these highest-ranked responses were positive, indicating higher relative signals after steaming. The occurrence of pronounced responses among hydroxycinnamoylquinic acid derivatives, phenolic acid glycosides, flavonol conjugates, and complex hydroxycinnamic acid derivatives indicates that the processing-associated differences were distributed across several components of the phenolic profile.

The marked compound-to-compound variation is consistent with previous studies showing that individual phenolic constituents of *Brassica* vegetables do not respond uniformly to thermal processing. In Galega kale, Armesto et al. (2019) [24] demonstrated that the effects of boiling, steaming, microwaving, pressure cooking, and vacuum cooking on flavonoid contents depended on both the processing method and the individual compound. Compound-specific thermal responses have also been observed for kale flavonoids under controlled heating conditions [25]. Such variability is particularly relevant to kale because its phenolic profile contains numerous highly glycosylated and acylated quercetin and kaempferol derivatives with substantial structural diversity [26]. The present LC–HRMS results extend these observations to a broader phenolic profile, showing that heterogeneous processing responses encompass not only flavonol conjugates but also hydroxycinnamoylquinic acids, phenolic acid derivatives, and complex hydroxycinnamic acid conjugates.

The predominance of positive steamed-versus-boiled contrasts is consistent with previous observations in kale, where steaming generally resulted in higher extractable phenolic levels than boiling [21,24]. As discussed above, differences between these processing methods may partly result from leaching of water-soluble constituents during boiling, but processing-induced changes in measured phenolic levels may also reflect altered extractability or transformation of phenolic conjugates. Consequently, the higher LC–HRMS signals observed after steaming in the present study should not be interpreted directly as greater absolute retention, particularly because the cooking water was not analysed.

The broader distribution of log_2_FC values and the larger proportion of compounds exceeding the combined significance and effect-size thresholds in red-leaved kale indicate a stronger compound-level differentiation between steamed and boiled samples under the conditions tested. This observation is consistent with the cultivar-specific patterns apparent in the multivariate analyses, but its biochemical basis cannot be established from the present dataset. Differences in the initial phenolic composition of the two cultivars may contribute to the contrasting response patterns; however, attributing them to specific metabolic pathways would require targeted biochemical or molecular evidence.

Overall, the compound-level analysis demonstrates that the processing-related differences observed at the global and class levels were composed of highly heterogeneous responses of individual phenolic metabolites. The predominance of positive steamed-versus-boiled log_2_FC values supports the general pattern observed at the class level, while the wide range of individual effect sizes emphasizes that summed phenolic classes alone do not fully capture the response of the kale phenolic profile to thermal processing.

### 2.5. Iodine-Associated Patterns in Phenolic Responses to Thermal Processing

Although thermal processing represented the largest source of within-cultivar variation in the complete factorial subset, the descriptive variance partitioning also indicated a substantial Processing × Iodine component. This pattern suggested that processing-related differences were not uniform among the Control, KIO_3_, and DIQ source pools and prompted their further descriptive examination at the individual-compound level.

**Figure 7:**
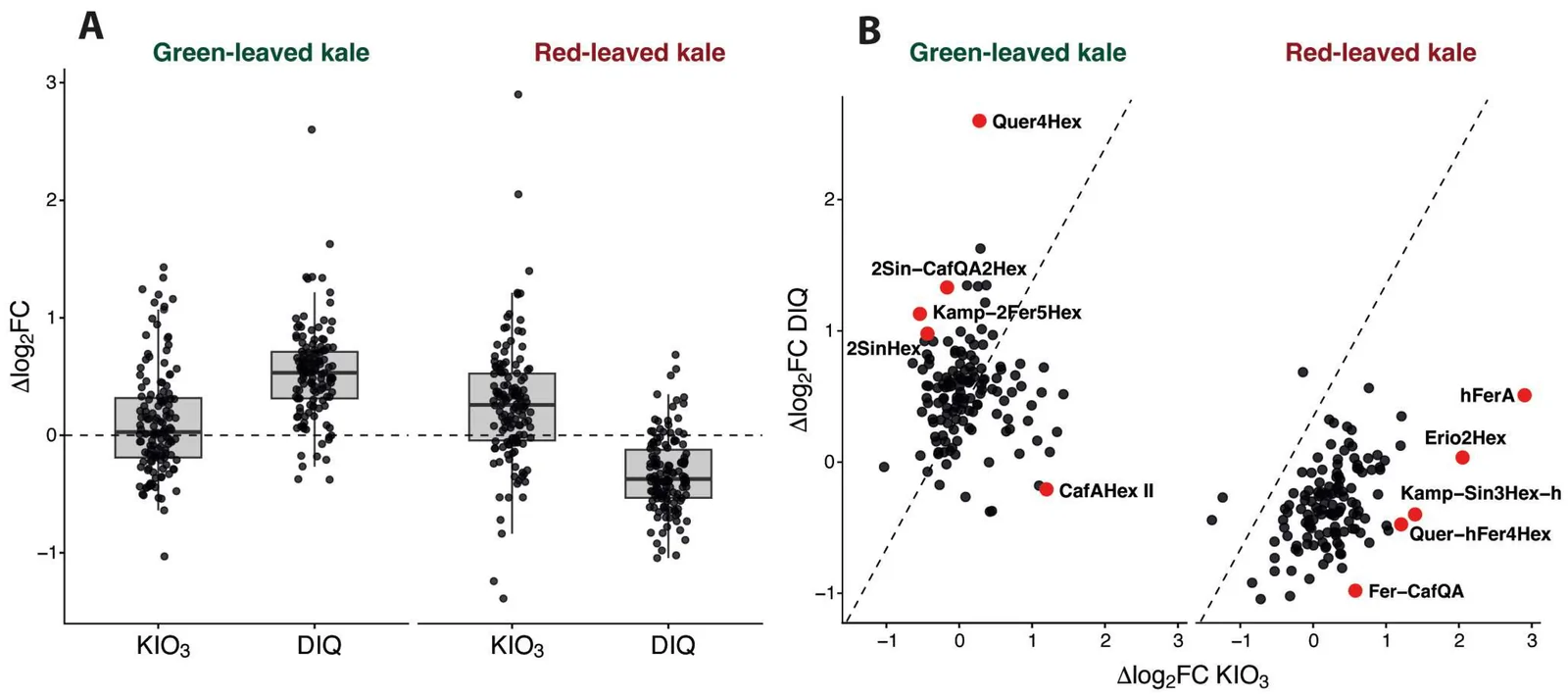
(**A**) Distribution of Δlog_2_FC values for individual phenolic compounds under KIO_3_ and DIQ treatments. For each compound, Δlog_2_FC represents the difference between the steamed-versus-boiled log_2_FC under the respective iodine treatment and the corresponding log_2_FC in the iodine-free control. Box plots show the median and interquartile range, with individual points representing the 135 annotated phenolic compounds. (**B**) Compound-wise comparison of Δlog_2_FC values obtained for KIO_3_ and DIQ. The dashed diagonal line represents equal Δlog_2_FC values for the two iodine treatments (Δlog_2_FC DIQ = Δlog_2_FC KIO_3_). Red points indicate the five compounds showing the largest absolute difference between Δlog_2_FC values for DIQ and KIO_3_ within each cultivar; all remaining compounds are shown in black. Positive Δlog_2_FC values indicate a shift towards a more positive steamed-versus-boiled contrast relative to the iodine-free control, whereas negative values indicate a shift in the opposite direction. The Δlog_2_FC analysis was used descriptively to characterize compound-level response patterns.

For this descriptive comparison, the steamed-versus-boiled log_2_FC calculated for each iodine-treatment pool was compared with the corresponding contrast in the iodine-free control. The resulting Δlog_2_FC therefore represents the difference in the steamed-versus-boiled contrast associated with the KIO_3_ or DIQ pool relative to the untreated control (Figure 7A; Table S5). Positive Δlog_2_FC values indicate a shift towards a more positive steamed-versus-boiled contrast relative to the control, whereas negative values indicate a shift in the opposite direction.

Distinct cultivar- and iodine-treatment-associated patterns were apparent in the distributions of Δlog_2_FC values. In green-leaved kale, the median Δlog_2_FC was close to zero for KIO_3_ (0.027), whereas DIQ showed a more positive median shift (0.533). In contrast, red-leaved kale showed a positive median Δlog_2_FC for KIO_3_ (0.260) and a negative median value for DIQ (−0.372) (Figure 7A). Thus, at the descriptive compound-profile level, the direction of the observed differences varied according to both iodine-treatment pool and cultivar.

Compound-wise comparison of the KIO_3_- and DIQ-associated patterns further illustrated these differences (Figure 7B). In green-leaved kale, most compounds were positioned above the line of equality, indicating generally more positive Δlog_2_FC values under DIQ than under KIO_3_. Conversely, the distribution in red-leaved kale was predominantly shifted below the line of equality, reflecting generally more negative Δlog_2_FC values under DIQ relative to KIO_3_. The compounds showing the largest absolute differences between the two iodine treatments included Quer4Hex, Kamp-2Fer5Hex, 2Sin-CafQA2Hex, 2SinHex, and CafAHex II in green-leaved kale, and hFerA, Erio2Hex, Kamp-Sin3Hex-h, Quer-hFer4Hex, and Fer-CafQA in red-leaved kale (Figure 7B; Table S5). Their distribution on both sides of the equality line in green-leaved kale, but consistently below it in red-leaved kale, further illustrates the cultivar-dependent pattern of differences between the KIO_3_ and DIQ source pools.

The cultivar- and iodine-treatment-associated patterns observed here are consistent with previous studies showing that the chemical composition of iodine-biofortified kale may vary according to both genotype and the chemical form of iodine applied. Studies of ‘Oldenbor F1’ and ‘Redbor F1’ biofortified with KIO_3_ and different iodoquinolines have demonstrated cultivar- and iodine-form-dependent differences in plant chemical composition and antioxidant-related parameters [8]. More recent analyses have likewise reported differences in total polyphenol content and antioxidant capacity according to iodine form and cultivar [20]. These findings provide a broader context for the compound-resolved patterns observed in the present study, in which the Control, KIO_3_, and DIQ sample pools did not show a uniform phenolic response to processing.

The descriptive Δlog_2_FC analysis further showed that processing-related differences were not identical among the Control, KIO_3_, and DIQ sample pools. Previous studies of iodine-biofortified kale have reported that the effects of steaming, blanching, and boiling on the processed product may vary with cultivar and the iodine compound used for biofortification [8,19]. The present LC–HRMS data show a similar pattern at the compound level: the magnitude and, for some compounds, the direction of the steamed-versus-boiled contrast differed among the three source pools. Because each iodine treatment within a cultivar was represented by a single pooled biological source, these differences are interpreted descriptively and do not provide inferential evidence that iodine treatment modified the response to processing.

The biochemical basis of the different patterns observed among the Control, KIO_3_, and DIQ sample pools cannot be established from the present experiment. Iodine biofortification has been associated with changes in antioxidant-related plant metabolism, but the available evidence does not allow the compound-specific responses observed here to be attributed to particular biosynthetic or stress-response pathways. This limitation is particularly relevant for DIQ because, as an organic iodine-containing compound, it differs substantially in chemical form from inorganic KIO_3_. Mechanistic interpretation would therefore require complementary measurements of iodine uptake and speciation together with targeted biochemical or molecular analyses.

Finally, Δlog_2_FC values for individual compounds were used here as descriptive features rather than as independent biological replicates for statistical inference. Accordingly, the distributions in Figure 7 characterize patterns within the detected phenolic profile and should not be interpreted as inferential comparisons between iodine treatments. Taken together, these descriptive patterns indicate that processing-related phenolic responses differed among the Control, KIO_3_, and DIQ source pools and between cultivars, while the absence of independent biological replication of the iodine treatments precludes inferential conclusions regarding iodine effects on the processing response.

### 2.6. Quantitative and Semi-Quantitative Determination of Selected Major Phenolic Compounds

**Figure 8:**
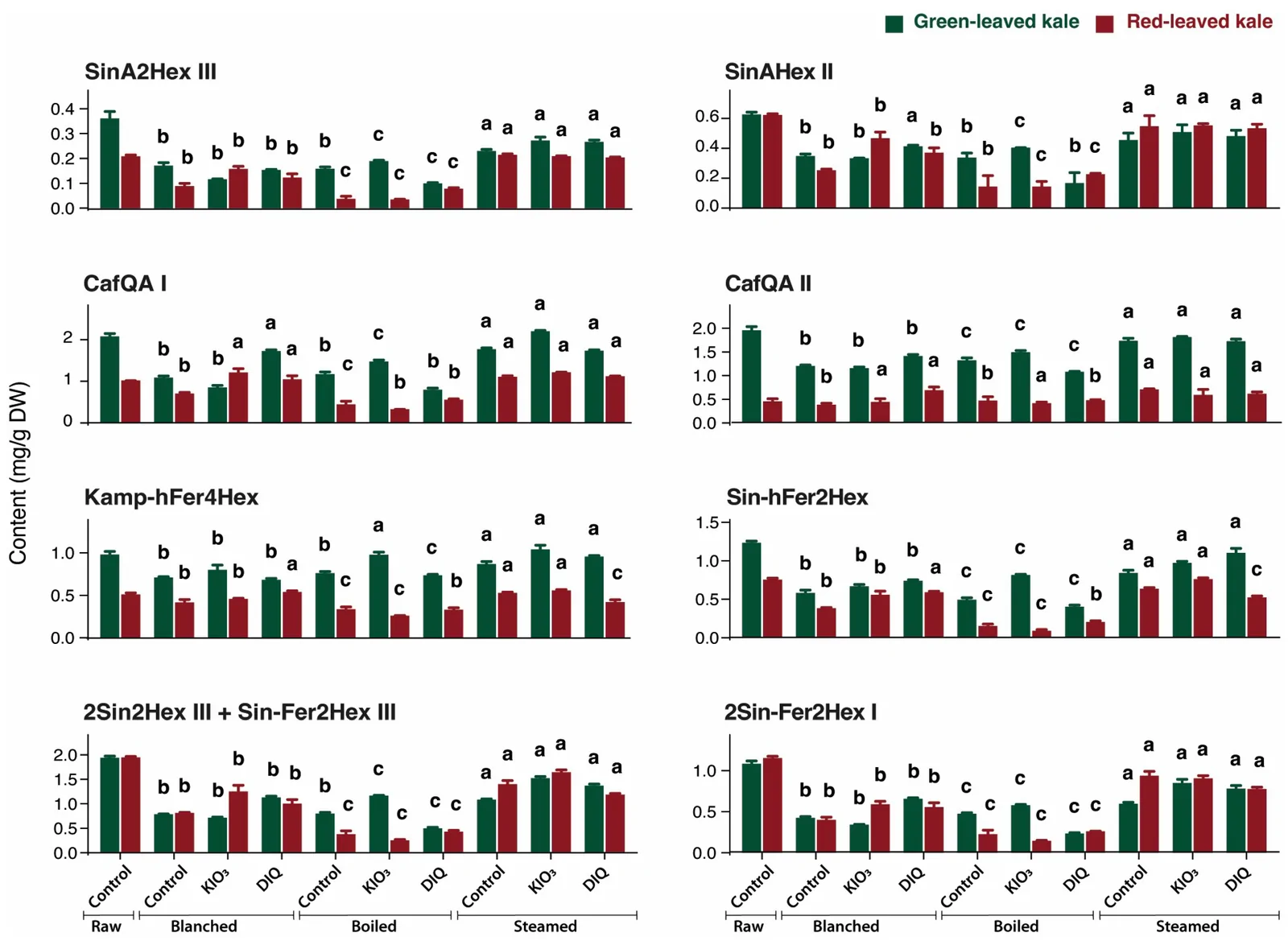
Quantitative and semi-quantitative determination of selected major phenolic compounds in green- and red-leaved kale subjected to different thermal processing and iodine treatments. Values are expressed as mean ± SD (*n* = 3 independently processed portions derived from the same pooled plant material), in mg/g DW. CafQA I and CafQA II were quantified using authentic neochlorogenic acid and chlorogenic acid standards, respectively. Sinapic acid derivatives were semi-quantified as sinapic acid equivalents, whereas Kamp-hFer4Hex was expressed as kaempferol equivalents. Compounds 2Sin2Hex III and Sin-Fer2Hex III co-eluted and were therefore determined together as a single PDA peak. For each cultivar, compound, and iodine treatment (Control, KIO_3_, or DIQ), the effect of thermal processing was evaluated separately by one-way ANOVA across blanched, boiled, and steamed samples, followed by Tukey-adjusted post-hoc comparisons (*p* < 0.05). Different lowercase letters indicate significant differences among processing treatments within the same cultivar and iodine treatment only; letters should not be compared across Control, KIO_3_, and DIQ groups. Raw controls are shown as descriptive reference values and were not included in the statistical analyses.

To complement the relative LC–HRMS profiling with concentration-based data, eight major chromatographic peaks representing selected phenolic compounds were quantified or semi-quantified using PDA detection (Figure 8; Table S7). The selected compounds included sinapic acid derivatives, caffeoylquinic acids, and a kaempferol derivative and represented prominent, well-resolved features of the phenolic profiles. Compounds 118 and 119 co-eluted under the applied chromatographic conditions and were therefore determined together as a single PDA peak.

The concentration-based data generally supported the pronounced effect of thermal processing observed in the untargeted LC–HRMS analysis, while also demonstrating substantial compound- and cultivar-specific variation. In the iodine-free controls, boiling was generally associated with lower contents than steaming for the selected compounds. This pattern was clearly apparent in red-leaved kale. For example, the content of Sin-hFer2Hex decreased from 0.760 mg/g DW in raw leaves to 0.152 mg/g DW after boiling, whereas 0.635 mg/g DW was measured after steaming. Similarly, the combined content of 2Sin2Hex III and Sin-Fer2Hex III was 1.94 mg/g DW in raw red kale, 0.378 mg/g DW after boiling, and 1.40 mg/g DW after steaming. For 2Sin-Fer2Hex I, the corresponding values were 1.15, 0.226, and 0.937 mg/g DW, respectively. These concentration-based results are consistent with the compound-level LC–HRMS analysis, in which the steamed-versus-boiled contrasts were predominantly positive.

The concentrations determined for the selected compounds can be placed within the broader context of phenolic levels previously reported for kale, although direct quantitative comparison is limited by differences in cultivar, growing conditions, extraction procedures, and quantification strategy. Hydroxycinnamic acid derivatives are consistently reported as prominent components of the kale phenolic profile. For example, neochlorogenic acid and chlorogenic acid contents of approximately 0.396 and 0.087 mg/g DW, respectively, have recently been reported in untreated kale [27]. Other studies have likewise reported substantial concentrations of hydroxycinnamic acids and their derivatives in kale, although the reported values vary considerably among cultivars and analytical approaches [28]. In the present study, CafQA I reached 1.02 mg/g DW in raw red-leaved kale and 1.11–1.22 mg/g DW after steaming, illustrating that the abundance of individual caffeoylquinic acid derivatives can also vary markedly with cultivar and treatment.

Sinapic acid-derived compounds also constituted quantitatively important components of the selected PDA profile. This is consistent with the established importance of sinapic acid and its conjugates in kale and other *Brassica* vegetables. Reported concentrations of individual sinapic acid-related constituents vary substantially among studies, reflecting differences in genotype, sample preparation, and whether free, hydrolysed, or conjugated forms are quantified [29]. Direct numerical comparison with the present sinapoyl conjugates is therefore not appropriate because these compounds were semi-quantified as sinapic acid equivalents rather than quantified against their own authentic standards.

Processing-related differences were also observed within the individual Control, KIO_3_, and DIQ sample pools, although their magnitude and pattern varied among compounds, cultivars, and source pools (Figure 8). Because each iodine treatment within a cultivar was represented by a single pooled biological source, differences among the Control, KIO_3_, and DIQ groups were interpreted descriptively rather than subjected to inferential testing. For example, Sin-hFer2Hex in green-leaved kale showed different concentration patterns among the three source pools. After boiling, its content was 0.494 mg/g DW in the Control pool, 0.815 mg/g DW in the KIO_3_ pool, and 0.403 mg/g DW in the DIQ pool, whereas after steaming the corresponding contents were 0.841, 0.973, and 1.10 mg/g DW, respectively. Thus, the differences among the Control, KIO_3_, and DIQ pools were not uniform across processing conditions; however, these patterns should not be interpreted as inferential evidence of an iodine × processing interaction.

Differences between the two cultivars were also apparent descriptively. Several compounds, particularly CafQA II and Kamp-hFer4Hex, showed consistently higher contents in green-leaved than in red-leaved kale, whereas the magnitude of the difference was smaller or varied with treatment for other compounds. Importantly, processing occasionally resulted in contents approaching or exceeding those measured in raw leaves. For example, CafQA I in red-leaved kale reached 1.11 and 1.22 mg/g DW after steaming in the Control and KIO_3_ pools, respectively, compared with 1.02 mg/g DW in raw leaves. Apparent increases in extractable phenolic compounds following thermal processing have also been reported for kale, including after steaming [21]. Such increases should not necessarily be interpreted as de novo formation or improved absolute retention. Thermal disruption of plant tissues and changes in phenolic–matrix interactions may increase extractability, while transformation of structurally related conjugates may also contribute to the measured chromatographic response.

Overall, the PDA-based determinations support the patterns observed in the LC–HRMS profiling while providing concentration-based information for selected major phenolic constituents. Because the cooking water was not analysed and the measurements were expressed per unit of dry weight, the observed differences describe the phenolic composition of the processed plant material rather than mass-balance retention during processing.

## 3. Materials and Methods

### 3.1. Chemicals

Methanol and acetonitrile (LC–MS grade) and formic acid (LC–MS grade) were obtained from Merck (Darmstadt, Germany) and used for extraction and chromatographic analyses. Sinapic acid (>99%) and kaempferol (>97%) were obtained from Sigma-Aldrich (St. Louis, MO, USA), neochlorogenic acid (>98%) from Merck, and chlorogenic acid from PhytoLab (Vestenbergsgreuth, Germany). Ultrapure water (18.2 MΩ·cm) was used for the preparation of aqueous solutions. All other reagents were of at least analytical grade and were used without further purification.

### 3.2. Plant Cultivation, Iodine Biofortification, Thermal Processing, and Sample Preparation

Curly kale (*Brassica oleracea* var. *sabellica* L.) cultivars ‘Oldenbor F1’ (green-leaved) and ‘Redbor F1’ (red-leaved) were grown hydroponically in spring 2022 using a Nutrient Film Technique (NFT) system at the Faculty of Biotechnology and Horticulture, University of Agriculture in Kraków, Poland, as described previously [19]. The nutrient solution was maintained at pH 5.70 and an electrical conductivity of 2.0 mS/cm. Plants were grown under three iodine treatments: an untreated control and supplementation with either KIO_3_ or 5,7-diiodo-8-quinolinol (DIQ), each applied at a concentration of 10 µM. Iodine treatment was initiated at the 4–5 true-leaf stage and continued until harvest at the 13–14 true-leaf stage. The two iodine compounds were applied at the same molar concentration but were not matched for elemental iodine content; DIQ contains two iodine atoms per molecule, whereas KIO_3_ contains one. Further details of the cultivation and biofortification conditions have been reported previously [19].

Thermal processing was performed according to the previously described procedure, with the principal experimental conditions reported here to ensure reproducibility. On the day of harvest, kale leaves from the control, KIO_3_-biofortified (KIO_3_), and 5,7-diiodo-8-quinolinol-biofortified (DIQ) groups were separated from the stems to obtain the edible portions. For each cultivar × iodine treatment combination, leaves collected from 18 plants were pooled and thoroughly mixed to obtain a composite sample. The pooled material was then divided among the raw and thermal-processing conditions. For each thermal-processing condition, three separate portions of the composite material were prepared and independently subjected to the respective processing treatment, providing three independent processing replicates per cultivar × iodine × processing combination. Thus, the three replicates represented independently processed portions derived from the same pooled plant material rather than independent biological replicates. Distilled water was used for all water-based treatments to minimize the introduction of exogenous iodine [19].

Steaming was performed in a combi-steam oven (Retigo Orange, Version 6 × GN1/1O 611, Rožnov, Czech Republic). Kale leaves were arranged in a single layer on stainless-steel trays and steamed at 100 °C for 15 min.

Blanching was performed in distilled water using an electric cooker (Mastercook KE 2003 B Dynamic, Wrocław, Poland). A 500 g portion of kale leaves was immersed in 1 L of distilled water maintained at 85–95 °C, corresponding to a sample-to-water ratio of 1:2 (*w*/*v*), and blanched for 3–5 min. After blanching, the processing water was discarded, and the leaves were immediately rinsed with cold distilled water.

Boiling was performed in distilled water using the same electric cooker. A 500 g portion of kale leaves was placed in 1 L of boiling distilled water in a stainless-steel pot, corresponding to a sample-to-water ratio of 1:2 (*w*/*v*), and boiled without a lid for 20 min. The boiling water was collected for separate iodine analysis. The initial and final water volumes were recorded to estimate water evaporation during processing; the volume decreased from 1 L to approximately 400 mL.

Following the respective processing treatments, all samples, including raw samples, were handled identically. The kale leaves were spread in a single layer on trays, frozen at −20 °C, and subsequently freeze-dried using a Christ Alpha Gamma 1–16 LSCplus freeze-dryer (Martin Christ Gefriertrocknungsanlagen GmbH, Osterode am Harz, Germany). The freeze-dried samples were ground using a WZ-1 laboratory mill (Zakład Badawczy Przemysłu Piekarskiego Sp. z o.o., Bydgoszcz, Poland) and stored in sealed polyethylene bags at 2–8 °C until extraction.

### 3.3. Preparation of Plant Extracts

Kale samples were finely ground and homogenized prior to extraction. An aliquot of 200 mg of each sample was extracted with 2.5 mL of 70% (*v*/*v*) aqueous methanol. The mixtures were subjected to ultrasonic-assisted extraction for 30 min, followed by centrifugation at 5000 rpm for 20 min. The resulting supernatants were carefully collected for further analysis. For each cultivar × iodine × processing combination, three independently processed portions derived from the same pooled plant material were analysed. Each processing replicate was extracted in triplicate, resulting in three technical extraction replicates per processing replicate. The technical extraction replicates were averaged prior to statistical analysis, yielding one analytical value for each of the three processing replicates. A pooled quality-control (QC) sample was prepared by combining equal volumes of all individual extracts.

### 3.4. LC–PDA–Q-Exactive Orbitrap HRMS Analysis

LC–PDA–HRMS analyses were carried out using an Ultimate 3000 ultra-high-performance liquid chromatography (UHPLC) system equipped with a photodiode array (PDA) detector (Thermo Scientific Dionex, Bremen, Germany) and hyphenated to a Q-Exactive™ Focus quadrupole–Orbitrap mass spectrometer (Thermo Scientific, Bremen, Germany) equipped with a heated electrospray ionization (HESI) source. Chromatographic separation was achieved on a Phenomenex Synergi Polar-RP column (250 mm × 4.6 mm, 4 μm, Phenomenex, Torrance, CA, USA) employing gradient elution with 0.1% (*v*/*v*) formic acid in deionized water (mobile phase A) and 0.1% (*v*/*v*) formic acid in acetonitrile (mobile phase B) at a constant flow rate of 0.6 mL/min. The gradient program started at 5% B, increased to 60% B at 10 min and to 80% B at 20 min, followed by a ramp to 100% B between 21 and 30 min. Prior to each injection, the system was re-equilibrated under initial conditions (5% B) for 7 min.

PDA spectra were recorded simultaneously during the LC–HRMS analyses, and chromatograms acquired at 325 nm were used for quantitative and semi-quantitative determination of selected phenolic compounds.

Mass spectrometric detection was performed in negative ionization mode. The HESI source was operated with the following settings: sheath gas flow rate of 35 arb, auxiliary gas flow rate of 15 arb, sweep gas flow rate of 3 arb, spray voltage of 2.5 kV, capillary temperature of 350 °C, S-lens RF level of 50, and heater temperature of 300 °C. Full-scan mass spectra were acquired over an *m*/*z* range of 120–1200 at a resolving power of 70,000 FWHM, with an AGC target of 2 × 10^5^ and a maximum injection time of 100 ms. Data-dependent MS^2^ experiments were conducted at a resolution of 17,500 FWHM using an isolation window of 3.0 *m*/*z*, normalized collision energy of 30 eV, an AGC target of 2 × 10^5^, and a maximum injection time of 100 ms. The injection volume was set to 8 μL.

Raw mass spectrometric data were processed using Compound Discoverer software (version 3.3.3.200, Thermo Scientific, San Jose, CA, USA). Samples were analysed in randomized order, with pooled quality control (QC) samples injected every nine study samples to monitor analytical stability throughout the analytical sequence. Seven blank injections were distributed at the beginning and throughout the analytical sequence to monitor background signals and potential carryover. Signal intensities were normalized using QC-based area correction implemented in Compound Discoverer, applying a minimum QC coverage threshold of 50% and a maximum QC relative standard deviation (RSD) of 30%. No missing values were present in the final processed dataset; therefore, no missing-value imputation was applied.

Compound annotation was based on accurate mass measurements of precursor ions, isotopic patterns, characteristic MS/MS fragmentation, diagnostic product ions and neutral losses, supported by database searches and comparison with published data. For compounds not sufficiently represented in spectral databases, MS/MS spectra were manually interpreted by assigning molecular formulas to product ions, evaluating sequential neutral losses, and reconstructing plausible fragmentation pathways. Identification confidence was assigned according to the framework proposed by Schymanski et al. (2014) [10]. Sinapic acid, neochlorogenic acid, and chlorogenic acid were assigned Level 1, as their identities were confirmed using authentic reference standards analysed under identical chromatographic and MS conditions, based on agreement in retention time, accurate mass, MS/MS fragmentation, and UV–Vis spectra. Compounds assigned Level 2b represented probable structures supported by diagnostic HRMS/MS evidence and, where available, corresponding literature data, whereas Level 3 was assigned when the available MS/MS evidence supported a tentative structural candidate but did not allow an unambiguous structural assignment. Detailed MS/MS data, proposed fragment assignments, identification confidence levels, and supporting literature references are provided in Supplementary Table S1.

### 3.5. Quantitative and Semi-Quantitative Determination of Selected Phenolic Compounds by HPLC-PDA

Selected major phenolic compounds were quantified or semi-quantified using PDA chromatograms recorded at 325 nm during the LC–PDA–HRMS analyses described in Section 3.4. Calibration curves were prepared using available reference standards. Caffeoylquinic acid I (compound **30**) and caffeoylquinic acid II (compound **34**) were quantified using the corresponding authentic standards, neochlorogenic acid and chlorogenic acid, respectively. Sinapic acid derivatives, including SinA2Hex III (17), SinAHex II (22), Sin-hFer2Hex (113), the co-eluting 2Sin2Hex III and Sin-Fer2Hex III (118 + 119), and 2Sin-Fer2Hex I (126), were semi-quantified using the sinapic acid calibration curve and expressed as sinapic acid equivalents. Kamp-hFer4Hex (70) was semi-quantified using the kaempferol calibration curve and expressed as kaempferol equivalents. Compounds **118** and **119** were not chromatographically resolved in the PDA trace and were therefore determined together as a single peak. Results were expressed as mg/g dry weight (DW).

Calibration curves were constructed using seven concentration levels, with three replicate measurements at each level (*n* = 21). Linear regression was performed using peak area as the response variable and standard concentration as the independent variable. The calibration ranges were 0.0100–0.2000 mg/mL for neochlorogenic acid, 0.0099–0.1980 mg/mL for chlorogenic acid, 0.00565–0.1130 mg/mL for sinapic acid, and 0.00623–0.1246 mg/mL for kaempferol. The corresponding coefficients of determination (R^2^) were 0.99505, 0.99649, 0.99612, and 0.99693, respectively. Limits of detection (LOD) and quantification (LOQ) were calculated as LOD = 3.3σ/S and LOQ = 10σ/S, where σ was the standard error of the regression intercept and S was the slope of the calibration curve. The corresponding LOD and LOQ values were 0.00601 and 0.01821 mg/mL for neochlorogenic acid, 0.00500 and 0.01515 mg/mL for chlorogenic acid, 0.00300 and 0.00910 mg/mL for sinapic acid, and 0.00294 and 0.00891 mg/mL for kaempferol, respectively. On a dry-weight basis, calculated using the extraction volume (2.5 mL) and sample mass (0.200 g), the corresponding LOD and LOQ values were 0.0751 and 0.2276, 0.0625 and 0.1894, 0.0375 and 0.1138, and 0.0368 and 0.1114 mg/g DW, respectively. Measurements below the LOQ were retained as back-calculated concentrations for calculation of group means and for the primary statistical analyses. The robustness of the statistical conclusions was additionally evaluated by sensitivity analyses in which measurements below the LOQ were replaced with either LOQ/2 or LOQ (Table S8).

### 3.6. Statistical Analysis

Statistical analyses were performed in R using RStudio (version 2025.09.2+418). For statistical analyses, the three independently processed portions derived from the same pooled plant material were treated as processing replicates. Technical extraction replicates were averaged prior to statistical analysis, yielding one analytical value per processing replicate. QC-corrected LC–HRMS signal intensities obtained as described above were used for subsequent statistical analyses. Unless stated otherwise, analyses were performed separately for green- and red-leaved kale.

To evaluate global patterns in phenolic composition across cultivars, thermal processing, and iodine treatments, principal component analysis (PCA) was applied to the LC–HRMS dataset comprising all annotated phenolic compounds. Peak intensities were log-transformed and subsequently mean-centered and scaled to unit variance prior to PCA. Heat map visualizations were generated separately for each cultivar using three processing replicates for each treatment combination, followed by row-wise autoscaling. For Figure 4, compounds were displayed in a fixed order according to their chemical classes without hierarchical clustering. For Supplementary Figure S1, hierarchical clustering was additionally applied to visualize groups of phenolic compounds showing similar treatment-associated response patterns.

To evaluate the relative contributions of thermal processing, iodine treatment, and their interaction to multivariate phenolic variation, variance partitioning based on the PERMANOVA model was performed separately for each cultivar using the adonis2 function of the vegan package. Euclidean distances were calculated from log-transformed and autoscaled LC–HRMS intensities. Because the three processing replicates within each cultivar × iodine × processing combination were derived from the same pooled plant material, they did not represent independent biological replicates of the iodine treatment. Therefore, the analysis was used to quantify the proportion of variation (R^2^) associated with processing, iodine treatment, and their interaction within the present dataset, rather than for inferential testing of iodine-related effects. Raw samples were excluded because they were available only for the iodine-free control and did not form part of the complete factorial design. The analysis was therefore performed on the 3 × 3 subset comprising three processing treatments (blanching, boiling, and steaming) and three iodine treatments (Control, KIO_3_, and DIQ), with three independently processed portions per treatment combination.

At the class level, phenolic compounds were grouped according to their chemical classification, and summed LC–HRMS signal intensities were calculated for each phenolic class. Summed signal intensities were transformed as log_10_(summed intensity + 1) prior to analysis. Because the three processing replicates within each cultivar × iodine treatment combination were independently processed portions derived from the same pooled plant material, statistical comparisons were restricted to the effect of thermal processing within each iodine treatment. Accordingly, for each cultivar, phenolic class, and iodine treatment (Control, KIO_3_, or DIQ), blanched, boiled, and steamed samples were compared separately using one-way ANOVA followed by Tukey-adjusted post-hoc comparisons (*p* < 0.05). No inferential comparisons were performed among iodine treatments. Raw samples were retained in graphical presentations as descriptive reference values but were not included in these analyses.

At the compound level, processing-related differences were evaluated separately for each cultivar using the processed samples. For each of the 135 annotated phenolic compounds, LC–HRMS signal intensities were log_10_-transformed prior to analysis. The steamed-versus-boiled contrast was estimated within each of the three pooled source materials corresponding to the Control, KIO_3_, and DIQ treatments and then averaged across these pools to obtain an overall processing contrast. Because each iodine treatment within a cultivar was represented by a single pooled biological source, iodine treatment was treated only as a source-pool stratification factor in this analysis and no inferential conclusions regarding iodine effects or Processing × Iodine interactions were drawn from these models. Contrast estimates were converted to log_2_ fold changes (log_2_FC), with positive values indicating higher relative LC–HRMS signal intensities in steamed samples and negative values indicating higher signals in boiled samples. *p*-values for the steamed-versus-boiled processing contrast were adjusted separately within each cultivar across the 135 compounds using the Benjamini–Hochberg false discovery rate (FDR) procedure. For visualization in volcano plots, compounds with BH-adjusted *p* < 0.05 and |log_2_FC| ≥ 0.5 were highlighted, with the fold-change threshold used as an effect-size criterion for visualization.

To descriptively examine whether iodine biofortification modified compound-level responses to processing, log_2_FC values for the steamed-versus-boiled contrast were calculated separately for iodine-free controls and samples biofortified with KIO_3_ or 5,7-diiodo-8-quinolinol (DIQ). For each compound and cultivar, the iodine-treatment-associated difference was expressed as Δlog_2_FC, calculated as the difference between the log_2_FC under a given iodine treatment and that of the corresponding iodine-free control. For each treatment combination, log_2_FC was calculated from the three independently processed portions as log_2_[(mean Steamed + 1)/(mean Boiled + 1)]. The compound-specific patterns associated with KIO_3_ and DIQ were further compared by calculating the difference between their respective Δlog_2_FC values (Δlog_2_FC_DIQ − Δlog_2_FC_KIO_3_). For visualization, the five compounds showing the largest absolute difference between the two iodine treatments were highlighted.

Concentration data obtained by PDA detection were analysed separately for each cultivar and compound. Because the three processing replicates within each cultivar × iodine treatment combination were independently processed portions derived from the same pooled plant material, statistical comparisons were restricted to the effect of thermal processing within each iodine treatment. Accordingly, for each cultivar, compound, and iodine treatment (Control, KIO_3_, or DIQ), blanched, boiled, and steamed samples were compared separately using one-way ANOVA followed by Tukey-adjusted post-hoc comparisons (*p* < 0.05). No inferential comparisons were performed among iodine treatments. Raw samples were retained in graphical presentations as descriptive reference values but were not included in these analyses.

## 4. Conclusions

The present study demonstrates that the phenolic composition of iodine-biofortified kale is strongly shaped by thermal processing and differs between the two investigated cultivars. Across the complete dataset, cultivar was the major source of variation, whereas within each cultivar thermal processing accounted for the largest proportion of multivariate variation among processed samples. Steaming and boiling produced particularly distinct phenolic profiles, with steaming generally associated with higher relative LC–HRMS signals for numerous phenolic compounds, especially in red-leaved kale. Differences were also observed among the Control, KIO_3_, and DIQ sample pools, and the magnitude and direction of these differences varied across processing conditions. However, because each cultivar × iodine treatment combination was represented by a single pooled biological source, these iodine-associated patterns and the Processing × Iodine component of multivariate variation should be interpreted descriptively rather than as inferential evidence of iodine treatment effects or their interaction with processing.

Because iodine-treated raw samples were not available, comparisons involving the raw material were limited to untreated controls, and analyses of the processed samples were restricted accordingly. Moreover, the LC–HRMS data represent relative analytical responses and do not establish absolute phenolic retention during processing, particularly because processing water was not analysed for phenolic compounds. PDA measurements provided concentrations for compounds quantified using authentic standards and semi-quantitative estimates expressed as standard equivalents for the remaining selected phenolics. Despite these limitations, the study provides a compound-resolved characterization of processing-related changes in the phenolic profiles of iodine-biofortified kale and highlights the importance of considering culinary processing when characterizing the phytochemical composition of biofortified vegetables.

## 5. Patents

The method of biofortification of vegetables in iodine cultivated using a traditional, soilless, and hydroponic method, and the use of 5,7-diiodo-8-quinolinol for biofortification of vegetables with iodine. These are covered under patent application number P.443221 for the compounds (Polish Patent Office; 21 December 2022).

## Figures and Tables

**Table 1:** Phenolic compounds annotated in kale leaves grouped according to phenolic classes, including compound numbers, names, abbreviations, molecular formulas, and deprotonated molecular ions ([M − H]^−^).

No	Compound Name	Abbreviation	Molecular Formula	[M − H]^−^ (*m/z*)
Phenolic acids and their glycosides (**1**–**26**)				
1	Syringoylquinic acid	SyrQA	C_16_H_20_O_10_	371.0980
2	Caffeic acid dihexoside	CafA2Hex	C_21_H_28_O_14_	503.1410
3	Protocatechuic acid hexoside	PrcAHex	C_13_H_16_O_9_	315.0720
4	*p*-Coumaric acid dihexoside (I)	CoumA2Hex I	C_21_H_28_O_13_	487.1460
5	*p*-Coumaric acid dihexoside (II)	CoumA2Hex II	C_21_H_28_O_13_	487.1461
6	Gallic acid hexoside	GAHex	C_13_H_16_O_10_	331.0670
7	Sinapic acid dihexoside (I)	SinA2Hex I	C_23_H_32_O_15_	547.1672
8	*p*-Coumaric acid dihexoside (III)	CoumA2Hex III	C_21_H_28_O_13_	487.1461
9	Ferulic acid dihexoside (I)	FerA2Hex I	C_22_H_30_O_14_	517.1567
10	Sinapic acid dihexoside (II)	SinA2Hex II	C_23_H_32_O_15_	547.1672
11	Caffeic acid hexoside (I)	CafAHex I	C_15_H_18_O_9_	341.0879
12	Hydroxyferulic acid hexoside (I)	hFerAHex I	C_16_H_20_O_10_	371.0983
13	Sinapic acid dipentoside	SinA2Pent	C_21_H_28_O_13_	487.1459
14	Caffeic acid hexoside (II)	CafAHex II	C_15_H_18_O_9_	341.0879
15	Hydroxyferulic acid hexoside (II)	hFerAHex II	C_16_H_20_O_10_	371.0986
16	Ferulic acid dihexoside (II)	FerA2Hex II	C_22_H_30_O_14_	517.1565
17	Sinapic acid dihexoside (III)	SinA2Hex III	C_23_H_32_O_15_	547.1672
18	Ferulic acid hexoside (I)	FerAHex I	C_16_H_20_O_9_	355.1028
19	Sinapic acid dihexoside (IV)	SinA2Hex IV	C_23_H_32_O_15_	547.1672
20	*p*-Coumaric acid hexoside	CoumAHex	C_15_H_18_O_8_	325.0921
21	Sinapic acid hexoside (I)	SinAHex I	C_17_H_22_O_10_	385.1142
22	Sinapic acid hexoside (II)	SinAHex II	C_17_H_22_O_10_	385.1135
23	Ferulic acid hexoside (II)	FerAHex II	C_16_H_20_O_9_	355.1035
24	Caffeic acid	CafA	C_9_H_8_O_4_	179.0342
25	Hydroxyferulic acid	hFerA	C_10_H_10_O_5_	209.0451
26	Sinapic acid	SinA	C_11_H_12_O_5_	223.0608
Hydroxycinnamoylquinic acids (chlorogenic acid derivatives) (**27**–**57**)				
27	Caffeoylquinic acid hexoside (I)	CafQAHex I	C_22_H_28_O_14_	515.1408
28	*p*-Coumaroylquinic acid hexoside	CoumQAHex	C_22_H_28_O_13_	499.1461
29	Caffeoylquinic acid hexoside (II)	CafQAHex II	C_22_H_28_O_14_	515.1408
30	Caffeoylquinic acid (I)	CafQA I	C_16_H_18_O_9_	353.0879
31	Feruloylquinic acid hexoside	FerQAHex	C_23_H_30_O_14_	529.1563
32	Sinapoyl-caffeoylquinic acid pentoside hexoside	Sin-CafQAPentHex	C_38_H_48_O_23_	871.2521
33	*p*-Coumaroylquinic acid (I)	CoumQA I	C_16_H_18_O_8_	337.0923
34	Caffeoylquinic acid (II)	CafQA II	C_16_H_18_O_9_	353.0879
35	Sinapoyl-caffeoylquinic acid hexoside (I)	Sin-CafQAHex I	C_33_H_40_O_19_	739.2100
36	Feruloylquinic acid (I)	FerQA I	C_17_H_20_O_9_	367.1036
37	Caffeoylquinic acid (III)	CafQA III	C_16_H_18_O_9_	353.0871
38	Hydroxyferuloyl-caffeoylquinic acid hexoside	hFer-CafQAHex	C_32_H_38_O_19_	725.1942
39	*p*-Coumaroylquinic acid (II)	CoumQA II	C_16_H_18_O_8_	337.0929
40	Disinapoyl-coumaroylquinic acid dihexoside	2Sin-CoumQA2Hex	C_50_H_60_O_27_	1091.3252
41	Sinapoyl-dicoumaroylquinic acid hexoside	Sin-2CoumQAHex	C_49_H_58_O_26_	1061.3149
42	Feruloylquinic acid (II)	FerQA II	C_17_H_20_O_9_	367.1036
43	*p*-Coumaroylquinic acid (III)	CoumQA III	C_16_H_18_O_8_	337.0925
44	Disinapoyl-caffeoylquinic acid dihexoside	2Sin-CafQA2Hex	C_50_H_60_O_28_	1107.3196
45	Feruloylquinic acid (III)	FerQA III	C_17_H_20_O_9_	367.1035
46	Syringaldehyde-sinapoylquinic acid hexoside	SyringAld-SinQAHex	C_33_H_40_O_18_	723.2147
47	Feruloyl-caffeoylquinic acid hexoside (I)	Fer-CafQAHex I	C_32_H_38_O_18_	709.1991
48	Sinapoyl-caffeoylquinic acid hexoside (II)	Sin-CafQAHex II	C_33_H_40_O_19_	739.2099
49	Sinapoyl-hydroxyferuloyl-caffeoylquinic acid hexoside	Sin-hFer-CafQAHex	C_43_H_48_O_23_	931.2520
50	Feruloyl-caffeoylquinic acid hexoside (II)	Fer-CafQAHex II	C_32_H_38_O_18_	709.1992
51	Disinapoyl-caffeoylquinic acid hexoside (I)	2Sin-CafQAHex I	C_44_H_50_O_23_	945.2674
52	Dihydroferuloyl-disinapoyl-coumaroylquinic acid hexoside	dhFer-2Sin-CoumQAHex	C_54_H_62_O_26_	1125.3459
53	Feruloyl-sinapoyl-caffeoylquinic acid hexoside (I)	Fer-Sin-CafQAHex I	C_43_H_48_O_22_	915.2570
54	Feruloyl-caffeoylquinic acid	Fer-CafQA	C_26_H_28_O_13_	547.1450
55	Disinapoyl-caffeoylquinic acid hexoside (II)	2Sin-CafQAHex II	C_44_H_50_O_23_	945.2673
56	Feruloyl-sinapoyl-caffeoylquinic acid hexoside (II)	Fer-Sin-CafQAHex II	C_43_H_48_O_22_	915.2569
57	Disinapoyl-caffeoylquinic acid hexoside (III)	2Sin-CafQAHex III	C_44_H_50_O_23_	945.2674
Flavonols (**58**–**103**)				
58	Quercetin tetrahexoside	Quer4Hex	C_39_H_50_O_27_	949.2471
59	Quercetin trihexoside	Quer3Hex	C_33_H_40_O_22_	787.1943
60	Quercetin rhamnoside trihexoside	QuerRham3Hex	C_39_H_50_O_26_	933.2519
61	Kaempferol trihexoside (I)	Kamp3Hex I	C_33_H_40_O_21_	771.1996
62	Isorhamnetin trihexoside	Irham3Hex	C_34_H_42_O_22_	801.2099
63	Kaempferol-hydroxyferuloyl pentahexoside	Kamp-hFer5Hex	C_55_H_68_O_35_	643.1709 *
64	Quercetin-hydroxyferuloyl tetrahexoside	Quer-hFer4Hex	C_49_H_58_O_31_	1141.2890
65	Quercetin-caffeoyl tetrahexoside	Quer-Caf4Hex	C_48_H_56_O_30_	1111.2784
66	Kaempferol-caffeoyl pentahexoside	Kamp-Caf5Hex	C_54_H_66_O_34_	628.1651 *
67	Quercetin-sinapoyl pentahexoside	Quer-Sin5Hex	C_56_H_70_O_36_	658.1758 *
68	Eriodictyol dihexoside	Erio2Hex	C_27_H_32_O_16_	611.1620
69	Kaempferol tetrahexoside	Kamp4Hex	C_39_H_50_O_26_	933.2519
70	Kaempferol-hydroxyferuloyl tetrahexoside	Kamp-hFer4Hex	C_49_H_58_O_30_	562.1477 *
71	Quercetin-hydroxyferuloyl trihexoside	Quer-hFer3Hex	C_43_H_48_O_26_	979.2349
72	Kaempferol-sinapoyl pentahexoside	Kamp-Sin5Hex	C_56_H_70_O_35_	650.1782*
73	Kaempferol-caffeoyl tetrahexoside	Kamp-Caf4Hex	C_48_H_56_O_29_	1095.2835
74	Quercetin-sinapoyl tetrahexoside	Quer-Sin4Hex	C_50_H_60_O_31_	577.1492 *
75	Kaempferol-feruloyl pentahexoside	Kamp-Fer5Hex	C_55_H_68_O_34_	635.1729 *
76	Kaempferol trihexoside (II)	Kamp3Hex II	C_33_H_40_O_21_	771.1994
77	Kaempferol-hydroxyferuloyl trihexoside	Kamp-hFer3Hex	C_43_H_48_O_25_	963.2413
78	Kaempferol-sinapoyl tetrahexoside	Kamp-Sin4Hex	C_50_H_60_O_30_	1139.3094
79	Quercetin-sinapoyl trihexoside	Quer-Sin3Hex	C_44_H_50_O_26_	993.2519
80	Kaempferol-caffeoyl trihexoside	Kamp-Caf3Hex	C_42_H_46_O_24_	933.2309
81	Kaempferol-feruloyl tetrahexoside	Kamp-Fer4Hex	C_49_H_58_O_29_	1109.2991
82	Kaempferol-sinapoyl trihexoside	Kamp-Sin3Hex	C_44_H_50_O_25_	977.2571
83	Kaempferol-feruloyl trihexoside	Kamp-Fer3Hex	C_43_H_48_O_24_	947.2466
84	Kaempferol-dihydroxyferuloyl pentahexoside	Kamp-2hFer5Hex	C_65_H_76_O_39_	739.1915 *
85	Kaempferol-hydroxyferuloyl-sinapoyl pentahexoside	Kamp-hFer-Sin5Hex	C_66_H_78_O_39_	746.1994 *
86	Quercetin-disinapoyl pentahexoside	Quer-2Sin5Hex	C_67_H_80_O_40_	761.2046 *
87	Kaempferol-sinapoyl trihexoside (hydrated)	Kamp-Sin3Hex-h	C_44_H_52_O_26_	995.2677
88	Kaempferol-feruloyl trihexoside (hydrated)	Kamp-Fer3Hex-h	C_43_H_50_O_25_	965.2573
89	Kaempferol-feruloyl-hydroxyferuloyl pentahexoside	Kamp-Fer-hFer5Hex	C_65_H_76_O_38_	731.1942 *
90	Kaempferol dihexoside (I)	Kamp2Hex I	C_27_H_30_O_16_	609.1464
91	Kaempferol-disinapoyl pentahexoside	Kamp-2Sin5Hex	C_67_H_80_O_39_	753.2065 *
92	Quercetin dihexoside	Quer2Hex	C_27_H_30_O_17_	625.1414
93	Kaempferol-sinapoyl-hydroxyferuloyl tetrahexoside	Kamp-Sin-hFer4Hex	C_60_H_68_O_34_	665.1730 *
94	Quercetin-disinapoyl tetrahexoside	Quer-2Sin4Hex	C_61_H_70_O_35_	680.1781 *
95	Kaempferol-sinapoyl-feruloyl pentahexoside	Kamp-Sin-Fer5Hex	C_66_H_78_O_38_	738.2019 *
96	Kaempferol-diferuloyl pentahexoside	Kamp-2Fer5Hex	C_65_H_76_O_37_	723.1965 *
97	Kaempferol-disinapoyl tetrahexoside	Kamp-2Sin4Hex	C_61_H_70_O_34_	672.1812 *
98	Kaempferol dihexoside (II)	Kamp2Hex II	C_27_H_30_O_16_	609.1465
99	Kaempferol-hydroxyferuloyl dihexoside	Kamp-hFer2Hex	C_37_H_38_O_20_	801.1890
100	Kaempferol-sinapoyl dihexoside	Kamp-Sin2Hex	C_38_H_40_O_20_	815.2045
101	Kaempferol-sinapoyl-feruloyl trihexoside	Kamp-Sin-Fer3Hex	C_54_H_60_O_29_	1171.3148
102	Quercetin glucuronide	QuerGluc	C_21_H_18_O_13_	477.0674
103	Kaempferol-feruloyl dihexoside	Kamp-Fer2Hex	C_37_H_38_O_19_	785.1939
Anthocyanins (**104**–**105**)				
104	Cyanidin disinapoyl trihexoside	Cy-2Sin3Hex	C_55_H_61_O_29_^+^	1183.3150
105	Cyanidin feruloyl-sinapoyl trihexoside	Cy-Fer-Sin3Hex	C_54_H_59_O_28_^+^	1153.30445
Lignans (**106**–**107**)				
106	Lariciresinol dihexoside	Lar2Hex	C_32_H_44_O_16_	683.2560
107	Pinoresinol dihexoside	Pin2Hex	C_32_H_42_O_16_	681.2408
Complex hydroxycinnamic acid derivatives (**108**–**135**)				
108	*p*-Coumaroyl-sinapoyl trihexoside (I)	Coum-Sin3Hex I	C_38_H_48_O_22_	855.2568
109	Feruloyl-sinapoyl trihexoside	Fer-Sin3Hex	C_39_H_50_O_23_	885.2675
110	*p*-Coumaroyl-sinapoyl trihexoside (II)	Coum-Sin3Hex II	C_38_H_48_O_22_	855.2571
111	Disinapoyl dihexoside (I)	2Sin2Hex I	C_34_H_42_O_19_	753.2253
112	Sinapoyl-feruloyl dihexoside (I)	Sin-Fer2Hex I	C_33_H_40_O_18_	723.2150
113	Sinapoyl-hydroxyferuloyl dihexoside	Sin-hFer2Hex	C_33_H_40_O_19_	739.2096
114	Sinapoyl-feruloyl-dihydroferuloyl dihexoside	Sin-Fer-dhFer2Hex	C_43_H_52_O_22_	919.2881
115	Disinapoyl dihexoside (II)	2Sin2Hex II	C_34_H_42_O_19_	753.2254
116	Diferuloyl-dihydroferuloyl dihexoside	2Fer-dhFer2Hex	C_42_H_50_O_21_	889.2776
117	Sinapoyl-feruloyl dihexoside (II)	Sin-Fer2Hex II	C_33_H_40_O_18_	723.2150
118	Disinapoyl dihexoside (III)	2Sin2Hex III	C_34_H_42_O_19_	753.2252
119	Sinapoyl-feruloyl dihexoside (III)	Sin-Fer2Hex III	C_33_H_40_O_18_	723.2147
120	Diferuloyl dihexoside (I)	2Fer2Hex I	C_32_H_38_O_17_	693.2041
121	Disinapoyl dihexoside (IV)	2Sin2Hex IV	C_34_H_42_O_19_	753.2252
122	Sinapoyl-feruloyl dihexoside (IV)	Sin-Fer2Hex IV	C_33_H_40_O_18_	723.2148
123	Diferuloyl dihexoside (II)	2Fer2Hex II	C_32_H_38_O_17_	693.2042
124	Sinapoyl-sinapaldehyde dihexoside	Sin-SinAld2Hex	C_34_H_42_O_18_	737.2305
125	Trisinapoyl dihexoside (I)	3Sin2Hex I	C_45_H_52_O_23_	959.2830
126	Disinapoyl-feruloyl dihexoside (I)	2Sin-Fer2Hex I	C_44_H_50_O_22_	929.2727
127	Sinapoyl-diferuloyl dihexoside (I)	Sin-2Fer2Hex I	C_43_H_48_O_21_	899.2622
128	Disinapoyl hexoside	2SinHex	C_28_H_32_O_14_	591.1725
129	Sinapoyl-feruloyl hexoside	Sin-FerHex	C_27_H_30_O_13_	561.1617
130	Trisinapoyl dihexoside (II)	3Sin2Hex II	C_45_H_52_O_23_	959.2832
131	Disinapoyl-feruloyl dihexoside (II)	2Sin-Fer2Hex II	C_44_H_50_O_22_	929.2728
132	Diferuloyl hexoside	2FerHex	C_26_H_28_O_12_	531.1510
133	Sinapoyl-diferuloyl dihexoside (II)	Sin-2Fer2Hex II	C_43_H_48_O_21_	899.2622
134	Trisinapoyl dihexoside (III)	3Sin2Hex III	C_45_H_52_O_23_	959.2831
135	Disinapoyl-feruloyl dihexoside (III)	2Sin-Fer2Hex III	C_44_H_50_O_22_	929.2727

* For compounds marked with an asterisk, the reported *m*/*z* values correspond to doubly charged deprotonated ions ([M − 2H]^2−^). Detailed MS/MS data and annotation confidence levels are provided in Supplementary Table S1.

## Data Availability

The data presented in this study are available within the article and Supplementary Materials. Additional data are available from the corresponding author upon reasonable request.

## Supplementary Materials

The following supporting information can be downloaded at: https://www.mdpi.com/article/10.3390/molecules31203660/s1, File S1. Data associated with this article can be found, in the online version, at the publisher’s website.
