# Supplementary material for: Thermal Processing Shapes Phenolic Profiles in Iodine-Biofortified Kale: Cultivar- and Iodine-Treatment-Associated Patterns Revealed by LC–HRMS
Source: Molecules. 2026 Oct 13;31(20):3660. doi: 10.3390/molecules31203660 (PMC13707524; doi:10.3390/molecules31203660)
Supplement: Supplementary file 1 [file molecules-31-03660-s001.zip › molecules-4581710-supplementary.pdf]

Supplementary

Thermal Processing Shapes Phenolic Profiles in Iodine-Biofortified Kale: Cultivar- and Iodine-Treatment-Associated Patterns Revealed by LC–HRMS

Barbara Kusznierewicz[a*], Justyna Waśniowska[b*] and Aneta Koronowicz[b]

Table S1. Comprehensive list of phenolic compounds annotated in kale leaves, including molecular formulas, retention times, deprotonated molecular ions ([M−H]$^-$), characteristic MS/MS fragment ions and neutral losses used for compound annotation, identification confidence levels, and supporting literature references.

No	Rt (min)	Name/abbreviation/formula/molecular weight	[M − H]$^-$ Δm [ppm]	Product ions<br>Neutral-loss	 *m/z* (formula)	Identification confidence level/ reference(s)
1	9.103	Syringoylquinic acid (SyrQA)<br>$C_{16}H_{20}O_{10}$<br>Mw=372.1056	371.0985<br>-1.8 ppm	[M − H − Quinoyl − CO_2 − H_2O]$^-$<br>[M − H − Syringoyl]$^-$<br>[M − H − Syringoyl − H_2O]$^-$<br>[M − H − Quinoyl − H_2O]$^-$	135.044 ($C_8H_7O_2$)<br>191.055 ($C_7H_{11}O_6$)<br>173.045 ($C_7H_9O_5$)<br>179.034 ($C_9H_7O_4$)	2b
2	9.475	Caffeic acid dihexoside (CafA2Hex)<br>$C_{21}H_{28}O_{14}$<br>Mw=504.1479	503.1410<br>-1.8 ppm	[M − H − 2Hex − H_2O]$^-$<br>[M − H − 2Hex]$^-$<br>[M − H − Hex]$^-$	161.023 ($C_9H_5O_3$)<br>179.034 ($C_9H_7O_4$)<br>341.088 ($C_{15}H_{17}O_9$)	2b
3	9.605	Protocatechuic acid hexoside (PrcAHex)<br>$C_{13}H_{16}O_9$<br>Mw=316.0794	315.0720<br>-1.2 ppm	[M − H − Hex − CO_2]$^-$<br>[M − H − Hex]$^-$	109.028 ($C_6H_5O_2$)<br>153.018 ($C_7H_5O_4$)	2b
4	9.648	*p*-Coumaric acid dihexoside (I) (CoumA2Hex I)<br>$C_{21}H_{28}O_{13}$<br>Mw=488.1529	487.1460<br>-1.7 ppm	[M − H − 2Hex]$^-$<br>[M − H − Hex − $C_4H_8O_4$]$^-$<br>[M − H − Hex − $C_3H_8O_3$]$^-$<br>[M − H − Hex − $C_5H_{10}O_4$]$^-$<br>[M − H − Hex]$^-$	163.039 ($C_9H_7O_3$)<br>205.050 ($C_{11}H_9O_4$)<br>233.045 ($C_{12}H_9O_5$)<br>191.034 ($C_{10}H_7O_4$)<br>325.093 ($C_{15}H_{17}O_8$)	2b
5	9.778	*p*-Coumaric acid dihexoside (II) (CoumA2Hex II)<br>$C_{21}H_{28}O_{13}$<br>Mw=488.1529	487.1461<br>-1.9 ppm	[M − H − 2Hex]$^-$<br>[M − H − Hex − $C_5H_{10}O_4$]$^-$<br>[M − H − Hex − $C_3H_4O_4$]$^-$<br>[M − H − Hex − $C_5H_8O_3$]$^-$<br>[M − H − Hex − $C_4H_8O_4$]$^-$<br>[M − H − Hex]$^-$	163.039 ($C_9H_7O_3$)<br>191.034 ($C_{10}H_7O_4$)<br>221.081 ($C_{12}H_{13}O_4$)<br>209.045 ($C_{10}H_9O_5$)<br>205.050 ($C_{11}H_9O_4$)<br>325.093 ($C_{15}H_{17}O_8$)	2b
6	9.781	Gallic acid hexoside (GAHex)<br>$C_{13}H_{16}O_{10}$<br>Mw=332.0743	331.0670<br>-1.4 ppm	[M − H − Hex]$^-$<br>[M − H − Hex − CO_2]$^-$	169.013 ($C_7H_5O_5$)<br>125.023 ($C_6H_5O_3$)	2b
7	9.788	Sinapic acid dihexoside (I) (SinA2Hex I)<br>$C_{23}H_{32}O_{15}$<br>Mw=548.1741	547.1672<br>-1.8 ppm	[M − H − 2Hex]$^-$<br>[M − H − 2Hex − H_2O]$^-$<br>[M − H − Hex]$^-$<br>[M − H − Hex − $C_4H_8O_4$]$^-$	223.061 ($C_{11}H_{11}O_5$)<br>205.050 ($C_{11}H_9O_4$)<br>385.114 ($C_{17}H_{21}O_{10}$)<br>265.072 ($C_{13}H_{13}O_6$)	2b
8	9.914	*p*-Coumaric acid dihexoside (III) (CoumA2Hex III)<br>$C_{21}H_{28}O_{13}$<br>Mw=488.1529	487.1461<br>-1.8 ppm	[M − H − Hex − $C_5H_8O_3$]$^-$<br>[M − H − Hex − $C_5H_{10}O_4$]$^-$<br>[M − H − 2Hex]$^-$<br>[M − H − $C_5H_8O_3$]$^-$<br>[M − H − $C_5H_{10}O_4$]$^-$	209.045 ($C_{10}H_9O_5$)<br>191.034 ($C_{10}H_7O_4$)<br>163.039 ($C_9H_7O_3$)<br>371.099 ($C_{16}H_{19}O_{10}$)<br>353.088 ($C_{16}H_{17}O_9$)	2b
9	9.931	Ferulic acid dihexoside (I) (FerA2Hex I)<br>$C_{22}H_{30}O_{14}$<br>Mw=518.1636	517.1567<br>-1.9 ppm	[M − H − 2Hex]$^-$<br>[M − H − Hex]$^-$<br>[M − H − 2Hex − H_2O]$^-$	193.049 ($C_{10}H_9O_4$)<br>355.103 ($C_{16}H_{19}O_9$)<br>175.039 ($C_{10}H_7O_3$)	2b
10	10.207	Sinapic acid dihexoside (II) (SinA2Hex II)<br>$C_{23}H_{32}O_{15}$<br>Mw=548.1741	547.1672<br>-1.7 ppm	[M − H − 2Hex]$^-$<br>[M − H − 2Hex − H_2O]$^-$<br>[M − H − $C_4H_8O_4$]$^-$<br>[M − H − Hex − $C_4H_8O_4$]$^-$<br>[M − H − Hex − $C_4H_8O_4$ − H_2O]$^-$	223.061 ($C_{11}H_{11}O_5$)<br>205.050 ($C_{11}H_9O_4$)<br>427.125 ($C_{19}H_{23}O_{11}$)<br>265.072 ($C_{13}H_{13}O_6$)<br>247.061 ($C_{13}H_{11}O_5$)	2b
11	10.346	Caffeic acid hexoside (I) (CafAHex I)<br>$C_{15}H_{18}O_9$<br>Mw=342.0951	341.0879<br>-1.9 ppm	[M − H − Hex − CO_2]$^-$<br>[M − H − Hex − H_2O]$^-$<br>[M − H − Hex]$^-$	135.044 ($C_8H_7O_2$)<br>161.023 ($C_9H_5O_3$)<br>179.034 ($C_9H_7O_4$)	2b
12	10.382	Hydroxyferulic acid hexoside (I) (hFerAHex I)<br>$C_{16}H_{20}O_{10}$<br>Mw=372.1056	371.0983<br>-1.4 ppm	[M − H − Hex − H_2O]$^-$<br>[M − H − Hex]$^-$	191.034 ($C_{10}H_7O_4$)<br>209.045 ($C_{10}H_9O_5$)	2b
13	10.541	Sinapic acid dipentoside (SinA2Pent)<br>$C_{21}H_{28}O_{13}$<br>Mw=488.1529	487.1459<br>-1.6 ppm	[M − H − 2Pent]$^-$<br>[M − H − 2Pent − H_2O]$^-$	223.061 ($C_{11}H_{11}O_5$)<br>205.050 ($C_{11}H_9O_4$)	2b
14	10.545	Caffeic acid hexoside (II) (CafAHex II)<br>$C_{15}H_{18}O_9$<br>Mw=342.0951	341.0879<br>-1.9 ppm	[M − H − Hex − H_2O]$^-$<br>[M − H − Hex − CO_2]$^-$<br>[M − H − Hex]$^-$	161.023 ($C_9H_5O_3$)<br>135.044 ($C_8H_7O_2$)<br>179.034 ($C_9H_7O_4$)	2b
15	10.58	Hydroxyferulic acid hexoside (II) (hFerAHex II)<br>$C_{16}H_{20}O_{10}$<br>Mw=372.1056	371.0986<br>-1.8 ppm	[M − H − Hex − H_2O]$^-$<br>[M − H − Hex − CO − H_2O]$^-$	191.034 ($C_{10}H_7O_4$)<br>163.039 ($C_9H_7O_3$)	2b
16	10.603	Ferulic acid dihexoside (II) (FerA2Hex II)<br>$C_{22}H_{30}O_{14}$<br>Mw=518.1635	517.1565<br>-1.5 ppm	[M − H − 2Hex]$^-$<br>[M − H − 2Hex − H_2O]$^-$	193.049 ($C_{10}H_9O_4$)<br>175.039 ($C_{10}H_7O_3$)	2b
17	10.608	Sinapic acid dihexoside (III) (SinA2Hex III)<br>$C_{23}H_{32}O_{15}$	547.1672<br>-1.7 ppm	[M − H − 2Hex]$^-$<br>[M − H − 2Hex − H_2O]$^-$	223.061 ($C_{11}H_{11}O_5$)<br>205.050 ($C_{11}H_9O_4$)	2b

		Mw=548.1741				
**18**	10.697	Ferulic acid hexoside (I) (FerAHex I)<br>$C_{16}H_{20}O_9$<br>Mw=356.1107	355.1028<br>0.4 ppm	$[M - H - Hex - CO_2 - CH_3]^-$<br>$[M - H - Hex - CO_2]^-$<br>$[M - H - Hex - CH_3]^-$<br>$[M - H - Hex]^-$	134.036 ($C_8H_6O_2$)<br>149.059 ($C_9H_9O_2$)<br>178.026 ($C_9H_6O_4$)<br>193.049 ($C_{10}H_9O_4$)	2b
**19**	10.744	Sinapic acid dihexoside (IV) (SinA2Hex IV)<br>$C_{23}H_{32}O_{15}$<br>Mw=548.1741	547.1672<br>-1.7 ppm	$[M - H - 2Hex]^-$<br>$[M - H - 2Hex - H_2O]^-$	223.061 ($C_{11}H_{11}O_5$)<br>205.050 ($C_{11}H_9O_4$)	2b
**20**	10.767	*p*-Coumaric acid hexoside (CoumAHex)<br>$C_{15}H_{18}O_8$<br>Mw=326.1002	325.0921<br>0.8 ppm	$[M - H - Hex - CO_2]^-$<br>$[M - H - Hex]^-$	119.04897 (C_8H_7O)<br>163.03908 ($C_9H_7O_3$)	2b
**21**	10.979	Sinapic acid hexoside (I) (SinAHex I)<br>$C_{17}H_{22}O_{10}$<br>Mw=386.1213	385.1142<br>-1.9 ppm	$[M - H - Hex - CH_3]^-$<br>$[M - H - Hex - CO_2 - CH_3]^-$<br>$[M - H - Hex]^-$	208.037 ($C_{10}H_8O_5$)<br>164.047 ($C_9H_8O_3$)<br>223.061 ($C_{11}H_{11}O_5$)	2b
**22**	11.271	Sinapic acid hexoside (II) (SinAHex II)<br>$C_{17}H_{22}O_{10}$<br>Mw=386.1213	385.1135<br>-0.1 ppm	$[M - H - Hex - H_2O)]^-$<br>$[M - H - Hex - H_2O - CH_3)]^-$<br>$[M - H - Hex - CO_2 - CH_3)]^-$<br>$[M - H - Hex]^-$	205.050 ($C_{11}H_9O_4$)<br>190.026 ($C_{10}H_6O_4$)<br>164.047 ($C_9H_8O_3$)<br>223.060 ($C_{11}H_{11}O_5$)	2b
**23**	11.295	Ferulic acid hexoside (II) (FerAHex II)<br>$C_{16}H_{20}O_9$<br>Mw=356.1107	355.1035<br>-1.8 ppm	$[M - H - Hex - H_2O]^-$<br>$[M - H - Hex - H_2O - CH_3]^-$<br>$[M - H - Hex - CO_2 - CH_3]^-$	175.039 ($C_{10}H_7O_3$)<br>160.015 ($C_9H_4O_3$)<br>134.036 ($C_8H_6O_2$)	2b
**24**	12.135	Caffeic acid (CafA)<br>$C_9H_8O_4$<br>Mw=180.0423	179.0342<br>1.2 ppm	$[M - H - CO_2]^-$<br>$[M - H - CO_2 - CO]^-$<br>$[M - H - CO_2 - H_2O]^-$	135.044 ($C_8H_7O_2$)<br>107.049 (C_7H_7O)<br>117.033 (C_8H_5O)	2b<br>Lin et al., 2009
**25**	12.198	Hydroxyferulic acid (hFerA)<br>$C_{10}H_{10}O_5$<br>Mw=210.0528	209.0451<br>-0.8 ppm	$[M - H - CO_2 - CH_3]^-$<br>$[M - H - CO_2 - CO - CH_3]^-$	150.031 ($C_8H_6O_3$)<br>122.036 ($C_7H_6O_2$)	2b
**26**	13.29	Sinapic acid (SinA)<br>$C_{11}H_{12}O_5$<br>Mw=224.0685	223.0608<br>-1.0 ppm	$[M - H - 2CO - 2CH_2 - H_2O]^-$<br>$[M - H - CO - 2CH_2 - H_2O]^-$<br>$[M - H - 2CH_3]^-$<br>$[M - H - CO - 2CH_3]^-$	121.028 ($C_7H_5O_2$)<br>149.023 ($C_8H_5O_3$)<br>193.013 ($C_9H_5O_5$)<br>165.018 ($C_8H_5O_4$)	1<br>Lin and Harnly, 2009
		**Hydroxycinnamoylquinic acids (chlorogenic acid derivatives)**				
**27**	9.504	Caffeoylquinic acid hexoside (I) (CafQAHex I)<br>$C_{22}H_{28}O_{14}$<br>Mw=516.1479	515.1408<br>-1.5 ppm	$[M - H - Hex - Quinoyl]^-$<br>$[M - H - Hex - Caffeoyl - H_2O]^-$<br>$[M - H - Hex - Caffeoyl]^-$	179.034 ($C_9H_7O_4$)<br>173.045 ($C_7H_9O_5$)<br>191.055 ($C_7H_{11}O_6$)	2b
**28**	9.546	*p*-Coumaroylquinic acid hexoside (CoumQAHex)<br>$C_{22}H_{28}O_{13}$<br>Mw=500.1529	499.1461<br>-1.9 ppm	$[M - H - Hex - Quinoyl]^-$<br>$[M - H - Hex - Quinoyl - CO_2]^-$<br>$[M - H - Hex - Coumaroyl - H_2O]^-$<br>$[M - H - Quinoyl]^-$	163.039 ($C_9H_7O_3$)<br>119.049 (C_8H_7O)<br>173.045 ($C_7H_9O_5$)<br>325.093 ($C_{15}H_{17}O_8$)	2b
**29**	9.851	Caffeoylquinic acid hexoside (II) (CafQAHex II)<br>$C_{22}H_{28}O_{14}$<br>Mw=516.1479	515.1408<br>-1.5 ppm	$[M - H - Caffeoyl - Hex]^-$<br>$[M - H - Caffeoyl - Hex - H_2O]^-$<br>$[M - H - Quinoyl - Hex]^-$<br>$[M - H - Hex]^-$	191.055 ($C_7H_{11}O_6$)<br>173.045 ($C_7H_9O_5$)<br>179.034 ($C_9H_7O_4$)<br>353.087 ($C_{16}H_{17}O_9$)	2b
**30**	10.389	Caffeoylquinic acid (I) (CafQA I)<br>$C_{16}H_{18}O_9$<br>Mw=354.0951	353.0879<br>-1.9 ppm	$[M - H - Caffeoyl]^-$<br>$[M - H - Quinoyl - CO_2]^-$<br>$[M - H - Quinoyl]^-$	191.055 ($C_7H_{11}O_6$)<br>135.044 ($C_8H_7O_2$)<br>179.034 ($C_9H_7O_4$)	1<br>Lin and Harnly, 2009
**31**	10.478	Feruloylquinic acid hexoside (FerQAHex)<br>$C_{23}H_{30}O_{14}$<br>Mw=530.1636	529.1563<br>-1.1 ppm	$[M - H - Hex - Feruloyl]^-$<br>$[M - H - Hex - Quinoyl]^-$<br>$[M - H - Hex]^-$	191.055 ($C_7H_{11}O_6$)<br>193.049 ($C_{10}H_9O_4$)<br>367.103 ($C_{17}H_{19}O_9$)	2b
**32**	10.798	Sinapoyl-caffeoylquinic acid pentoside hexoside<br>(Sin-CafQAPentHex)<br>$C_{38}H_{48}O_{23}$<br>Mw=872.2586	871.2521<br>-1.5 ppm	$[M - H - Hex - Sinapoyl - H_2O]^-$<br>$[M - H - Hex]^-$<br>$[M - H - Hex - Sinapoyl - Pent - H_2O]^-$<br>$[M - H - Hex - Sinapoyl - Quinoyl - Pent - 2H_2O]^-$<br>$[M - H - Hex - CQ - Pent - H_2O]^-$	485.130 ($C_{21}H_{25}O_{13}$)<br>709.199 ($C_{32}H_{37}O_{18}$)<br>353.088 ($C_{16}H_{17}O_9$)<br>161.023 ($C_9H_5O_3$)<br>223.060 ($C_{11}H_{11}O_5$)	2b
**33**	10.983	*p*-Coumaroylquinic acid (I) (CoumQA I)<br>$C_{16}H_{18}O_8$<br>Mw=338.1002	337.0923<br>0.1 ppm	$[M - H - Quinoyl - CO_2]^-$<br>$[M - H - Coumaroyl]^-$<br>$[M - H - Quinoyl]^-$	119.04898 (C_8H_7O)<br>191.05537 ($C_7H_{11}O_6$)<br>163.03912 ($C_9H_7O_3$)	2b<br>Lin and Harnly, 2009
**34**	11.044	Caffeoylquinic acid (II) (CafQA II)<br>$C_{16}H_{18}O_9$<br>Mw=354.0951	353.0879<br>-1.8 ppm	$[M - H - Caffeoyl]^-$<br>$[M - H - Quinoyl - H_2O]^-$<br>$[M - H - Quinoyl - CO_2]^-$<br>$[M - H - Quinoyl]^-$	191.055 ($C_7H_{11}O_6$)<br>161.023 ($C_9H_5O_3$)<br>135.044 ($C_8H_7O_2$)<br>179.034 ($C_9H_7O_4$)	1<br>Lin and Harnly, 2009
**35**	11.126	Sinapoyl-caffeoylquinic acid hexoside (I)<br>(Sin-CafQAHex I)<br>$C_{33}H_{40}O_{19}$<br>Mw=740.2164	739.2100<br>-1.9 ppm	$[M - H - Hex - Sinapoyl - Caffeoyl - H_2O]^-$<br>$[M - H - Hex - Sinapoyl - H_2O]^-$<br>$[M - H - Hex - CQ - CH_2 - 2H_2O]^-$<br>$[M - H - Hex - CQ - CH_2 - H_2O]^-$<br>$[M - H - Hex - CQ - H_2O]^-$	191.055 ($C_7H_{11}O_6$)<br>353.088 ($C_{16}H_{17}O_9$)<br>191.035 ($C_{10}H_7O_4$)<br>209.045 ($C_{10}H_9O_5$)<br>223.061 ($C_{11}H_{11}O_5$)	2b
**36**	11.167	Feruloylquinic acid (I) (FerQA I)<br>$C_{17}H_{20}O_9$<br>Mw=368.1107	367.1036<br>-1.9 ppm	$[M - H - Quinoyl - CO_2 - CH_3]^-$<br>$[M - H - Quinoyl]^-$<br>$[M - H - Quinoyl - CO_2]^-$	134.036 ($C_8H_6O_2$)<br>193.049 ($C_{10}H_9O_4$)<br>149.059 ($C_9H_9O_2$)	2b<br>Lin and Harnly, 2009
**37**	11.222	Caffeoylquinic acid (III) (CafQA III)<br>$C_{16}H_{18}O_9$<br>Mw=354.0951	353.0871<br>0.4 ppm	$[M - H - Caffeoyl]^-$<br>$[M - H - Quinoyl - H_2O]^-$	191.055 ($C_7H_{11}O_6$)<br>161.023 ($C_9H_5O_3$)	2b<br>Lin and Harnly, 2009

**38**	11.489	Hydroxyferuloyl-caffeoylquinic acid hexoside (hFer-CafQAHex)<br>$C_{32}H_{38}O_{19}$<br>Mw=726.2007	725.1942<br>-1.8 ppm	M − H − Hex − CQ − $H_2O]^-$<br>[M − H − h-Feruloyl − $H_2O]^-$<br>[M − H − Hex − CQ − $2H_2O]^-$<br>[M − H − h-Feruloyl$]^-$	209.045 ($C_{10}H_9O_5$)<br>515.141 ($C_{22}H_{27}O_{14}$)<br>191.034 ($C_{10}H_7O_4$)<br>533.153 ($C_{22}H_{29}O_{15}$)	2b
**39**	11.557	*p*-Coumaroylquinic acid (II) (CoumQA II)<br>$C_{16}H_{18}O_8$<br>Mw=338.1002	337.0929<br>-1.7 ppm	[M − H − Coumaroyl − $H_2O]^-$<br>[M − H − Quinoyl − $CO_2]^-$<br>[M − H − Quinoyl$]^-$<br>[M − H − Coumaroyl$]^-$	173.045 ($C_7H_9O_5$)<br>119.049 (C_8H_7O)<br>163.039 ($C_9H_7O_3$)<br>191.055 ($C_7H_{11}O_6$)	2b
**40**	11.599	Disinapoyl-coumaroylquinic acid dihexoside (2Sin-CoumQA2Hex)<br>$C_{50}H_{60}O_{27}$<br>Mw=1092.3322	1091.3252<br>-0.7 ppm	[M − H − Hex$]^-$<br>[M − H − 2Hex − 2Sinapoyl − $H_2O]^-$<br>[M − H − Hex − Sinapoyl − $H_2O]^-$	929.272 ($C_{44}H_{49}O_{22}$)<br>337.093 ($C_{16}H_{17}O_8$)<br>705.204 ($C_{33}H_{37}O_{17}$)	2b
**41**	11.609	Sinapoyl-dicoumaroylquinic acid hexoside (Sin-2CoumQAHex)<br>$C_{49}H_{58}O_{26}$<br>Mw=1062.3216	1061.3149<br>-1.0 ppm	[M − H − Hex$]^-$<br>[M − H − Hex − Sinapoyl − $H_2O]^-$<br>[M − H − Sinapoyl$]^-$<br>[M − H − Sinapoyl − $H_2O]^-$	899.261 ($C_{43}H_{47}O_{21}$)<br>675.193 ($C_{32}H_{35}O_{16}$)<br>855.259 ($C_{38}H_{47}O_{22}$)<br>837.246 ($C_{38}H_{45}O_{21}$)	2b
**42**	11.723	Feruloylquinic acid (II) (FerQA II)<br>$C_{17}H_{20}O_9$<br>Mw=368.1107	367.1036<br>-1.9 ppm	[M − H − Feruloyl − $H_2O]^-$<br>[M − H − Quinoyl − CO_2 − $CH_3]^-$<br>[M − H − Quinoyl$]^-$	173.045 ($C_7H_9O_5$)<br>134.036 ($C_8H_6O_2$)<br>193.049 ($C_{10}H_9O_4$)	2b
**43**	11.843	*p*-Coumaroylquinic acid (III) (CoumQA III)<br>$C_{16}H_{18}O_8$<br>Mw=338.1002	337.0925<br>-0.5 ppm	[M − H − Coumaroyl$]^-$<br>[M − H − Quinoyl − $CO_2]^-$<br>[M − H − Quinoyl$]^-$	191.055 ($C_7H_{11}O_6$)<br>119.049 (C_8H_7O)<br>163.039 ($C_9H_7O_3$)	2b
**44**	11.911	Disinapoyl-caffeoylquinic acid dihexoside (2Sin-CafQA2Hex)<br>$C_{50}H_{60}O_{28}$<br>Mw=1108.3271	1107.3196<br>-0.3 ppm	[M − H − Hex − Sinapoyl$]^-$<br>[M − H − Hex − 2Sinapoyl − $H_2O]^-$<br>[M − H − Hex − Sinapoyl − CQ −$2H_2O]^-$<br>[M − H − 2Hex − 2Sinapoyl − Caffeoyl − $H_2O]^-$<br>[M − H − 2Hex − Sinapoyl − CQ − $H_2O]^-$	739.209 ($C_{33}H_{39}O_{19}$)<br>515.141 ($C_{22}H_{27}O_{14}$)<br>367.103 ($C_{17}H_{19}O_9$)<br>191.055 ($C_7H_{11}O_6$)<br>223.061 ($C_{11}H_{11}O_5$)	2b
**45**	11.934	Feruloylquinic acid (III) (FerQA III)<br>$C_{17}H_{20}O_9$<br>Mw=368.1107	367.1035<br>-1.8 ppm	[M − H − Feruloyl$]^-$<br>[M − H − Quinoyl − CO_2 − $CH_3]^-$<br>[M − H − Quinoyl$]^-$<br>[M − H − Feruloyl − $H_2O]^-$	191.055 ($C_7H_{11}O_6$)<br>134.036 ($C_8H_6O_2$)<br>193.049 ($C_{10}H_9O_4$)<br>173.045 ($C_7H_9O_5$)	2b
**46**	11.95	Syringaldehyde-sinapoylquinic acid hexoside (SyringAld-SinQAHex)<br>$C_{33}H_{40}O_{18}$<br>Mw=724.2215	723.2147<br>-1.5 ppm	[M − H − Hex − Sinapoyl − SyringAld$]^-$<br>[M − H − Quinoyl − SyringAld − $H_2O]^-$<br>[M − H − Hex − Sinapoyl − SyringAld − $H_2O]^-$<br>[M − H − Sinapoyl − Quinoyl − 2CO − $H_2O]^-$	191.055 ($C_7H_{11}O_6$)<br>367.103 ($C_{17}H_{19}O_9$)<br>173.044 ($C_7H_9O_5$)<br>269.103 ($C_{13}H_{17}O_6$)	3
**47**	12.004	Feruloyl-caffeoylquinic acid hexoside (I) (Fer-CafQAHex I)<br>$C_{32}H_{38}O_{18}$<br>Mw=710.2058	709.1991<br>-1.6 ppm	[M − H − Feruloyl − $H_2O]^-$<br>[M − H − Hex − Quinoyl − Feruloyl − $2H_2O]^-$<br>[M − H − Hex − CQ − $H_2O]^-$	515.141 ($C_{22}H_{27}O_{14}$)<br>161.023 ($C_9H_5O_3$)<br>193.049 ($C_{10}H_9O_4$)	2b
**48**	12.216	Sinapoyl-caffeoylquinic acid hexoside (II) (Sin-CafQAHex II)<br>$C_{33}H_{40}O_{19}$<br>Mw=740.2164	739.2099<br>-1.8 ppm	[M − H − Sinapoyl − $H_2O]^-$<br>[M − H − Hex − CQ − CH_2 − $2H_2O]^-$<br>[M − H − Hex − CQ − CH_2 − $H_2O]^-$<br>[M − H − Hex − CQ − $H_2O]^-$	515.141 ($C_{22}H_{27}O_{14}$)<br>191.034 ($C_{10}H_7O_4$)<br>209.045 ($C_{10}H_9O_5$)<br>223.061 ($C_{11}H_{11}O_5$)	2b
**49**	12.257	Sinapoyl-hydroferuloyl-caffeoylquinic acid hexoside (Sin-hFer-CafQAHex)<br>$C_{43}H_{48}O_{23}$<br>Mw=932.2586	931.2520<br>-1.3 ppm	[M − H − h-Feruloyl($C_{10}H_8O_4$) − $H_2O]^-$<br>[M − H − Hex − CQ − Sinapoyl − $H_2O]^-$<br>[M − H − Hex − CQ − Sinapoyl − $2H_2O]^-$<br>[M − H − h-Feruloyl − Sinapoyl − $H_2O]^-$<br>[M − H − h-Feruloyl$]^-$	721.199 ($C_{33}H_{37}O_{18}$)<br>209.045 ($C_{10}H_9O_5$)<br>191.034 ($C_{10}H_7O_4$)<br>515.140 ($C_{22}H_{27}O_{14}$)<br>739.210 ($C_{33}H_{39}O_{19}$)	2b
**50**	12.267	Feruloyl-caffeoylquinic acid hexoside (II)<br>(Fer-CafQAHex II)<br>$C_{32}H_{38}O_{18}$<br>Mw=710.2058	709.1992<br>-1.8 ppm	[M − H − Feruloyl − $H_2O]^-$<br>[M − H − Hex − CQ − $H_2O]^-$	515.141 ($C_{22}H_{27}O_{14}$)<br>193.050 ($C_{10}H_9O_4$)	2b
**51**	12.628	Disinapoyl-caffeoylquinic acid hexoside (I) (2Sin-CafQAHex I)<br>$C_{44}H_{50}O_{23}$<br>Mw=946.2743	945.2674<br>-1.0 ppm	[M − H − Sinapoyl − $H_2O]^-$<br>[M − H − 2Sinapoyl − $H_2O]^-$	721.199 ($C_{33}H_{37}O_{18}$)<br>515.141 ($C_{22}H_{27}O_{14}$)	2b
**52**	12.736	Dihydroferuloyl-disinapoyl-coumaroylquinic acid hexoside (dhFer-2Sin-CoumQAHex)<br>$C_{54}H_{62}O_{26}$<br>Mw=1126.3529	1125.3459<br>-0.7 ppm	[M − H − dh-Feruloyl − $H_2O]^-$<br>[M − H − Sinapoyl$]^-$<br>[M − H − Sinapoyl − $H_2O]^-$<br>[M − H − Hex − dh-Feruloyl − Sinapoyl − CQ − $3H_2O]^-$<br>[M − H − dh-Feruloyl − Sinapoyl − $2H_2O]^-$	929.272 ($C_{44}H_{49}O_{22}$)<br>919.288 ($C_{43}H_{51}O_{22}$)<br>901.278 ($C_{43}H_{49}O_{21}$)<br>205.04997 ($C_{11}H_9O_4$)<br>705.207 ($C_{33}H_{37}O_{17}$)	3
**53**	12.776	Feruloyl-sinapoyl-caffeoylquinic acid hexoside (I) (Fer-Sin-CafQAHex I)<br>$C_{43}H_{48}O_{22}$<br>Mw=916.2637	915.2570<br>-1.3 ppm	[M − H − Feruloyl − $H_2O]^-$<br>[M − H − Sinapoyl − $H_2O]^-$<br>[M − H − Sinapoyl$]^-$<br>[M − H − Feruloyl − Sinapoyl − $H_2O]^-$	721.198 ($C_{33}H_{37}O_{18}$)<br>691.189 ($C_{32}H_{35}O_{17}$)<br>709.199 ($C_{32}H_{37}O_{18}$)<br>515.140 ($C_{22}H_{27}O_{14}$)	2b

**54**	12.88	Feruloyl-caffeoylquinic acid (Fer-CafQA)<br>$C_{26}H_{28}O_{13}$<br>Mw=548.1529	547.1450<br>0.3 ppm	M − H − CQ − H_2O]$^-$<br>[M − H − Feruloyl − H_2O]$^-$	193.049 ($C_{10}H_9O_4$)<br>353.088 ($C_{16}H_{17}O_9$)	2b
**55**	13.06	Disinapoyl-caffeoylquinic acid hexoside (II) (2Sin-CafQAHex II)<br>$C_{44}H_{50}O_{23}$<br>Mw=946.2743	945.2673<br>-0.9 ppm	[M − H − Sinapoyl − H_2O]$^-$<br>[M − H − Hex − Sinapoyl − CQ − CH_2 − $2H_2O$]$^-$<br>[M − H − Sinapoyl]$^-$<br>[M − H − Hex − Sinapoyl − CQ − CH_2 − H_2O]$^-$<br>[M − H − 2Sinapoyl − H_2O]$^-$	721.199 ($C_{33}H_{37}O_{18}$)<br>191.034 ($C_{10}H_7O_4$)<br>739.209 ($C_{33}H_{39}O_{19}$)<br>209.045 ($C_{10}H_9O_5$)<br>515.140 ($C_{22}H_{27}O_{14}$)	2b
**56**	13.178	Feruloyl-sinapoyl-caffeoylquinic acid hexoside (II) (Fer-Sin-CafQAHex II)<br>$C_{43}H_{48}O_{22}$<br>Mw=916.2637	915.2569<br>-1.1 ppm	[M − H − Feruloyl − H_2O]$^-$<br>[M − H − Hex − Feruloyl − CQ − CH_2 − $2H_2O$]$^-$<br>[M − H − Sinapoyl − H_2O]$^-$<br>[M − H − Hex − Feruloyl − CQ − CH_2 − H_2O]$^-$<br>[M − H − Sinapoyl]$^-$<br>[M − H − Feruloyl − Sinapoyl − H_2O]$^-$	721.199 ($C_{33}H_{37}O_{18}$)<br>191.034 ($C_{10}H_7O_4$)<br>691.187 ($C_{32}H_{35}O_{17}$)<br>209.045 ($C_{10}H_9O_5$)<br>709.199 ($C_{32}H_{37}O_{18}$)<br>515.141 ($C_{22}H_{27}O_{14}$)	2b
**57**	13.496	Disinapoyl-caffeoylquinic acid hexoside (III) (2Sin-CafQAHex III)<br>$C_{44}H_{50}O_{23}$<br>Mw=946.2743	945.2674<br>-1.0 ppm	[M − H − Sinapoyl − H_2O]$^-$<br>[M − H − Hex − Sinapoyl − CQ − CH_2 − $2H_2O$]$^-$<br>[M − H − Hex − Sinapoyl − CQ − CH_2 − H_2O]$^-$<br>[M − H − 2Sinapoyl − H_2O]$^-$	721.199 ($C_{33}H_{37}O_{18}$)<br>191.034 ($C_{10}H_7O_4$)<br>209.045 ($C_{10}H_9O_5$)<br>515.141 ($C_{22}H_{27}O_{14}$)	2b
				**Flavonols**		
**58**	9.536	Quercetin tetrahexoside (Quer4Hex)<br>$C_{39}H_{50}O_{27}$<br>Mw=950.2539	949.2471<br>-1.0 ppm	[M − H − 2Hex]$^-$<br>[M − H − Hex]$^-$<br>[M − H − 4Hex]$^-$	625.141 ($C_{27}H_{29}O_{17}$)<br>787.194 ($C_{33}H_{39}O_{22}$)<br>301.034 ($C_{15}H_9O_7$)	2b<br>Lin and Harnly, 2009;<br>Schmidt et al., 2010;<br>Baky et al., 2025
**59**	9.818	Quercetin trihexoside (Quer3Hex)<br>$C_{33}H_{40}O_{22}$<br>Mw=788.2011	787.1943<br>-1.3 ppm	[M − H − Hex]$^-$<br>[M − H − 2Hex]$^-$<br>[M − H − 3Hex]$^-$	625.141 ($C_{27}H_{29}O_{17}$)<br>463.090 ($C_{21}H_{19}O_{12}$)<br>301.035 ($C_{15}H_9O_7$)	2b<br>Lin and Harnly, 2009;<br>Schmidt et al., 2010;<br>Baky et al., 2025
**60**	9.868	Quercetin rhamnoside trihexoside (QuerRham3Hex)<br>$C_{39}H_{50}O_{26}$<br>Mw=934.2590	933.2519<br>-0.8 ppm	[M − H − 2Hex]$^-$<br>[M − H − 2Hex]$^-$<br>[M − H − Hex]$^-$	609.146 ($C_{27}H_{29}O_{16}$)<br>463.051 ($C_{20}H_{15}O_{13}$)<br>771.199 ($C_{33}H_{39}O_{21}$)	2b
**61**	9.969	Kaempferol trihexoside (I) (Kamp3Hex I)<br>$C_{33}H_{40}O_{21}$<br>Mw=772.2062	771.1996<br>-1.6 ppm	[M − H − Hex]$^-$<br>[M − H − 3Hex]$^-$<br>[M − H − 2Hex]$^-$	609.146 ($C_{27}H_{29}O_{16}$)<br>285.040 ($C_{15}H_9O_6$)<br>447.093 ($C_{21}H_{19}O_{11}$)	2b<br>Lin and Harnly, 2009;<br>Schmidt et al., 2010;<br>Baky et al., 2025
**62**	10.014	Isorhamnetin trihexoside (Irham3Hex)<br>$C_{34}H_{42}O_{22}$<br>Mw=802.2168	801.2099<br>-1.2 ppm	[M − H − Hex]$^-$<br>[M − H − 2Hex]$^-$	639.157 ($C_{28}H_{31}O_{17}$)<br>477.104 ($C_{22}H_{21}O_{12}$)	2b<br>Schmidt et al., 2010;<br>Baky et al., 2025
**63**	10.078	Kaempferol-hydrokysferuloyl pentahexoside (Kamp-hFer5Hex)<br>$C_{55}H_{68}O_{35}$<br>Mw=1288.3541	[M-2H]$^{-2}$<br>643.1709<br>1.9 ppm	[M − H − 5Hex($C_6H_{10}O_5$) − h-Feruloyl − H]$^-$<br>[M − H − 5Hex($C_6H_{10}O_5$) − Kamp]$^-$<br>[M − H − 2Hex($C_6H_{10}O_5$) − h-Feruloyl]$^-$<br>[M − H − 5Hex($C_6H_{10}O_5$) − h-Feruloyl]$^-$	284.033 ($C_{15}H_8O_6$)<br>191.034 ($C_{10}H_7O_4$)<br>771.199 ($C_{33}H_{39}O_{21}$)<br>285.037 ($C_{15}H_9O_6$)	2b<br>Baky et al., 2025
**64**	10.101	Quercetin-hydroxyferuloyl tetrahexoside (Quer-hFer4Hex)<br>$C_{49}H_{58}O_{31}$<br>Mw=1142.2962	1141.2890<br>-0.6 ppm	[M − H − h-Feruloyl]$^-$<br>[M − H − 2Hex($C_6H_{10}O_5$)]$^-$<br>[M − H − $C_{10}H_8O_5$]$^-$<br>[M − H − 2Hex − h-Feruloyl]$^-$	949.247 ($C_{39}H_{49}O_{27}$)<br>817.184 ($C_{37}H_{37}O_{21}$)<br>933.249 ($C_{39}H_{49}O_{26}$)<br>625.142 ($C_{27}H_{29}O_{17}$)	2b<br>Lin and Harnly, 2009;<br>Olsen et al., 2009;<br>Schmidt et al., 2010;<br>Baky et al., 2025
**65**	10.129	Quercetin-caffeoyl tetrahexoside (Quer-Caf4Hex)<br>$C_{48}H_{56}O_{30}$<br>Mw=1112.2856	1111.2784<br>-0.5 ppm	[M − H − Caffeoyl]$^-$<br>[M − H − 2Hex]$^-$<br>[M − H − 2Hex − Caffeoyl]$^-$	949.247 ($C_{39}H_{49}O_{27}$)<br>787.175 ($C_{36}H_{35}O_{20}$)<br>625.143 ($C_{27}H_{29}O_{17}$)	2b<br>Lin and Harnly, 2009;<br>Olsen et al., 2009;<br>Schmidt et al., 2010;<br>Baky et al., 2025
**66**	10.133	Kaempferol-caffeoyl pentahexoside (Kamp-Caf5Hex)<br>$C_{54}H_{66}O_{34}$<br>Mw=1258.3436	[M-2H]$^{-2}$<br>628.1651<br>-1.9 ppm	[M − H − 5Hex − Caffeoyl − H]$^-$<br>[M − H − 5Hex − Kamp]$^-$<br>[M − H − 2Hex − Caffeoyl]$^-$<br>[M − H − 5Hex − Caffeoyl]$^-$	284.033 ($C_{15}H_8O_6$)<br>161.023 ($C_9H_5O_3$)<br>771.199 ($C_{33}H_{39}O_{21}$)<br>285.037 ($C_{15}H_9O_6$)	2b<br>Schmidt et al., 2010;<br>Baky et al., 2025
**67**	10.137	Quercetin-sinapoyl pentahexoside (Quer-Sin5Hex)<br>$C_{56}H_{70}O_{36}$<br>Mw=1318.3647	[M-2H]$^{-2}$<br>658.1758<br>-1.9 ppm	[M − H − 2Hex − Sinapoyl]$^-$<br>[M − H − 5Hex − Sinapoyl − H]$^-$<br>[M − H − 5Hex − Quer]$^-$<br>[M − H − 2Hex]$^-$<br>[M − H − 5Hex − Sinapoyl]$^-$	787.195 ($C_{33}H_{39}O_{22}$)<br>300.028 ($C_{15}H_8O_7$)<br>205.050 ($C_{11}H_9O_4$)<br>993.252 ($C_{44}H_{49}O_{26}$)<br>301.033 ($C_{15}H_9O_7$)	2b<br>Schmidt et al., 2010;<br>Baky et al., 2025
**68**	10.139	Eriodictyol dihexoside (Erio2Hex)<br>$C_{27}H_{32}O_{16}$<br>Mw=612.1690	611.1620<br>-1.4 ppm	[M − H − Hex]$^-$<br>[M − H − 2Hex]$^-$	449.109 ($C_{21}H_{21}O_{11}$)<br>287.056 ($C_{15}H_{11}O_6$)	2b
**69**	10.222	Kaempferol tetrahexoside (Kamp4Hex)<br>$C_{39}H_{50}O_{26}$<br>Mw=934.2590	933.2519<br>-0.8 ppm	[M − H − 2Hex]$^-$<br>[M − H − 4Hex]$^-$	609.146 ($C_{27}H_{29}O_{16}$)<br>285.041 ($C_{15}H_9O_6$)	2b<br>Lin and Harnly, 2009;<br>Schmidt et al., 2010;<br>Baky et al., 2025
**70**	10.239	Kaempferol-hydroxyferuloyl tetrahexoside (Kamp-hFer4Hex)<br>$C_{49}H_{58}O_{30}$<br>Mw=1126.3013	[M-2H]$^{-2}$<br>562.1477<br>-0.3 ppm	[M − H − 2Hex]$^-$<br>[M − H − Hex]$^-$<br>[M − H − h-Feruloyl − 2Hex]$^-$<br>[M − H − 4Hex − Kamp]$^-$<br>[M − H − h-Feruloyl − 4Hex]$^-$	801.189 ($C_{37}H_{37}O_{20}$)<br>963.241 ($C_{43}H_{47}O_{25}$)<br>609.147($C_{27}H_{29}O_{16}$)<br>191.034 ($C_{10}H_7O_4$)<br>285.037 ($C_{15}H_9O_6$)	2b<br>Lin and Harnly, 2009;<br>Olsen et al., 2009;<br>Schmidt et al., 2010;<br>Baky et al., 2025
**71**	10.254	Quercetin-hydroxyferuloyl trihexoside (Quer-hFer3Hex)<br>$C_{43}H_{48}O_{26}$	979.2349<br>0.7 ppm	[M − H − h-Feruloyl]$^-$<br>[M − H − Hex]$^-$<br>[M − H − h-Feruloyl − Hex]$^-$	787.194 ($C_{33}H_{39}O_{22}$)<br>817.184 ($C_{37}H_{37}O_{21}$)<br>625.142 ($C_{27}H_{29}O_{17}$)	2b<br>Lin and Harnly, 2009;<br>Olsen et al., 2009;

		Mw=980.2434				Schmidt et al., 2010;<br>Baky et al., 2025
**72**	10.264	Kaempferol-sinapoyl pentahexoside<br>(Kamp-Sin5Hex)<br>$C_{56}H_{70}O_{35}$<br>Mw=1302.3698	$[M-2H]^{-2}$<br>650.1782<br>-1.0 ppm	$[M - H - 5Hex - Kamp]^{-}$<br>$[M - H - 2Hex - Sinapoyl]^{-}$<br>$[M - H - 5Hex - Sinapoyl]^{-}$	205.050 ($C_{11}H_9O_4$)<br>771.199 ($C_{33}H_{39}O_{21}$)<br>285.038 ($C_{15}H_9O_6$)	2b<br>Schmidt et al., 2010
**73**	10.291	Kaempferol-caffeoyl tetrahexoside<br>(Kamp-Caf4Hex)<br>$C_{48}H_{56}O_{29}$<br>Mw=1096.2907	1095.2835<br>-0.6 ppm	$[M - H - Hex - Caffeoyl]^{-}$<br>$[M - H - 2Hex - Caffeoyl]^{-}$<br>$[M - H - Hex]^{-}$	771.178 ($C_{33}H_{39}O_{21}$)<br>609.145 ($C_{27}H_{29}O_{16}$)<br>933.231 ($C_{42}H_{45}O_{24}$)	2b<br>Lin and Harnly, 2009;<br>Olsen et al., 2009;<br>Schmidt et al., 2010;<br>Baky et al., 2025
**74**	10.315	Quercetin-sinapoyl tetrahexoside<br>(Quer-Sin4Hex)<br>$C_{50}H_{60}O_{31}$<br>Mw=1156.3119	$[M-2H]^{-2}$<br>577.1492<br>-0.8 ppm	$[M - H - Sinapoyl]^{-}$<br>$[M - H - 2Hex]^{-}$<br>$[M - H - 2Hex - Sinapoyl]^{-}$	949.246 ($C_{39}H_{49}O_{27}$)<br>831.199 ($C_{38}H_{39}O_{21}$)<br>625.141 ($C_{27}H_{29}O_{17}$)	2b<br>Lin and Harnly, 2009;<br>Olsen et al., 2009;<br>Schmidt et al., 2010;<br>Baky et al., 2025
**75**	10.338	Kaempferol-feruloyl pentahexoside<br>(Kamp-Fer5Hex)<br>$C_{55}H_{68}O_{34}$<br>Mw=1272.3592	$[M-2H]^{-2}$<br>635.1729<br>-0.9 ppm	$[M - H - 5Hex - Kamp]^{-}$<br>$[M - H - 2Hex - Feruloyl]^{-}$<br>$[M - H - 2Hex]^{-}$<br>$[M - H - 5Hex - Feruloyl]^{-}$	175.039 ($C_{10}H_7O_3$)<br>771.199 ($C_{33}H_{39}O_{21}$)<br>947.246 ($C_{43}H_{47}O_{24}$)<br>285.038 ($C_{15}H_9O_6$)	2b<br>Schmidt et al., 2010;<br>Baky et al., 2025
**76**	10.344	Kaempferol trihexoside (II)<br>(Kamp3Hex II)<br>$C_{33}H_{40}O_{21}$<br>Mw=772.2062	771.1994<br>-1.3 ppm	$[M - H - 2Hex]^{-}$<br>$[M - H - Hex]^{-}$<br>$[M - H - 3Hex]^{-}$	447.094 ($C_{21}H_{19}O_{11}$)<br>609.146 ($C_{27}H_{29}O_{16}$)<br>285.040 ($C_{15}H_9O_6$)	2b<br>Lin and Harnly, 2009;<br>Schmidt et al., 2010;<br>Baky et al., 2025
**77**	10.42	Kaempferol-hydroxyferuloyl trihexoside<br>(Kamp-hFer3Hex)<br>$C_{43}H_{48}O_{25}$<br>Mw=964.2485	963.2413<br>-0.6 ppm	$[M - H - Hex]^{-}$<br>$[M - H - Hex - h\text{-}Feruloyl]^{-}$<br>$[M - H - 3Hex - Kamp]^{-}$	801.189 ($C_{37}H_{37}O_{20}$)<br>609.146 ($C_{27}H_{29}O_{16}$)<br>191.034 ($C_{10}H_7O_4$)	2b<br>Lin and Harnly, 2009;<br>Olsen et al., 2009;<br>Schmidt et al., 2010;<br>Baky et al., 2025
**78**	10.474	Kaempferol-sinapoyl tetrahexoside<br>(Kamp-Sin4Hex)<br>$C_{50}H_{60}O_{30}$<br>Mw=1140.3169	1139.3094<br>-0.2 ppm	$[M - H - 2Hex]^{-}$<br>$[M - H - Hex]^{-}$<br>$[M - H - 2Hex - Sinapoyl]^{-}$	815.204 ($C_{38}H_{39}O_{20}$)<br>977.258 ($C_{44}H_{49}O_{25}$)<br>609.146 ($C_{27}H_{29}O_{16}$)	2b<br>Lin and Harnly, 2009;<br>Olsen et al., 2009;<br>Schmidt et al., 2010;<br>Baky et al., 2025
**79**	10.496	Quercetin-sinapoyl trihexoside<br>(Quer-Sin3Hex)<br>$C_{44}H_{50}O_{26}$<br>Mw=994.2590	993.2519<br>-0.7 ppm	$[M - H - Hex]^{-}$<br>$[M - H - Sinapoyl]^{-}$<br>$[M - H - Hex - Sinapoyl]^{-}$<br>$[M - H - 2Hex - Sinapoyl - H]^{-}$	831.199 ($C_{38}H_{39}O_{21}$)<br>787.194 ($C_{33}H_{39}O_{22}$)<br>625.142 ($C_{27}H_{29}O_{17}$)<br>462.080 ($C_{21}H_{18}O_{12}$)	2b<br>Lin and Harnly, 2009;<br>Olsen et al., 2009;<br>Schmidt et al., 2010;<br>Baky et al., 2025
**80**	10.496	Kaempferol-caffeoyl trihexoside<br>(Kamp-Caf3Hex)<br>$C_{42}H_{46}O_{24}$<br>Mw=934.2379	933.2309<br>-0.9 ppm	$[M - H - Hex]^{-}$<br>$[M - H - Hex - Caffeoyl]^{-}$	771.178 ($C_{36}H_{35}O_{19}$)<br>609.146 ($C_{27}H_{29}O_{16}$)	2b<br>Lin and Harnly, 2009;<br>Olsen et al., 2009;<br>Schmidt et al., 2010;<br>Baky et al., 2025
**81**	10.535	Kaempferol-feruloyl tetrahexoside<br>(Kamp-Fer4Hex)<br>$C_{49}H_{58}O_{29}$<br>Mw=1110.3064	1109.2991<br>-0.5 ppm	$[M - H - 2Hex]^{-}$<br>$[M - H - Hex]^{-}$<br>$[M - H - 2Hex - Feruloyl]^{-}$	785.194 ($C_{37}H_{37}O_{19}$)<br>947.242 ($C_{43}H_{47}O_{24}$)<br>609.147 ($C_{27}H_{29}O_{16}$)	2b<br>Lin and Harnly, 2009;<br>Olsen et al., 2009;<br>Schmidt et al., 2010;<br>Baky et al., 2025
**82**	10.663	Kaempferol-sinapoyl trihexoside<br>(Kamp-Sin3Hex)<br>$C_{44}H_{50}O_{25}$<br>Mw=978.2641	977.2571<br>-0.9 ppm	$[M - H - Hex]^{-}$<br>$[M - H - Hex - Sinapoyl]^{-}$	815.204 ($C_{38}H_{39}O_{20}$)<br>609.146 ($C_{27}H_{29}O_{16}$)	2b<br>Lin and Harnly, 2009;<br>Olsen et al., 2009;<br>Schmidt et al., 2010;<br>Baky et al., 2025
**83**	10.748	Kaempferol-feruloyl trihexoside<br>(Kamp-Fer3Hex)<br>$C_{43}H_{48}O_{24}$<br>Mw=948.2536	947.2466<br>-0.9 ppm	$[M - H - Hex]^{-}$<br>$[M - H - Hex - Feruloyl]^{-}$<br>$[M - H - 3Hex - Feruloyl - H]^{-}$	785.194 ($C_{37}H_{37}O_{19}$)<br>609.147 ($C_{27}H_{29}O_{16}$)<br>284.031 ($C_{15}H_8O_6$)	2b<br>Lin and Harnly, 2009;<br>Olsen et al., 2009;<br>Schmidt et al., 2010;<br>Baky et al., 2025
**84**	10.879	Kaempferol-dihydroxyferuloyl<br>pentahexoside (Kamp-2hFer5Hex)<br>$C_{65}H_{76}O_{39}$<br>Mw=1480.3964	$[M-2H]^{-2}$<br>739.1915<br>-0.8 ppm	$[M - H - 5Hex - h\text{-}Feruloyl - Kamp]^{-}$<br>$[M - H - 2Hex - h\text{-}Feruloyl]^{-}$<br>$[M - H - 5Hex - 2(h\text{-}Feruloyl) - H]^{-}$	191.034 ($C_{10}H_7O_4$)<br>963.241 ($C_{43}H_{47}O_{25}$)<br>284.033 ($C_{15}H_8O_6$)	3
**85**	11.030	Kaempferol-hydroxyferuloyl-sinapoyl<br>pentahexoside (Kamp-hFer-Sin5Hex)<br>$C_{66}H_{78}O_{39}$<br>Mw=1494.4120	$[M-2H]^{-2}$<br>746.1994<br>-1.00 ppm	$[M - H - 5Hex - Sinapoyl - h\text{-}Feruloyl - H]^{-}$<br>$[M - H - 5Hex - Kamp - h\text{-}Feruloyl]^{-}$<br>$[M - H - 5Hex - Kamp - Sinapoyl]^{-}$<br>$[M - H - 2Hex - Sinapoyl]^{-}$	284.032 ($C_{15}H_8O_6$)<br>205.050 ($C_{11}H_9O_4$)<br>191.034 ($C_{10}H_7O_4$)<br>963.242 ($C_{43}H_{47}O_{25}$)	2b
**86**	11.056	Quercetin-disinapoyl pentahexoside<br>(Quer-2Sin5Hex)<br>$C_{67}H_{80}O_{40}$<br>Mw=1524.4226	$[M-2H]^{-2}$<br>761.2046<br>-0.8 ppm	$[M - H - 5Hex - Quer - Sinapoyl]^{-}$<br>$[M - H - 5Hex - 2Sinapoyl - H]^{-}$<br>$[M - H - 2Hex - Sinapoyl]^{-}$	205.050 ($C_{11}H_9O_4$)<br>300.027 ($C_{15}H_8O_7$)<br>993.252 ($C_{44}H_{49}O_{26}$)	2b<br>Schmidt et al., 2010;<br>Baky et al., 2025
**87**	11.084	Kaempferol-sinapoyl trihexoside<br>(hydrated) (Kamp-Sin3Hex-h)<br>$C_{44}H_{52}O_{26}$<br>Mw=996.2747	995.2677<br>-0.9 ppm	$[M - H - Hex]^{-}$<br>$[M - H - 3Hex - Sinapoyl - H_2O]^{-}$<br>$[M - H - Hex - H_2O]^{-}$<br>$[M - H - 2Hex - Sinapoyl - H_2O]^{-}$	833.215 ($C_{38}H_{41}O_{21}$)<br>285.040 ($C_{15}H_9O_6$)<br>815.204 ($C_{38}H_{39}O_{20}$)<br>447.095 ($C_{21}H_{19}O_{11}$)	3
**88**	11.099	Kaempferol-feruloyl trihexoside<br>(hydrated)(Kamp-Fer3Hex-h)<br>$C_{43}H_{50}O_{25}$<br>Mw=966.2641	965.2573<br>-1.0 ppm	$[M - H - Hex]^{-}$<br>$[M - H - 3Hex - Feruloyl - H_2O]^{-}$<br>$[M - H - Hex - H_2O]^{-}$<br>$[M - H - 2Hex - Feruloyl - H_2O]^{-}$	803.205 ($C_{37}H_{39}O_{20}$)<br>285.040 ($C_{15}H_9O_6$)<br>785.193 ($C_{37}H_{37}O_{19}$)<br>447.094 ($C_{21}H_{19}O_{11}$)	3
**89**	11.111	Kaempferol-feruloyl-hydroxyferuloyl<br>pentahexoside	$[M-2H]^{-2}$<br>731.1942	$[M - H - 5Hex - Feruloyl - h\text{-}Feruloyl - H]^{-}$	284.033 ($C_{15}H_8O_6$)	2b<br>Baky et al., 2025

		(Kamp-Fer-hFer5Hex)<br>$C_{65}H_{76}O_{38}$<br>Mw=1464.4015	-1.0 ppm	[M − H − 5Hex − Kamp − h-Feruloyl]$^{-}$<br>[M − H − 5Hex − Kamp − Feruloyl]$^{-}$<br>[M − H − 5Hex − Feruloyl − h-Feruloyl]$^{-}$	175.039 ($C_{10}H_7O_3$)<br>191.034 ($C_{10}H_7O_4$)<br>285.038 ($C_{15}H_9O_6$)	
**90**	11.157	Kaempferol dihexoside (I)<br>(Kamp2Hex I)<br>$C_{27}H_{30}O_{16}$<br>Mw=610.1534	609.1464<br>-1.4 ppm	[M − H − Hex]$^{-}$<br>[M − H − 2Hex]$^{-}$	447.092 ($C_{21}H_{19}O_{11}$)<br>285.041 ($C_{15}H_9O_6$)	2b<br>Lin and Harnly, 2009;<br>Schmidt et al., 2010;<br>Baky et al., 2025
**91**	11.164	Kaempferol-disinapoyl pentohexoside<br>(Kamp-2Sin5Hex)<br>$C_{67}H_{80}O_{39}$<br>Mw=1508.4277	[M-2H]$^{-2}$<br>753.2065<br>0.1 ppm	[M − H − 5Hex − Kamp − Sinapoyl]$^{-}$<br>[M − H − 2Hex − Sinapoyl]$^{-}$<br>[M − H − 5Hex − 2Sinapoyl − H]$^{-}$<br>[M − H − 4Hex − Kamp − Sinapoyl]$^{-}$<br>[M − H − 2Hex]$^{-}$	205.050 ($C_{11}H_9O_4$)<br>977.258 ($C_{44}H_{49}O_{25}$)<br>284.033 ($C_{15}H_8O_6$)<br>367.103 ($C_{17}H_{19}O_9$)<br>1183.315 ($C_{55}H_{59}O_{29}$)	2b<br>Schmidt et al., 2010;<br>Baky et al., 2025
**92**	11.228	Quercetin dihexoside (Quer2Hex)<br>$C_{27}H_{30}O_{17}$<br>Mw=626.1483	625.1414<br>-1.5 ppm	[M − H − 2Hex]$^{-}$<br>[M − H − Hex]$^{-}$	301.033 ($C_{15}H_9O_7$)<br>463.088 ($C_{21}H_{19}O_{12}$)	2b<br>Lin and Harnly, 2009;<br>Schmidt et al., 2010;<br>Baky et al., 2025
**93**	11.239	Kaempferol-sinapoyl-hydroxyferuloyl tetrahexoside (Kamp-Sin-hFer4Hex)<br>$C_{60}H_{68}O_{34}$<br>Mw=1332.3592	[M-2H]$^{-2}$<br>665.1730<br>-1.1 ppm	[M − H − 4Hex − Sinapoyl − h-Feruloyl − H]$^{-}$<br>[M − H − 4Hex − Kamp − h-Feruloyl]$^{-}$<br>[M − H − 4Hex − Kamp − Sinapoyl]$^{-}$<br>[M − H − 4Hex − Sinapoyl − h-Feruloyl − H]$^{-}$	284.033 ($C_{15}H_8O_6$)<br>205.050 ($C_{11}H_9O_4$)<br>191.034 ($C_{10}H_7O_4$)<br>285.037 ($C_{15}H_9O_6$)	2b
**94**	11.265	Quercetin-disinapoyl tetrahexoside<br>(Quer-2Sin4Hex)<br>$C_{61}H_{70}O_{35}$<br>Mw=1362.3698	[M-2H]$^{-2}$<br>680.1781<br>-0.8 ppm	[M − H − 4Hex − 2Sinapoyl − H]$^{-}$<br>[M − H − 4Hex − Quer − Sinapoyl]$^{-}$	300.028 ($C_{15}H_8O_7$)<br>205.050 ($C_{11}H_9O_4$)	2b<br>Schmidt et al., 2010;<br>Baky et al., 2025
**95**	11.266	Kaempferol-sinapoyl-feruloyl pentahexoside (Kamp-Sin-Fer5Hex)<br>$C_{66}H_{78}O_{38}$<br>Mw=1478.4171	[M-2H]$^{-2}$<br>738.2019<br>-0.9 ppm	[M − H − 5Hex − Sinapoyl − Feruloyl − H]$^{-}$<br>[M − H − 5Hex − Kamp − Sinapoyl]$^{-}$<br>[M − H − 5Hex − Kamp − Feruloyl]$^{-}$<br>[M − H − 2Hex − Feruloyl]$^{-}$<br>[M − H − 2Hex]$^{-}$<br>[M − H − 2Hex − Sinapoyl]$^{-}$	284.033 ($C_{15}H_8O_6$)<br>175.039 ($C_{10}H_7O_3$)<br>205.050 ($C_{11}H_9O_4$)<br>977.257 ($C_{44}H_{49}O_{25}$)<br>1153.304 ($C_{54}H_{57}O_{28}$)<br>947.247 ($C_{43}H_{47}O_{24}$)	2b<br>Schmidt et al., 2010;<br>Baky et al., 2025
**96**	11.368	Kaempferol-diferuloyl pentahexoside<br>(Kamp-2Fer5Hex)<br>$C_{65}H_{76}O_{37}$<br>Mw=1448.4065	[M-2H]$^{-2}$<br>723.1965<br>-0.7 ppm	[M − H − 5Hex − Kamp − Feruloyl]$^{-}$<br>[M − H − 5Hex − 2Feruloyl − H]$^{-}$<br>[M − H − 2Hex − Feruloyl]$^{-}$<br>[M − H − 2Hex]$^{-}$	175.039 ($C_{10}H_7O_3$)<br>284.033 ($C_{15}H_8O_6$)<br>947.247 ($C_{43}H_{47}O_{24}$)<br>1123.295 ($C_{53}H_{55}O_{27}$)	2b<br>Schmidt et al., 2010;<br>Baky et al., 2025
**97**	11.389	Kaempferol-disinapoyl tetrahexoside<br>(Kamp-2Sin4Hex)<br>$C_{61}H_{70}O_{34}$<br>Mw=1346.3749	[M-2H]$^{-2}$<br>672.1812<br>-1.6 ppm	[M − H − 4Hex − Kamp − Sinapoyl]$^{-}$<br>[M − H − Hex − Sinapoyl]$^{-}$<br>[M − H − 4Hex − 2Sinapoyl]$^{-}$<br>[M − H − 4Hex − Kamp − Sinapoyl]$^{-}$	205.050 ($C_{11}H_9O_4$)<br>977.257 ($C_{44}H_{49}O_{25}$)<br>285.037 ($C_{15}H_9O_6$)<br>223.061 ($C_{11}H_{11}O_5$)	2b<br>Schmidt et al., 2010;<br>Baky et al., 2025
**98**	11.475	Kaempferol dihexoside (II)<br>(Kamp2Hex II)<br>$C_{27}H_{30}O_{16}$<br>Mw=610.1534	609.1465<br>-1.7 ppm	[M − H − 2Hex]$^{-}$<br>[M − H − Hex − H_2O]$^{-}$	285.039 ($C_{15}H_9O_6$)<br>429.082 ($C_{21}H_{17}O_{10}$)	2b<br>Lin and Harnly, 2009;<br>Schmidt et al., 2010;<br>Baky et al., 2025
**99**	11.596	Kaempferol-hydroxyferuloyl dihexoside<br>(Kamp-hFer2Hex)<br>$C_{37}H_{38}O_{20}$<br>Mw=802.19565	801.1890<br>-1.5 ppm	[M − H − h-Feruloyl]$^{-}$<br>[M − H − 2Hex − Kamp]$^{-}$<br>[M − H − 2Hex − h-Feruloyl]$^{-}$	609.146 ($C_{27}H_{29}O_{16}$)<br>191.034 ($C_{10}H_7O_4$)<br>285.040 ($C_{15}H_9O_6$)	2b<br>Lin and Harnly, 2009;<br>Olsen et al., 2009;<br>Schmidt et al., 2010;<br>Baky et al., 2025
**100**	11.896	Kaempferol-sinapoyl dihexoside<br>(Kamp-Sin2Hex)<br>$C_{38}H_{40}O_{20}$<br>Mw=816.2113	815.2045<br>-1.4 ppm	[M − H − Sinapoyl]$^{-}$<br>[M − H − Sinapoyl − 2Hex − H]$^{-}$<br>[M − H − Sinapoyl − H_2O]$^{-}$<br>[M − H − Sinapoyl − 2Hex]$^{-}$	609.146 ($C_{27}H_{29}O_{16}$)<br>284.033 ($C_{15}H_8O_6$)<br>591.135 ($C_{27}H_{27}O_{15}$)<br>285.039 ($C_{15}H_9O_6$)	2b<br>Lin and Harnly, 2009;<br>Olsen et al., 2009<br>Schmidt et al., 2010;<br>Baky et al., 2025
**101**	11.954	Kaempferol-sinapoyl-feruloyl trihexoside (Kamp-Sin-Fer3Hex)<br>$C_{54}H_{60}O_{29}$<br>Mw=1172.3220	1171.3148<br>-0.6 ppm	[M − H − Hex]$^{-}$<br>[M − H − Sinapoyl]$^{-}$<br>[M − H − Hex − H_2O]$^{-}$<br>[M − H − 3Hex − Sinapoyl − Feruloyl − H_2O]$^{-}$<br>[M − H − 2Hex − Sinapoyl − Feruloyl − H_2O]$^{-}$	1009.262 ($C_{48}H_{49}O_{24}$)<br>965.257 ($C_{43}H_{49}O_{25}$)<br>991.253 ($C_{48}H_{47}O_{23}$)<br>285.040 ($C_{15}H_9O_6$)<br>447.093 ($C_{21}H_{19}O_{11}$)	2b<br>Schmidt et al., 2010;<br>Baky et al., 2025
**102**	11.98	Quercetin glucuronide (QuerGluc)<br>$C_{21}H_{18}O_{13}$<br>Mw=478.0747	477.0674<br>-1.1 ppm	[M − H − HA]$^{-}$	301.035 ($C_{15}H_9O_7$)	2b
**103**	12.094	Kaempferol-feruloyl dihexoside<br>(Kamp-Fer2Hex)<br>$C_{37}H_{38}O_{19}$<br>Mw=786.2007	785.1939<br>-1.3 ppm	[M − H − Feruloyl]$^{-}$<br>[M − H − Feruloyl − H_2O]$^{-}$<br>[M − H − 2Hex − Feruloyl]$^{-}$	609.146 ($C_{27}H_{29}O_{16}$)<br>591.135 ($C_{27}H_{27}O_{15}$)<br>285.039 ($C_{15}H_9O_6$)	2b<br>Lin and Harnly, 2009;<br>Olsen et al., 2009;<br>Schmidt et al., 2010;<br>Baky et al., 2025
				**Anthocyanins**		
**104**	11.583	Cyanidin disinapoyl trihexoside<br>(Cy-2Sin3Hex)<br>$C_{55}H_{61}O_{29}^{+}$<br>Mw=1185.3299	1183.3150<br>-0.7 ppm	[M^{+} − 2H − Hex]$^{-}$<br>[M^{+} − 2H − Sinapoyl]$^{-}$<br>[M^{+} − 2H − Hex − Sinapoyl]$^{-}$	1021.262 ($C_{49}H_{49}O_{24}$)<br>977.257 ($C_{44}H_{49}O_{25}$)<br>815.204 ($C_{38}H_{39}O_{20}$)	2b<br>Olsen et al., 2010
**105**	11.59	Cyanidin feruloyl-sinapoyl trihexoside	1153.30445	[M^{+} − 2H − Hex]$^{-}$	991.251 ($C_{48}H_{47}O_{23}$)	2b

		(Cy-Fer-Sin3Hex)<br>$C_{54}H_{59}O_{28}^+$<br>Mw=1155.3193	-0.7 ppm	[M^+ − 2H − Sinapoyl]$^-$<br>[M^+ − 2H − Hex − Sinapoyl]$^-$	947.246 ($C_{43}H_{47}O_{24}$)<br>785.194 ($C_{37}H_{37}O_{19}$)	Olsen et al., 2010
				**Lignans**		
**106**	10.474	Lariciresinol dihexoside (Lar2Hex)<br>$C_{32}H_{44}O_{16}$<br>Mw=684.2629	683.2560<br>-1.4 ppm	[M − H − 2Hex − CH_2O]$^-$<br>[M − H − 2Hex]$^-$<br>[M − H − Hex]$^-$	329.139 ($C_{19}H_{21}O_5$)<br>359.150 ($C_{20}H_{23}O_6$)<br>521.203 ($C_{26}H_{33}O_{11}$)	2b<br>Galasso et al., 2014;<br>Baky et al., 2025;<br>Baky et al., 2025
**107**	10.727	Pinoresinol dihexoside (Pin2Hex)<br>$C_{32}H_{42}O_{16}$<br>Mw=682.2473	681.2408<br>-1.9 ppm	[M − H − 2Hex]$^-$<br>[M − H − Hex]$^-$<br>[M − H − 2Hex − $C_{14}H_{12}O$]$^-$<br>[M − H − 2Hex − $C_{12}H_{14}O_3$]$^-$	357.134 ($C_{20}H_{21}O_6$)<br>519.187 ($C_{26}H_{31}O_{11}$)<br>161.044 ($C_6H_9O_5$)<br>151.038 ($C_8H_7O_3$)	2b<br>Galasso et al., 2014
				**Complex hydroxycinnamic acid derivatives**		
**108**	10.946	*p*-Coumaroyl-sinapoyl trihexoside (I)<br>(Coum-Sin3Hex I)<br>$C_{38}H_{48}O_{22}$<br>Mw=856.2637	855.2568<br>-1.0 ppm	[M − H − Hex − Sinapoyl − H_2O]$^-$<br>[M − H − Hex]$^-$<br>[M − H − 3Hex − Coumaroyl − H_2O]$^-$<br>[M − H − 3Hex − Coumaroyl]$^-$<br>[M − H − 3Hex − Sinapoyl − H_2O]$^-$<br>[M − H − 3Hex − Sinapoyl]$^-$	469.135 ($C_{21}H_{25}O_{12}$)<br>693.204 ($C_{32}H_{37}O_{17}$)<br>205.050 ($C_{11}H_9O_4$)<br>223.061 ($C_{11}H_{11}O_5$)<br>145.028 ($C_9H_5O_2$)<br>163.039 ($C_9H_7O_3$)	2b
**109**	10.959	Feruloyl-sinapoyl trihexoside (Fer-Sin3Hex)<br>$C_{39}H_{50}O_{23}$<br>Mw=886.2743	885.2675<br>-1.2 ppm	[M − H − Hex]$^-$<br>[M − H − Hex − Sinapoyl − H_2O]$^-$<br>[M − H − 3Hex − Feruloyl − H_2O]$^-$<br>[M − H − 3Hex − Feruloyl]$^-$<br>[M − H − 3Hex − Sinapoyl]$^-$<br>[M − H − 3Hex − Sinapoyl − H_2O]$^-$	723.215 ($C_{33}H_{39}O_{18}$)<br>499.146 ($C_{22}H_{27}O_{13}$)<br>205.049($C_{11}H_9O_4$)<br>223.061 ($C_{11}H_{11}O_5$)<br>193.049 ($C_{10}H_9O_4$)<br>175.039 ($C_{10}H_7O_3$)	2b<br>Lin and Harnly, 2009;<br>Olsen et al., 2009;<br>Olsen et al., 2010
**110**	11.353	*p*-Coumaroyl-sinapoyl trihexoside (II)<br>(Coum-Sin3Hex II)<br>$C_{38}H_{48}O_{22}$<br>Mw=856.2637	855.2571<br>-1.4 ppm	[M − H − Hex − Sinapoyl ($C_{11}H_{10}O_4$) − H_2O]$^-$<br>[M − H − Hex]$^-$<br>[M − H − 3Hex − Coumaroyl − H_2O]$^-$<br>[M − H − 3Hex − Coumaroyl]$^-$	469.135 ($C_{21}H_{25}O_{12}$)<br>693.201 ($C_{32}H_{37}O_{17}$)<br>205.050 ($C_{11}H_9O_4$)<br>223.061 ($C_{11}H_{11}O_5$)	2b
**111**	11.564	Disinapoyl dihexoside (I) (2Sin2Hex I)<br>$C_{34}H_{42}O_{19}$<br>Mw=754.2320	753.2253<br>-1.5 ppm	[M − H − Sinapoyl − H_2O]$^-$<br>[M − H − 2Hex − Sinapoyl − H_2O]$^-$<br>[M − H − 2Hex − Sinapoyl]$^-$	529.157 ($C_{23}H_{29}O_{14}$)<br>205.050 ($C_{11}H_9O_4$)<br>223.061 ($C_{11}H_{11}O_5$)	2b<br>Lin and Harnly, 2009;<br>Olsen et al., 2009;<br>Olsen et al., 2010
**112**	11.665	Sinapoyl-feruloyl dihexoside (I)<br>(Sin-Fer2Hex I)<br>$C_{33}H_{40}O_{18}$<br>Mw=724.2215	723.2150<br>-1.9 ppm	[M − H − Feruloyl − H_2O]$^-$<br>[M − H − 2Hex − Feruloyl]$^-$<br>[M − H − 2Hex − Feruloyl − H_2O]$^-$<br>[M − H − 2Hex − Sinapoyl − H_2O]$^-$<br>[M − H − 2Hex − Sinapoyl]$^-$	529.157 ($C_{23}H_{29}O_{14}$)<br>223.061 ($C_{11}H_{11}O_5$)<br>205.050 ($C_{11}H_9O_4$)<br>175.039 ($C_{10}H_7O_3$)<br>193.049 ($C_{10}H_9O_4$)	2b<br>Lin and Harnly, 2009;<br>Olsen et al., 2009;<br>Olsen et al., 2010
**113**	11.894	Sinapoyl-hydroxyferuloyl dihexoside<br>(Sin-hFer2Hex)<br>$C_{33}H_{40}O_{19}$<br>Mw=740.2164	739.2096<br>-1.5 ppm	[M − H − Sinapoyl − H_2O]$^-$<br>[M − H − 2Hex − Sinapoyl − H_2O]$^-$<br>[M − H− 2Hex − h-Feruloyl]$^-$	515.141 ($C_{22}H_{27}O_{14}$)<br>191.034 ($C_{10}H_7O_4$)<br>223.061 ($C_{11}H_{11}O_5$)	2b
**114**	12.057	Sinapoyl-feruloyl-dihydroferuloyl dihexoside (Sin-Fer-dhFer2Hex)<br>$C_{43}H_{52}O_{22}$<br>Mw=920.2950	919.2881<br>-1.1 ppm	M − H − Feruloyl − dh-Feruloyl − 2Hex − $2H_2O$]$^-$<br>[M − H − Sinapoyl − dh-Feruloyl − $2H_2O$]$^-$<br>[M − H − dh-Feruloyl − H_2O]$^-$<br>[M − H − Feruloyl − dh-Feruloyl − Sinapoyl − Hex − H_2O]$^-$<br>[M − H − Feruloyl − dh-Feruloyl − 2Hex − H_2O]$^-$<br>[M − H − Sinapoyl − H_2O]$^-$	205.050 ($C_{11}H_9O_4$)<br>499.146 ($C_{22}H_{27}O_{13}$)<br>723.215 ($C_{33}H_{39}O_{18}$)<br>179.055 ($C_6H_{11}O_6$)<br>223.061 ($C_{11}H_{11}O_5$)<br>695.219 ($C_{32}H_{39}O_{17}$)	3
**115**	12.147	Disinapoyl dihexoside (II) (2Sin2Hex II)<br>$C_{34}H_{42}O_{19}$<br>Mw=754.2320	753.2254<br>-1.6 ppm	[M − H − Hex − Sinapoyl − $C_4H_8O_4$ − H_2O]$^-$<br>[M − H − 2Hex − Sinapoyl]$^-$<br>[M − H − 2Hex − Sinapoyl − H_2O]$^-$<br>[M − H − Sinapoyl − $C_4H_8O_4$]$^-$<br>[M − H − Hex − Sinapoyl − $C_4H_8O_4$]$^-$	247.061 ($C_{13}H_{11}O_5$)<br>223.061 ($C_{11}H_{11}O_5$)<br>205.050 ($C_{11}H_9O_4$)<br>427.125 ($C_{19}H_{23}O_{11}$)<br>265.072($C_{13}H_{13}O_6$)	2b<br>Lin and Harnly, 2009;<br>Olsen et al., 2009;<br>Olsen et al., 2010
**116**	12.167	Diferuloyl-dihydroferuloyl dihexoside<br>(2Fer-dhFer2Hex)<br>$C_{42}H_{50}O_{21}$<br>Mw=890.2845	889.2776<br>-1.1 ppm	[M − H − Feruloyl − dh-Feruloyl − $2H_2O$]$^-$<br>[M − H − 2Hex − dh-Feruloyl − Feruloyl − $2H_2O$]$^-$<br>[M − H − 2Hex − dh-Feruloyl − Feruloyl − H_2O]$^-$<br>[M − H − dh-Feruloyl − H_2O]$^-$	499.146 ($C_{22}H_{27}O_{13}$)<br>175.039 ($C_{10}H_7O_3$)<br>193.049 ($C_{10}H_9O_4$)<br>693.204 ($C_{32}H_{37}O_{17}$)	3
**117**	12.194	Sinapoyl-feruloyl dihexoside (II)<br>(Sin-Fer2Hex II)<br>$C_{33}H_{40}O_{18}$<br>Mw=724.2215	723.2150<br>-1.9 ppm	[M − H − Hex − Sinapoyl − $C_4H_8O_4$ − H_2O]$^-$<br>[M − H − 2Hex − Feruloyl]$^-$<br>[M − H − Feruloyl − $C_4H_8O_4$]$^-$<br>[M − H − 2Hex − Feruloyl − H_2O]$^-$<br>[M − H − Hex − Feruloyl − $C_4H_8O_4$ − H_2O]$^-$	217.050 ($C_{12}H_9O_4$)<br>223.061 ($C_{11}H_{11}O_5$)<br>427.125 ($C_{19}H_{23}O_{11}$)<br>205.050 ($C_{11}H_9O_4$)<br>247.061($C_{13}H_{11}O_5$)	2b<br>Lin and Harnly, 2009;<br>Olsen et al., 2009;<br>Olsen et al., 2010

**118**	12.336	Disinapoyl dihexoside (III)<br>(2Sin2Hex III)<br>$C_{34}H_{42}O_{19}$<br>Mw=754.2320	753.2252<br>-1.3 ppm	[M − H − 2Hex − Sinapoyl − H_2O]$^-$<br>[M − H − Sinapoyl − H_2O]$^-$<br>[M − H − 2Hex − Sinapoyl]$^-$<br>[M − H − Hex − Sinapoyl − $C_4H_8O_4$ − H_2O]$^-$	205.050 ($C_{11}H_9O_4$)<br>529.156 ($C_{23}H_{29}O_{14}$)<br>223.061 ($C_{11}H_{11}O_5$)<br>247.061 ($C_{13}H_{11}O_5$)	2b<br>Lin and Harnly, 2009;<br>Olsen et al., 2009;<br>Olsen et al., 2010
**119**	12.432	Sinapoyl-feruloyl dihexoside (III)<br>(Sin-Fer2Hex III)<br>$C_{33}H_{40}O_{18}$<br>Mw=724.2215	723.2147<br>-1.6 ppm	[M − H − Sinapoyl − H_2O]$^-$<br>[M − H − 2Hex − Sinapoyl − H_2O]$^-$<br>[M − H − 2Hex − Sinapoyl]$^-$<br>[M − H − 2Hex − Feruloyl]$^-$<br>[M − H − 2Hex − Feruloyl − H_2O]$^-$	499.146 ($C_{22}H_{27}O_{13}$)<br>175.039 ($C_{10}H_7O_3$)<br>193.049 ($C_{10}H_9O_4$)<br>223.061 ($C_{11}H_{11}O_5$)<br>205.050 ($C_{11}H_9O_4$)	2b<br>Lin and Harnly, 2009;<br>Olsen et al., 2009;<br>Olsen et al., 2010
**120**	12.507	Diferuloyl dihexoside (I) (2Fer2Hex I)<br>$C_{32}H_{38}O_{17}$<br>Mw=694.2109	693.2041<br>-1.6 ppm	[M − H − 2Hex − Feruloyl − H_2O]$^-$<br>[M − H − 2Hex − Feruloyl]$^-$<br>[M − H − Feruloyl − H_2O]$^-$	175.039 ($C_{10}H_7O_3$)<br>193.049 ($C_{10}H_9O_4$)<br>499.146 ($C_{22}H_{27}O_{13}$)	2b
**121**	12.658	Disinapoyl dihexoside (IV) (2Sin2Hex IV)<br>$C_{34}H_{42}O_{19}$<br>Mw=754.2320	753.2252<br>-1.4 ppm	[M − H − 2Hex − Sinapoyl − H_2O]$^-$<br>[M − H − Sinapoyl − H_2O]$^-$<br>[M − H − 2Hex − Sinapoyl]$^-$<br>[M − H − Hex − Sinapoyl − $C_4H_8O_4$ − H_2O]$^-$	205.050 ($C_{11}H_9O_4$)<br>529.156 ($C_{23}H_{29}O_{14}$)<br>223.061 ($C_{11}H_{11}O_5$)<br>247.061 ($C_{13}H_{11}O_5$)	2b<br>Lin and Harnly, 2009;<br>Olsen et al., 2009;<br>Olsen et al., 2010
**122**	12.714	Sinapoyl-feruloyl dihexoside (IV)<br>(Sin-Fer2Hex IV)<br>$C_{33}H_{40}O_{18}$<br>Mw=724.2215	723.2148<br>-1.6 ppm	[M − H − Sinapoyl − H_2O]$^-$<br>[M − H − 2Hex − Sinapoyl]$^-$<br>[M − H − 2Hex − Sinapoyl − H_2O]$^-$<br>[M − H − 2Hex − Feruloyl − H_2O]$^-$<br>[M − H − 2Hex − Feruloyl]$^-$	499.146 ($C_{22}H_{27}O_{13}$)<br>193.049 ($C_{10}H_9O_4$)<br>175.039 ($C_{10}H_7O_3$)<br>205.050 ($C_{11}H_9O_4$)<br>223.061 ($C_{11}H_{11}O_5$)	2b<br>Lin and Harnly, 2009;<br>Olsen et al., 2009;<br>Olsen et al., 2010
**123**	12.771	Diferuloyl dihexoside (II) (2Fer2Hex II)<br>$C_{32}H_{38}O_{17}$<br>Mw=694.2109	693.2042<br>-1.6 ppm	[M − H − 2Hex − Feruloyl]$^-$<br>[M − H − 2Hex − Feruloyl − H_2O]$^-$<br>[M − H − Feruloyl − H_2O]$^-$	193.049 ($C_{10}H_9O_4$)<br>175.039 ($C_{10}H_7O_3$)<br>499.146 ($C_{22}H_{27}O_{13}$)	2b
**124**	13.021	Sinapoyl-sinapaldehyde dihexoside<br>(Sin-SinAld2Hex)<br>$C_{34}H_{42}O_{18}$<br>Mw=738.2371	737.2305<br>-1.7 ppm	[M − H − 2Hex − SinapAld − H_2O]$^-$<br>[M − H − 2Hex − SinapAld]$^-$<br>[M − H − Hex − Sinapoyl − SinapAld]$^-$	205.050 ($C_{11}H_9O_4$)<br>223.061 ($C_{11}H_{11}O_5$)<br>179.055 ($C_6H_{11}O_6$)	3
**125**	13.099	Trisinapoyl dihexoside (I) (3Sin2Hex I)<br>$C_{45}H_{52}O_{23}$<br>Mw=960.2899	959.2830<br>-1.0 ppm	[M − H − Sinapoyl − H_2O]$^-$<br>[M − H − 2Hex − 2Sinapoyl − H_2O]$^-$<br>[M − H − 2Sinapoyl − $2H_2O$]$^-$<br>[M − H − 2Sinapoyl − H_2O]$^-$<br>[M − H − 2Hex − 2Sinapoyl]$^-$	735.214 ($C_{34}H_{39}O_{18}$)<br>205.049 ($C_{11}H_9O_4$)<br>511.145 ($C_{23}H_{27}O_{13}$)<br>529.156 ($C_{23}H_{29}O_{14}$)<br>223.061 ($C_{11}H_{11}O_5$)	2b<br>Lin and Harnly, 2009;<br>Olsen et al., 2009;<br>Olsen et al., 2010
**126**	13.204	Disinapoyl-feruloyl dihexoside (I)<br>(2Sin-Fer2Hex I)<br>$C_{44}H_{50}O_{22}$<br>Mw=930.2794	929.2727<br>-1.2 ppm	[M − H − Sinapoyl − H_2O]$^-$<br>[M − H − 2Hex − Sinapoyl − Feruloyl − H_2O]$^-$<br>[M − H − 2Hex − 2Sinapoyl − H_2O]$^-$<br>[M − H − Sinapoyl − Feruloyl − H_2O]$^-$<br>[M − H − Sinapoyl − Feruloyl − $2H_2O$]$^-$<br>[M − H − 2Sinapoyl − H_2O]$^-$	705.204 ($C_{33}H_{37}O_{17}$)<br>205.049 ($C_{11}H_9O_4$)<br>175.039 ($C_{10}H_7O_3$)<br>529.155 ($C_{23}H_{29}O_{14}$)<br>511.145 ($C_{23}H_{27}O_{13}$)<br>499.147 ($C_{22}H_{27}O_{13}$)	2b<br>Lin and Harnly, 2009;<br>Olsen et al., 2009;<br>Olsen et al., 2010
**127**	13.321	Sinapoyl-diferuloyl dihexoside (I)<br>(Sin-2Fer2Hex I)<br>$C_{43}H_{48}O_{21}$<br>Mw=900.2688	899.2622<br>-1.3 ppm	[M − H − Feruloyl − H_2O]$^-$<br>[M − H − Sinapoyl − H_2O]$^-$<br>[M − H − 2Hex − Sinapoyl − Feruloyl − H_2O]$^-$<br>[M − H − Sinapoyl − Feruloyl − H_2O]$^-$<br>[M − H − Sinapoyl − Feruloyl − $2H_2O$]$^-$<br>[M − H − 2Feruloyl − H_2O]$^-$	705.204 ($C_{33}H_{37}O_{17}$)<br>675.194 ($C_{32}H_{35}O_{16}$)<br>175.039 ($C_{10}H_7O_3$)<br>499.145 ($C_{22}H_{27}O_{13}$)<br>481.135 ($C_{22}H_{25}O_{12}$)<br>529.156 ($C_{23}H_{29}O_{14}$)	2b
**128**	13.405	Disinapoyl hexoside (2SinHex)<br>$C_{28}H_{32}O_{14}$<br>Mw=592.1792	591.1725<br>-1.9 ppm	[M − H − Hex − Sinapoyl]$^-$<br>[M − H − Hex − Sinapoyl − H_2O]$^-$<br>[M − H − Sinapoyl − H_2O]$^-$<br>[M − H − Sinapoyl − CH_3 − H_2O]$^-$	223.061 ($C_{11}H_{11}O_5$)<br>205.050 ($C_{11}H_9O_4$)<br>367.104 ($C_{17}H_{19}O_9$)<br>352.080 ($C_{16}H_{16}O_9$)	2b
**129**	13.513	Sinapoyl-feruloyl hexoside (Sin-FerHex)<br>$C_{27}H_{30}O_{13}$<br>Mw=562.1686	561.1617<br>-1.6 ppm	[M − H − Hex − Feruloyl]$^-$<br>[M − H − Hex − Sinapoyl − H_2O]$^-$<br>[M − H − Sinapoyl − H_2O]$^-$<br>[M − H − Hex − Sinapoyl]$^-$	223.061 ($C_{11}H_{11}O_5$)<br>175.039 ($C_{10}H_7O_3$)<br>337.093 ($C_{16}H_{17}O_8$)<br>193.049 ($C_{10}H_9O_4$)	2b
**130**	13.514	Trisinapoyl dihexoside (II) (3Sin2Hex II)<br>$C_{45}H_{52}O_{23}$<br>Mw=960.2899	959.2832<br>-1.2 ppm	[M − H − Sinapoyl − H_2O]$^-$<br>[M − H − 2Sinapoyl − $2H_2O$]$^-$<br>[M − H − 2Hex − 2Sinapoyl − H_2O]$^-$<br>[M − H − 2Hex − 2Sinapoyl]$^-$<br>[M − H − 2Sinapoyl − H_2O]$^-$	735.215 ($C_{34}H_{39}O_{18}$)<br>511.146 ($C_{23}H_{27}O_{13}$)<br>205.050 ($C_{11}H_9O_4$)<br>223.061 ($C_{11}H_{11}O_5$)<br>529.156 ($C_{23}H_{29}O_{14}$)	2b<br>Lin and Harnly, 2009;<br>Olsen et al., 2009;<br>Olsen et al., 2010
**131**	13.595	Disinapoyl-feruloyl dihexoside (II)<br>(2Sin-Fer2Hex II)<br>$C_{44}H_{50}O_{22}$<br>Mw=930.2794	929.2728<br>-1.4 ppm	[M − H − Sinapoyl − H_2O]$^-$<br>[M − H − Sinapoyl − Feruloyl − $2H_2O$]$^-$<br>[M − H − 2Hex − Sinapoyl − Feruloyl − H_2O]$^-$<br>[M − H − 2Hex − 2Sinapoyl − H_2O]$^-$	705.205 ($C_{33}H_{37}O_{17}$)<br>511.146 ($C_{23}H_{27}O_{13}$)<br>205.051 ($C_{11}H_9O_4$)<br>175.039 ($C_{10}H_7O_3$)	2b<br>Lin and Harnly, 2009;<br>Olsen et al., 2009;<br>Olsen et al., 2010

				$[M - H - 2Hex - Sinapoyl - Feruloyl]^-$	223.061 ($C_{11}H_{11}O_5$)	
**132**	13.623	Diferuloyl hexoside (2FerHex)<br>$C_{26}H_{28}O_{12}$<br>Mw=532.1581	531.1510<br>-1.4 ppm	$[M - H - Hex - Feruloyl]^-$<br>$[M - H - Hex - Feruloyl - H_2O]^-$<br>$[M - H - Feruloyl - H_2O]^-$	193.049 ($C_{10}H_9O_4$)<br>175.039 ($C_{10}H_7O_3$)<br>337.093 ($C_{16}H_{17}O_8$)	2b
**133**	13.699	Sinapoyl-diferuloyl dihexoside (II)<br>(Sin-2Fer2Hex II)<br>$C_{43}H_{48}O_{21}$<br>Mw=900.2688	899.2622<br>-1.3 ppm	$[M - H - Feruloyl - H_2O]^-$<br>$[M - H - 2Feruloyl - 2H_2O]^-$<br>$[M - H - Sinapoyl - Feruloyl - 2H_2O]^-$<br>$[M - H - Sinapoyl - H_2O]^-$<br>$[M - H - 2Hex - Sinapoyl - Feruloyl - H_2O]^-$	705.204 ($C_{33}H_{37}O_{17}$)<br>511.145 ($C_{23}H_{27}O_{13}$)<br>481.135 ($C_{22}H_{25}O_{12}$)<br>675.194 ($C_{32}H_{35}O_{16}$)<br>175.039 ($C_{10}H_7O_3$)	2b
**134**	13.975	Trisinapoyl dihexoside (III)<br>(3Sin2Hex III)<br>$C_{45}H_{52}O_{23}$<br>Mw=960.2899	959.2831<br>-1.0 ppm	$[M - H - Sinapoyl - H_2O]^-$<br>$[M - H - 2Hex - 2Sinapoyl - H_2O]^-$<br>$[M - H - 2Sinapoyl - 2H_2O]^-$<br>$[M - H - 2Sinapoyl - H_2O]^-$<br>$[M - H - 2Hex - 2Sinapoyl]^-$	735.215 ($C_{34}H_{39}O_{18}$)<br>205.050 ($C_{11}H_9O_4$)<br>511.146 ($C_{23}H_{27}O_{13}$)<br>529.157 ($C_{23}H_{29}O_{14}$)<br>223.061 ($C_{11}H_{11}O_5$)	2b<br>Lin and Harnly, 2009;<br>Olsen et al., 2009;<br>Olsen et al., 2010
**135**	14.07	Disinapoyl-feruloyl dihexoside (III)<br>(2Sin-Fer2Hex III)<br>$C_{44}H_{50}O_{22}$<br>Mw=930.2794	929.2727<br>-1.3 ppm	$[M - H - Sinapoyl - H_2O]^-$<br>$[M - H - 2Hex - Sinapoyl - Feruloyl - H_2O]^-$<br>$[M - H - 2Hex - 2Sinapoyl - H_2O]^-$<br>$[M - H - Sinapoyl - Feruloyl - 2H_2O]^-$	705.204 ($C_{33}H_{37}O_{17}$)<br>205.050 ($C_{11}H_9O_4$)<br>175.039 ($C_{10}H_7O_3$)<br>511.145 ($C_{23}H_{27}O_{13}$)	2b<br>Lin and Harnly, 2009;<br>Olsen et al., 2009;<br>Olsen et al., 2010

Abbreviations: Caffeoyl, $C_9H_6O_3^-$ (*m/z* 162.031); CQ (caffeoylquinoyl), $C_{16}H_{16}O_8^-$ (*m/z* 336.084); Coumaroyl, $C_9H_6O_2^-$ (*m/z* 146.037); Feruloyl, $C_{10}H_8O_3^-$ (*m/z* 176.047); dh-Feruloyl (dihydroferuloyl), $C_{10}H_{10}O_3^-$ (*m/z* 178.063); h-Feruloyl (hydroxyferuloyl), $C_{10}H_8O_4^-$ (*m/z* 192.042); HA (hexuronic acid), $C_6H_8O_6^-$ (*m/z* 176.032); Hex (hexose), $C_6H_{10}O_5^-$ (*m/z* 162.053); Kamp (kaempferol), $C_{15}H_{10}O_6^-$ (*m/z* 286.048); Pent (pentose), $C_5H_8O_4^-$ (*m/z* 132.042); SinapAld (sinapoyl aldehyde), $C_{11}H_{10}O_3^-$ (*m/z* 190.063); Sinapoyl, $C_{11}H_{10}O_4^-$ (*m/z* 206.058); Syringoyl, $C_9H_8O_4^-$ (*m/z* 180.042); Quer (quercetin), $C_{15}H_{10}O_7^-$ (*m/z* 302.043); Quinoyl, $C_7H_{10}O_5^-$ (*m/z* 174.052).

Identification confidence levels were assigned according to Schymanski et al. (2014): Level 1, structure confirmed by comparison with an authentic reference standard analysed under identical conditions, based on agreement in retention time, accurate mass, MS/MS fragmentation, and UV–Vis spectrum; Level 2b, probable structure supported by diagnostic HRMS/MS evidence; Level 3, tentative structural candidate for which the available MS/MS evidence does not allow an unambiguous structural assignment. Literature references are provided where direct or closely related structural and/or fragmentation evidence was available.

Table S2. Variance partitioning of multivariate phenolic profiles based on the PERMANOVA model for green- and red-leaved kale.

Cultivar	Source	df	Sum of squares	R^2
Green	Processing	2	2162.8	0.61618
	Iodine	2	326.7	0.09308
	Processing × Iodine	4	849.7	0.24208
	Residual	18	170.8	0.04866
Red	Processing	2	2404.0	0.68489
	Iodine	2	253.8	0.07230
	Processing × Iodine	4	689.4	0.19642
	Residual	18	162.8	0.04640

The analysis was performed separately for each cultivar using the complete 3 × 3 factorial subset comprising three processing treatments (blanching, boiling, and steaming) and three iodine treatments (Control, KIO_3, and DIQ). Raw samples were excluded because they were available only for untreated controls. Euclidean distances were calculated from log-transformed and autoscaled LC–HRMS signal intensities. Because the three processing replicates within each cultivar × iodine × processing combination were derived from the same pooled plant material and therefore did not represent independent biological replicates of the iodine treatment, R^2 values are reported as descriptive measures of variance partitioning within the present dataset. Permutation-based significance tests were not used for biological inference regarding iodine treatment or its interaction with processing.

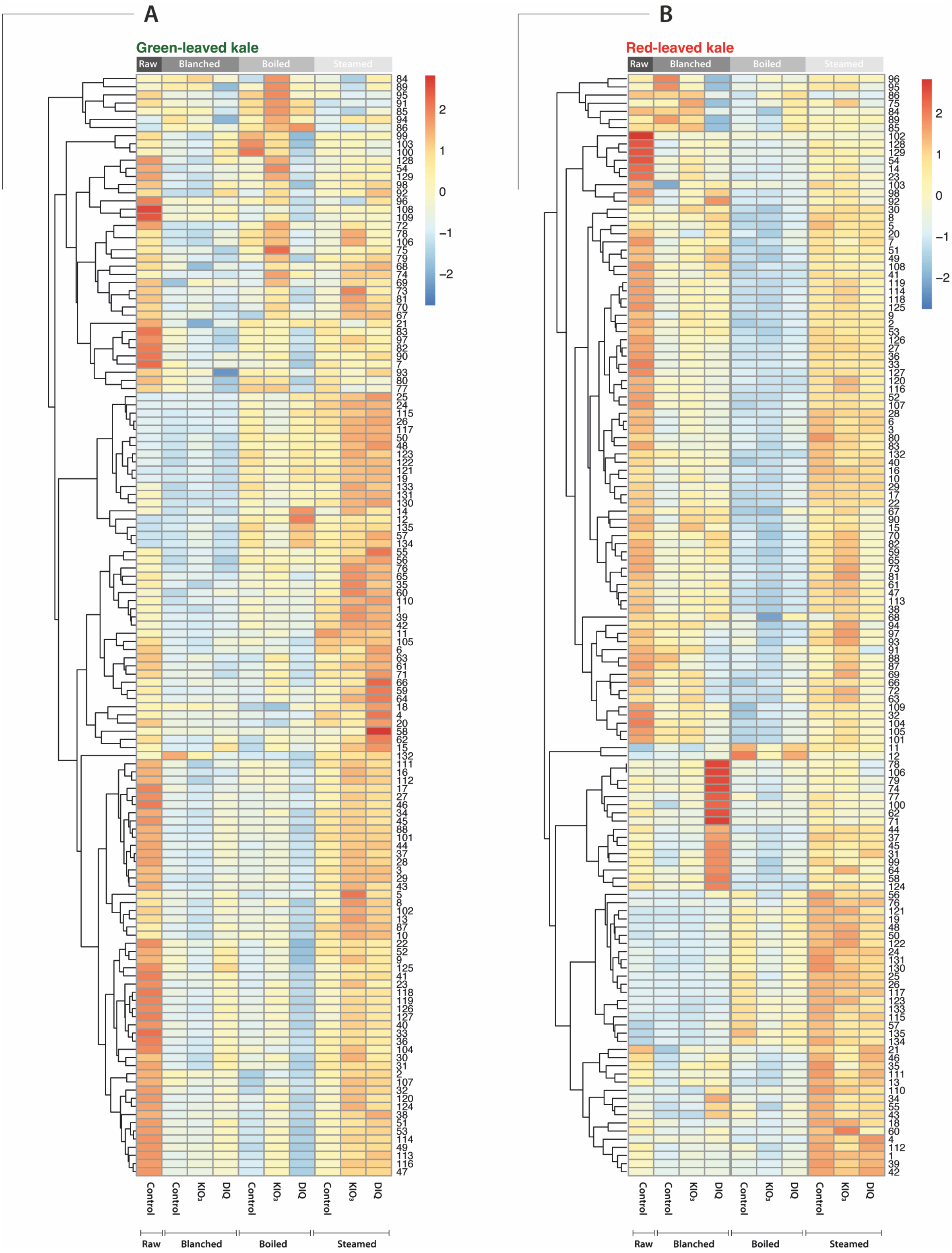

Figure S1. Hierarchical clustering heat maps of phenolic profiles in green- and red-leaved kale cultivars subjected to iodine biofortification and thermal processing. Heat maps show row-wise standardized (z-score) mean LC–HRMS signal intensities of 135 annotated phenolic compounds in (A) green- and (B) red-leaved kale. Compounds (rows) were ordered by hierarchical clustering using Euclidean distance and complete linkage, whereas treatment conditions (columns) were retained in a predefined experimental order to facilitate comparison across processing and iodine treatments. Compound numbers correspond to those reported in Table 1 and Table S1.

Table S3. Relative summed LC–HRMS signal intensities of the major phenolic classes in green- and red-leaved kale expressed relative to the corresponding raw control (Raw Control = 100%). Values are presented as mean ± SD (n = 3 biological replicates).

Major phenolic class	Raw	Blanched			Boiled			Steamed		
	Ctrl	Ctrl	KIO_3	DIQ	Ctrl	KIO_3	DIQ	Ctrl	KIO_3	DIQ
**Green-leaved kale**										
Phenolic acids and their glycosides	100	62.87 ±0.53	56.71 ±1.25	66.53 ±1.81	70.3 ±0.47	73.48 ±1.02	60.42 ±4.40	95.47 ±4.90	110.54 ±2.91	108.09 ±3.65
Hydroxycinnamoylquinic acids	100	48.67 ±0.27	42.94 ±0.30	64.62 ±1.05	54.52 ±0.98	63.17 ±0.72	39.43 ±0.13	81.3 ±1.19	95.74 ±2.83	86.72 ±2.25
Flavonols	100	70.74 ±0.19	68.32 ±0.50	64.16 ±0.11	79.74 ±1.65	93.25 ±0.35	64.58 ±0.87	83.44 ±0.75	92.62 ±1.53	92.70 ±0.40
Complex hydroxycinnamic acid derivatives	100	56.79 ±1.46	54.70 ±0.81	68.80 ±1.76	62.40 ±1.80	72.38 ±1.48	51.44 ±0.80	73.99 ±1.17	90.98 ±3.26	89.11 ±1.93
**Red-leaved kale**										
Phenolic acids and their glycosides	100	51.78 ±1.58	79.53 ±1.86	65.06 ±3.47	49.56 ±5.77	39.43 ±2.05	55.91 ±0.27	102.63 ±7.03	103.45 ±2.06	95.54 ±3.31
Hydroxycinnamoylquinic acids	100	54.64 ±1.70	77.23 ±6.65	87.00 ±6.91	45.12 ±4.73	36.27 ±1.47	51.81 ±1.82	113.92 ±5.38	106.35 ±6.07	107.99 ±4.72
Flavonols	100	82.91 ±1.44	87.54 ±5.20	72.36 ±3.83	61.91 ±5.08	55.02 ±1.02	72.67 ±1.69	89.61 ±4.02	97.32 ±2.36	76.97 ±0.66
Complex hydroxycinnamic acid derivatives	100	56.15 ±1.60	69.41 ±4.02	67.04 ±3.36	46.36 ±4.13	38.69 ±0.06	47.51 ±1.27	91.86 ±3.55	96.09 ±1.10	81.16 ±0.95

Table S4. Compound-level differences between steamed and boiled kale phenolic profiles. Steamed-versus-boiled contrasts were estimated separately for green- and red-leaved kale within each of the three pooled source materials corresponding to the Control, KIO_3, and DIQ treatments and then averaged across these pools to obtain an overall processing contrast. Iodine treatment was treated only as a source-pool stratification factor, and no inferential conclusions regarding iodine effects or Processing × Iodine interactions were drawn from these models. Positive $\log_2FC$ values indicate higher relative LC–HRMS signal intensities in steamed samples, whereas negative values indicate higher signals in boiled samples. P-values were adjusted for multiple testing within each cultivar using the Benjamini–Hochberg procedure. The combined threshold was defined as BH-adjusted $p < 0.05$ and $|\log_2FC| \geq 0.5$.

Compound No.	Compound	log2FC (Steamed/Boiled)	p-value	BH-adjusted p-value	Combined threshold
**Green-leaved kale**					
1	SyrQA	0.912	2.88E-14	6.48E-14	Yes
2	CafA2Hex	1.127	2.59E-22	8.44E-21	Yes
3	PrcAHex	0.951	3.28E-20	2.60E-19	Yes
4	CoumA2Hex I	1.609	2.03E-08	2.91E-08	Yes
5	CoumA2Hex II	1.135	7.97E-08	1.11E-07	Yes
6	GAHex	0.924	1.32E-19	8.32E-19	Yes
7	SinA2Hex I	0.566	3.50E-14	7.75E-14	Yes
8	CoumA2Hex III	0.988	7.17E-16	2.10E-15	Yes
9	FerA2Hex I	1.031	9.02E-08	1.24E-07	Yes
10	SinA2Hex II	0.776	5.33E-07	6.99E-07	Yes
11	CafAHex I	0.996	4.21E-06	5.31E-06	Yes
12	hFerAHex I	-0.172	3.54E-03	4.05E-03	No
13	SinA2Pent	0.732	2.63E-17	9.59E-17	Yes
14	CafAHex II	-0.011	9.64E-01	9.64E-01	No
15	hFerAHex II	0.478	1.47E-10	2.42E-10	No
16	FerA2Hex II	0.451	2.30E-17	8.62E-17	No
17	SinA2Hex III	0.536	1.14E-16	3.83E-16	Yes
18	FerAHex I	1.060	2.26E-12	4.30E-12	Yes
19	SinA2Hex IV	0.361	2.33E-12	4.37E-12	No

20	CoumAHex	0.903	1.66E-23	1.35E-21	Yes
21	SinAHex I	-0.051	1.47E-02	1.62E-02	No
22	SinAHex II	0.669	1.04E-07	1.42E-07	Yes
23	FerAHex II	0.875	1.22E-19	8.23E-19	Yes
24	CafA	1.158	1.99E-20	1.80E-19	Yes
25	hFerA	1.151	3.08E-19	1.43E-18	Yes
26	SinA	0.917	1.06E-18	4.62E-18	Yes
27	CafQAHex I	1.000	1.42E-19	8.32E-19	Yes
28	CoumQAHex	1.059	2.23E-18	9.12E-18	Yes
29	CafQAHex II	0.713	2.53E-19	1.22E-18	Yes
30	CafQA I	0.696	1.99E-21	2.98E-20	Yes
31	FerQAHex	0.845	1.42E-16	4.67E-16	Yes
32	Sin-CafQAPentHex	0.410	1.23E-06	1.58E-06	No
33	CoumQA I	0.862	4.87E-22	1.10E-20	Yes
34	CafQA II	0.554	5.32E-18	2.05E-17	Yes
35	Sin-CafQAHex I	0.604	5.59E-06	6.99E-06	Yes
36	FerQA I	0.852	3.27E-21	4.42E-20	Yes
37	CafQA III	1.066	2.31E-19	1.18E-18	Yes
38	hFer-CafQAHex	1.146	3.13E-22	8.44E-21	Yes
39	CoumQA II	1.021	1.32E-21	2.24E-20	Yes
40	2Sin-CoumQA2Hex	0.787	3.96E-17	1.41E-16	Yes
41	Sin-2CoumQAHex	0.961	1.84E-18	7.77E-18	Yes
42	FerQA II	0.889	3.88E-20	2.91E-19	Yes
43	CoumQA III	1.074	2.00E-23	1.35E-21	Yes
44	2Sin-CafQA2Hex	1.985	1.66E-22	7.45E-21	Yes
45	FerQA III	0.893	7.75E-22	1.49E-20	Yes
46	SyringAld-SinQAHex	1.714	6.11E-19	2.75E-18	Yes
47	Fer-CafQAHex I	0.709	2.35E-19	1.18E-18	Yes
48	Sin-CafQAHex II	0.529	2.80E-10	4.44E-10	Yes
49	Sin-hFer-CafQAHex	0.925	2.23E-20	1.88E-19	Yes
50	Fer-CafQAHex II	0.347	1.44E-09	2.24E-09	No
51	2Sin-CafQAHex I	0.931	5.50E-21	5.74E-20	Yes
52	dhFer-2Sin-CoumQAHex	0.818	7.72E-09	1.14E-08	Yes
53	Fer-Sin-CafQAHex I	0.880	4.05E-21	4.97E-20	Yes
54	Fer-CafQA	-0.007	8.59E-01	8.65E-01	No
55	2Sin-CafQAHex II	0.420	8.29E-12	1.42E-11	No
56	Fer-Sin-CafQAHex II	0.276	2.54E-09	3.85E-09	No
57	2Sin-CafQAHex III	0.278	2.30E-06	2.94E-06	No
58	Quer4Hex	1.014	6.15E-03	6.98E-03	Yes
59	Quer3Hex	0.490	4.20E-16	1.26E-15	No
60	QuerRham3Hex	0.581	3.84E-15	9.97E-15	Yes
61	Kamp3Hex I	0.817	4.28E-18	1.70E-17	Yes
62	Irham3Hex	0.635	2.66E-15	7.05E-15	Yes
63	Kamp-hFer5Hex	0.429	9.57E-11	1.59E-10	No
64	Quer-hFer4Hex	0.949	6.67E-21	6.43E-20	Yes
65	Quer-Caf4Hex	0.532	1.24E-08	1.83E-08	Yes
66	Kamp-Caf5Hex	0.606	4.21E-05	5.16E-05	Yes
67	Quer-Sin5Hex	0.259	1.51E-08	2.19E-08	No
68	Erio2Hex	0.279	6.02E-01	6.20E-01	No

69	Kamp4Hex	0.120	5.81E-01	6.03E-01	No
70	Kamp-hFer4Hex	0.222	2.43E-12	4.50E-12	No
71	Quer-hFer3Hex	0.571	5.00E-17	1.73E-16	Yes
72	Kamp-Sin5Hex	-0.061	2.73E-03	3.15E-03	No
73	Kamp-Caf4Hex	0.382	5.64E-14	1.23E-13	No
74	Quer-Sin4Hex	0.032	4.89E-02	5.24E-02	No
75	Kamp-Fer5Hex	-0.076	8.73E-04	1.02E-03	No
76	Kamp3Hex II	0.396	3.36E-12	6.04E-12	No
77	Kamp-hFer3Hex	-0.074	1.62E-01	1.71E-01	No
78	Kamp-Sin4Hex	0.009	6.27E-01	6.36E-01	No
79	Quer-Sin3Hex	0.193	2.25E-08	3.20E-08	No
80	Kamp-Caf3Hex	0.106	4.77E-04	5.60E-04	No
81	Kamp-Fer4Hex	0.337	1.26E-12	2.47E-12	No
82	Kamp-Sin3Hex	0.458	8.72E-15	2.18E-14	No
83	Kamp-Fer3Hex	0.343	2.09E-15	5.66E-15	No
84	Kamp-2hFer5Hex	-0.163	3.59E-02	3.87E-02	No
85	Kamp-hFer-Sin5Hex	-0.514	2.02E-13	4.19E-13	Yes
86	Quer-2Sin5Hex	-0.734	2.22E-07	2.97E-07	Yes
87	Kamp-Sin3Hex-h	0.697	2.55E-12	4.64E-12	Yes
88	Kamp-Fer3Hex-h	0.700	9.76E-14	2.09E-13	Yes
89	Kamp-Fer-hFer5Hex	-0.184	4.10E-04	4.85E-04	No
90	Kamp2Hex I	0.242	4.07E-10	6.40E-10	No
91	Kamp-2Sin5Hex	-0.409	1.31E-12	2.52E-12	No
92	Quer2Hex	0.275	5.65E-05	6.87E-05	No
93	Kamp-Sin-hFer4Hex	0.110	3.22E-02	3.51E-02	No
94	Quer-2Sin4Hex	-0.080	1.94E-02	2.13E-02	No
95	Kamp-Sin-Fer5Hex	-0.247	8.22E-05	9.82E-05	No
96	Kamp-2Fer5Hex	-0.146	4.94E-01	5.17E-01	No
97	Kamp-2Sin4Hex	0.261	1.58E-10	2.56E-10	No
98	Kamp2Hex II	0.350	4.59E-13	9.24E-13	No
99	Kamp-hFer2Hex	0.134	4.63E-07	6.13E-07	No
100	Kamp-Sin2Hex	-0.215	1.28E-07	1.73E-07	No
101	Kamp-Sin-Fer3Hex	0.697	9.21E-15	2.26E-14	Yes
102	QuerGluc	0.760	1.66E-15	4.67E-15	Yes
103	Kamp-Fer2Hex	-0.033	1.01E-01	1.08E-01	No
104	Cy-2Sin3Hex	0.440	8.37E-06	1.04E-05	No
105	Cy-Fer-Sin3Hex	0.390	6.35E-12	1.10E-11	No
106	Lar2Hex	0.009	6.27E-01	6.36E-01	No
107	Pin2Hex	0.573	2.16E-19	1.17E-18	Yes
108	Coum-Sin3Hex I	0.656	1.15E-12	2.28E-12	Yes
109	Fer-Sin3Hex	0.183	8.20E-05	9.82E-05	No
110	Coum-Sin3Hex II	0.683	1.18E-19	8.23E-19	Yes
111	2Sin2Hex I	0.450	3.61E-16	1.13E-15	No
112	Sin-Fer2Hex I	0.452	1.06E-14	2.54E-14	No
113	Sin-hFer2Hex	0.683	1.40E-19	8.32E-19	Yes
114	Sin-Fer-dhFer2Hex	0.738	1.45E-16	4.67E-16	Yes
115	2Sin2Hex II	0.545	4.09E-12	7.27E-12	Yes
116	2Fer-dhFer2Hex	0.732	6.01E-12	1.05E-11	Yes
117	Sin-Fer2Hex II	0.542	1.12E-13	2.36E-13	Yes

118	2Sin2Hex III	0.379	1.64E-19	9.20E-19	No
119	Sin-Fer2Hex III	0.436	5.53E-21	5.74E-20	No
120	2Fer2Hex I	0.651	2.50E-14	5.72E-14	Yes
121	2Sin2Hex IV	0.332	2.25E-13	4.61E-13	No
122	Sin-Fer2Hex IV	0.273	2.10E-09	3.22E-09	No
123	2Fer2Hex II	0.237	8.15E-07	1.06E-06	No
124	Sin-SinAld2Hex	0.730	4.49E-15	1.14E-14	Yes
125	3Sin2Hex I	0.494	1.32E-14	3.06E-14	No
126	2Sin-Fer2Hex I	0.558	3.97E-16	1.22E-15	Yes
127	Sin-2Fer2Hex I	0.615	8.74E-16	2.51E-15	Yes
128	2SinHex	0.067	8.86E-03	9.97E-03	No
129	Sin-FerHex	0.219	2.93E-09	4.40E-09	No
130	3Sin2Hex II	0.561	1.76E-15	4.86E-15	Yes
131	2Sin-Fer2Hex II	0.357	1.07E-14	2.54E-14	No
132	2FerHex	1.163	2.27E-10	3.65E-10	Yes
133	Sin-2Fer2Hex II	0.297	5.54E-08	7.79E-08	No
134	3Sin2Hex III	0.342	1.10E-11	1.85E-11	No
135	2Sin-Fer2Hex III	0.100	9.56E-03	1.07E-02	No
		**Red-leaved kale**			
1	SyrQA	1.520	9.87E-14	1.90E-13	Yes
2	CafA2Hex	1.380	2.56E-17	9.11E-17	Yes
3	PrcAHex	1.288	1.24E-18	7.00E-18	Yes
4	CoumA2Hex I	2.664	1.08E-06	1.26E-06	Yes
5	CoumA2Hex II	1.998	7.20E-10	9.81E-10	Yes
6	GAHex	1.323	7.29E-19	5.29E-18	Yes
7	SinA2Hex I	1.180	3.20E-10	4.41E-10	Yes
8	CoumA2Hex III	1.884	4.69E-11	6.96E-11	Yes
9	FerA2Hex I	1.188	8.15E-10	1.10E-09	Yes
10	SinA2Hex II	1.256	8.55E-18	3.61E-17	Yes
11	CafAHex I	-0.089	8.20E-01	8.20E-01	No
12	hFerAHex I	-1.221	1.25E-17	4.98E-17	Yes
13	SinA2Pent	1.026	3.13E-16	8.45E-16	Yes
14	CafAHex II	0.914	1.52E-07	1.90E-07	Yes
15	hFerAHex II	1.126	7.27E-20	1.23E-18	Yes
16	FerA2Hex II	0.810	2.19E-17	8.00E-17	Yes
17	SinA2Hex III	1.028	4.76E-19	4.01E-18	Yes
18	FerAHex I	1.109	3.27E-16	8.65E-16	Yes
19	SinA2Hex IV	0.488	1.89E-10	2.68E-10	No
20	CoumAHex	1.020	2.86E-15	6.78E-15	Yes
21	SinAHex I	0.550	2.46E-12	4.01E-12	Yes
22	SinAHex II	1.511	2.26E-11	3.44E-11	Yes
23	FerAHex II	1.201	2.36E-16	6.50E-16	Yes
24	CafA	1.140	5.13E-17	1.47E-16	Yes
25	hFerA	1.468	3.79E-20	1.02E-18	Yes
26	SinA	0.954	3.34E-17	1.13E-16	Yes
27	CafQAHex I	1.714	4.20E-18	2.03E-17	Yes
28	CoumQAHex	1.725	5.71E-20	1.10E-18	Yes
29	CafQAHex II	1.258	9.63E-18	3.94E-17	Yes
30	CafQA I	1.148	1.94E-17	7.40E-17	Yes

31	FerQAHex	1.087	4.14E-09	5.42E-09	Yes
32	Sin-CafQAPentHex	0.589	2.76E-09	3.66E-09	Yes
33	CoumQA I	1.601	8.20E-20	1.23E-18	Yes
34	CafQA II	1.086	2.28E-06	2.64E-06	Yes
35	Sin-CafQAHex I	1.200	1.67E-07	2.07E-07	Yes
36	FerQA I	1.600	7.84E-19	5.29E-18	Yes
37	CafQA III	1.458	2.42E-14	4.79E-14	Yes
38	hFer-CafQAHex	1.822	4.62E-21	3.12E-19	Yes
39	CoumQA II	1.494	5.47E-20	1.10E-18	Yes
40	2Sin-CoumQA2Hex	1.363	4.52E-17	1.36E-16	Yes
41	Sin-2CoumQAHex	1.470	3.72E-17	1.20E-16	Yes
42	FerQA II	1.405	9.33E-19	6.00E-18	Yes
43	CoumQA III	0.862	2.23E-10	3.10E-10	Yes
44	2Sin-CafQA2Hex	2.867	9.99E-13	1.77E-12	Yes
45	FerQA III	1.110	3.17E-17	1.10E-16	Yes
46	SyringAld-SinQAHex	1.247	1.94E-12	3.27E-12	Yes
47	Fer-CafQAHex I	1.543	7.56E-21	3.40E-19	Yes
48	Sin-CafQAHex II	0.700	6.73E-15	1.49E-14	Yes
49	Sin-hFer-CafQAHex	1.656	1.50E-19	1.68E-18	Yes
50	Fer-CafQAHex II	0.624	2.08E-13	3.95E-13	Yes
51	2Sin-CafQAHex I	1.528	5.60E-19	4.45E-18	Yes
52	dhFer-2Sin-CoumQAHex	1.601	1.04E-19	1.40E-18	Yes
53	Fer-Sin-CafQAHex I	1.595	1.38E-19	1.68E-18	Yes
54	Fer-CafQA	0.941	1.18E-04	1.28E-04	Yes
55	2Sin-CafQAHex II	0.695	1.02E-12	1.79E-12	Yes
56	Fer-Sin-CafQAHex II	0.560	6.25E-08	7.96E-08	Yes
57	2Sin-CafQAHex III	0.370	2.24E-05	2.54E-05	No
58	Quer4Hex	0.784	2.02E-07	2.45E-07	Yes
59	Quer3Hex	0.982	4.77E-16	1.22E-15	Yes
60	QuerRham3Hex	0.682	1.09E-12	1.89E-12	Yes
61	Kamp3Hex I	1.164	7.38E-18	3.22E-17	Yes
62	Irham3Hex	0.807	8.30E-05	9.19E-05	Yes
63	Kamp-hFer5Hex	0.611	6.51E-12	1.03E-11	Yes
64	Quer-hFer4Hex	1.041	1.30E-07	1.64E-07	Yes
65	Quer-Caf4Hex	1.055	6.99E-13	1.29E-12	Yes
66	Kamp-Caf5Hex	0.580	9.54E-12	1.46E-11	Yes
67	Quer-Sin5Hex	0.407	1.18E-08	1.53E-08	No
68	Erio2Hex	0.442	5.25E-01	5.29E-01	No
69	Kamp4Hex	1.005	2.21E-12	3.68E-12	Yes
70	Kamp-hFer4Hex	0.805	1.98E-14	4.05E-14	Yes
71	Quer-hFer3Hex	2.194	1.96E-10	2.76E-10	Yes
72	Kamp-Sin5Hex	0.478	2.46E-12	4.01E-12	No
73	Kamp-Caf4Hex	1.099	5.94E-18	2.67E-17	Yes
74	Quer-Sin4Hex	0.291	3.62E-07	4.29E-07	No
75	Kamp-Fer5Hex	0.105	2.47E-03	2.56E-03	No
76	Kamp3Hex II	0.641	4.37E-12	7.03E-12	Yes
77	Kamp-hFer3Hex	0.346	1.41E-03	1.49E-03	No
78	Kamp-Sin4Hex	0.119	1.41E-02	1.43E-02	No
79	Quer-Sin3Hex	0.658	2.32E-15	5.59E-15	Yes

80	Kamp-Caf3Hex	0.888	6.94E-14	1.36E-13	Yes
81	Kamp-Fer4Hex	0.882	1.24E-14	2.64E-14	Yes
82	Kamp-Sin3Hex	1.233	4.61E-15	1.06E-14	Yes
83	Kamp-Fer3Hex	1.191	7.51E-19	5.29E-18	Yes
84	Kamp-2hFer5Hex	0.168	4.86E-04	5.21E-04	No
85	Kamp-hFer-Sin5Hex	0.082	6.51E-03	6.71E-03	No
86	Quer-2Sin5Hex	-0.096	8.05E-04	8.55E-04	No
87	Kamp-Sin3Hex-h	1.318	1.10E-18	6.78E-18	Yes
88	Kamp-Fer3Hex-h	0.948	3.77E-16	9.80E-16	Yes
89	Kamp-Fer-hFer5Hex	0.175	2.62E-06	3.00E-06	No
90	Kamp2Hex I	0.464	1.37E-12	2.34E-12	No
91	Kamp-2Sin5Hex	0.160	5.98E-05	6.67E-05	No
92	Quer2Hex	0.539	1.38E-10	1.98E-10	Yes
93	Kamp-Sin-hFer4Hex	0.809	7.52E-12	1.17E-11	Yes
94	Quer-2Sin4Hex	0.510	1.33E-10	1.93E-10	Yes
95	Kamp-Sin-Fer5Hex	0.123	1.07E-04	1.17E-04	No
96	Kamp-2Fer5Hex	0.250	1.22E-06	1.42E-06	No
97	Kamp-2Sin4Hex	1.168	2.50E-18	1.30E-17	Yes
98	Kamp2Hex II	0.650	5.96E-15	1.34E-14	Yes
99	Kamp-hFer2Hex	0.653	3.21E-13	6.03E-13	Yes
100	Kamp-Sin2Hex	0.316	1.92E-07	2.36E-07	No
101	Kamp-Sin-Fer3Hex	0.928	4.09E-17	1.28E-16	Yes
102	QuerGluc	0.695	1.54E-03	1.61E-03	Yes
103	Kamp-Fer2Hex	0.234	8.55E-05	9.38E-05	No
104	Cy-2Sin3Hex	1.046	4.71E-17	1.38E-16	Yes
105	Cy-Fer-Sin3Hex	0.902	3.55E-17	1.17E-16	Yes
106	Lar2Hex	0.119	1.41E-02	1.43E-02	No
107	Pin2Hex	0.800	7.37E-17	2.07E-16	Yes
108	Coum-Sin3Hex I	1.201	1.97E-17	7.40E-17	Yes
109	Fer-Sin3Hex	1.090	1.09E-15	2.67E-15	Yes
110	Coum-Sin3Hex II	0.732	2.40E-14	4.79E-14	Yes
111	2Sin2Hex I	0.944	4.35E-17	1.34E-16	Yes
112	Sin-Fer2Hex I	0.769	1.28E-14	2.65E-14	Yes
113	Sin-hFer2Hex	1.538	1.81E-21	2.45E-19	Yes
114	Sin-Fer-dhFer2Hex	1.441	4.40E-18	2.05E-17	Yes
115	2Sin2Hex II	0.519	1.37E-09	1.83E-09	Yes
116	2Fer-dhFer2Hex	1.505	2.86E-19	2.57E-18	Yes
117	Sin-Fer2Hex II	0.801	7.43E-15	1.62E-14	Yes
118	2Sin2Hex III	0.939	2.86E-19	2.57E-18	Yes
119	Sin-Fer2Hex III	1.027	1.19E-18	6.99E-18	Yes
120	2Fer2Hex I	1.377	2.32E-20	7.84E-19	Yes
121	2Sin2Hex IV	0.490	6.57E-12	1.03E-11	No
122	Sin-Fer2Hex IV	0.575	6.75E-11	9.91E-11	Yes
123	2Fer2Hex II	0.524	2.77E-08	3.56E-08	Yes
124	Sin-SinAld2Hex	1.534	4.44E-15	1.03E-14	Yes
125	3Sin2Hex I	1.123	3.19E-18	1.60E-17	Yes
126	2Sin-Fer2Hex I	1.258	2.18E-19	2.26E-18	Yes
127	Sin-2Fer2Hex I	1.366	1.50E-18	8.09E-18	Yes
128	2SinHex	0.887	1.25E-14	2.64E-14	Yes

129	Sin-FerHex	0.995	6.34E-16	1.58E-15	Yes
130	3Sin2Hex II	0.755	8.01E-13	1.44E-12	Yes
131	2Sin-Fer2Hex II	0.660	7.55E-13	1.38E-12	Yes
132	2FerHex	1.867	3.41E-07	4.11E-07	Yes
133	Sin-2Fer2Hex II	0.464	3.46E-07	4.13E-07	No
134	3Sin2Hex III	0.401	2.31E-11	3.46E-11	No
135	2Sin-Fer2Hex III	0.208	2.60E-05	2.92E-05	No

Table S5. Iodine-treatment-associated patterns in the steamed-versus-boiled phenolic response in green- and red-leaved kale. For each compound and cultivar, log_2FC was calculated as log_2[(mean LC–HRMS signal in steamed samples + 1)/(mean LC–HRMS signal in boiled samples + 1)] separately for Control, KIO_3, and DIQ treatments. Δlog_2FC was calculated relative to the corresponding iodine-free control: Δlog_2FC KIO_3 = log_2FC KIO_3 − log_2FC Control and Δlog_2FC DIQ = log_2FC DIQ − log_2FC Control. The final column shows Δlog_2FC DIQ − Δlog_2FC KIO_3. These values are used descriptively to characterize compound-level iodine-treatment-associated patterns and are not treated as independent biological replicates for statistical inference.

Compound No.	Compound	log2FC Control	log2FC KIO_3	log2FC DIQ	Δlog2FC KIO_3	Δlog2FC DIQ	DIQ − KIO_3
			**Green-leaved kale**				
1	SyrQA	0.435	1.204	1.096	0.769	0.661	-0.108
2	CafA2Hex	1.202	0.916	1.264	-0.287	0.061	0.348
3	PrcAHex	0.769	0.833	1.253	0.065	0.484	0.420
4	CoumA2Hex I	1.412	1.357	2.260	-0.055	0.849	0.903
5	CoumA2Hex II	0.997	1.405	0.995	0.408	-0.002	-0.410
6	GAHex	0.868	0.584	1.320	-0.284	0.451	0.736
7	SinA2Hex I	0.477	0.180	1.043	-0.297	0.566	0.863
8	CoumA2Hex III	0.456	0.713	1.795	0.257	1.340	1.082
9	FerA2Hex I	0.666	0.686	1.661	0.020	0.995	0.975
10	SinA2Hex II	0.340	0.665	1.234	0.325	0.895	0.569
11	CafAHex I	1.104	0.671	1.031	-0.433	-0.074	0.360
12	hFerAHex I	-0.188	0.233	-0.566	0.421	-0.377	-0.798
13	SinA2Pent	0.392	0.697	1.106	0.305	0.713	0.409
14	CafAHex II	-0.329	0.868	-0.537	1.198	-0.208	-1.405
15	hFerAHex II	0.483	0.258	0.685	-0.225	0.202	0.428
16	FerA2Hex II	0.153	0.457	0.742	0.304	0.589	0.286
17	SinA2Hex III	0.353	0.369	0.886	0.015	0.533	0.518
18	FerAHex I	1.025	1.473	0.652	0.448	-0.373	-0.821
19	SinA2Hex IV	0.068	0.713	0.302	0.644	0.234	-0.411
20	CoumAHex	0.963	1.050	0.698	0.086	-0.265	-0.352
21	SinAHex I	0.014	-0.162	-0.003	-0.175	-0.017	0.159
22	SinAHex II	0.502	0.325	1.146	-0.176	0.644	0.821
23	FerAHex II	0.887	0.524	1.215	-0.363	0.327	0.691
24	CafA	0.747	1.817	0.910	1.070	0.163	-0.907
25	hFerA	0.846	1.941	0.666	1.096	-0.179	-1.275
26	SinA	0.268	1.698	0.785	1.430	0.517	-0.913
27	CafQAHex I	0.756	0.887	1.361	0.131	0.606	0.475
28	CoumQAHex	0.692	0.897	1.587	0.205	0.895	0.691
29	CafQAHex II	0.456	0.607	1.076	0.151	0.619	0.469
30	CafQA I	0.542	0.511	1.036	-0.031	0.494	0.525
31	FerQAHex	0.483	0.631	1.421	0.148	0.938	0.790
32	Sin-CafQAPentHex	0.354	0.280	0.602	-0.075	0.248	0.322
33	CoumQA I	0.659	0.572	1.353	-0.087	0.694	0.781

34	CafQA II	0.472	0.305	0.886	-0.167	0.413	0.581
35	Sin-CafQAHex I	0.251	0.771	0.788	0.520	0.538	0.017
36	FerQA I	0.629	0.668	1.258	0.039	0.629	0.589
37	CafQA III	0.776	0.923	1.500	0.147	0.724	0.576
38	hFer-CafQAHex	1.020	0.611	1.806	-0.409	0.786	1.195
39	CoumQA II	0.590	1.157	1.317	0.567	0.727	0.160
40	2Sin-CoumQA2Hex	0.533	0.545	1.282	0.012	0.749	0.737
41	Sin-2CoumQAHex	0.673	0.722	1.487	0.049	0.815	0.765
42	FerQA II	0.505	1.018	1.144	0.513	0.639	0.126
43	CoumQA III	0.748	1.094	1.380	0.346	0.631	0.285
44	2Sin-CafQA2Hex	1.598	1.432	2.929	-0.166	1.331	1.497
45	FerQA III	0.647	0.764	1.269	0.117	0.622	0.506
46	SyringAld-SinQAHex	1.191	1.547	2.406	0.356	1.216	0.860
47	Fer-CafQAHex I	0.589	0.298	1.241	-0.291	0.651	0.942
48	Sin-CafQAHex II	0.167	0.784	0.635	0.617	0.467	-0.149
49	Sin-hFer-CafQAHex	0.887	0.249	1.641	-0.638	0.754	1.392
50	Fer-CafQAHex II	-0.182	0.656	0.567	0.838	0.749	-0.089
51	2Sin-CafQAHex I	0.821	0.406	1.568	-0.415	0.747	1.162
52	dhFer-2Sin-CoumQAHex	0.675	0.523	1.249	-0.152	0.574	0.726
53	Fer-Sin-CafQAHex I	0.694	0.336	1.613	-0.357	0.920	1.277
54	Fer-CafQA	-0.074	-0.575	0.632	-0.501	0.706	1.207
55	2Sin-CafQAHex II	0.135	0.343	0.782	0.208	0.648	0.440
56	Fer-Sin-CafQAHex II	-0.151	0.424	0.555	0.575	0.706	0.131
57	2Sin-CafQAHex III	-0.141	0.803	0.173	0.943	0.314	-0.629
58	Quer4Hex	0.375	0.652	2.976	0.277	2.601	2.324
59	Quer3Hex	0.424	0.256	0.791	-0.168	0.367	0.535
60	QuerRham3Hex	0.445	0.612	0.690	0.168	0.245	0.077
61	Kamp3Hex I	0.726	0.514	1.213	-0.212	0.487	0.699
62	Irham3Hex	0.681	0.351	0.873	-0.330	0.193	0.522
63	Kamp-hFer5Hex	0.444	0.091	0.748	-0.354	0.303	0.657
64	Quer-hFer4Hex	0.734	0.717	1.394	-0.017	0.659	0.676
65	Quer-Caf4Hex	0.052	0.512	1.020	0.460	0.969	0.508
66	Kamp-Caf5Hex	0.497	0.055	1.318	-0.442	0.821	1.262
67	Quer-Sin5Hex	0.304	0.095	0.379	-0.209	0.075	0.284
68	Erio2Hex	0.292	0.102	0.448	-0.190	0.156	0.346
69	Kamp4Hex	0.085	-0.074	0.381	-0.159	0.297	0.455
70	Kamp-hFer4Hex	0.128	0.083	0.455	-0.045	0.327	0.372
71	Quer-hFer3Hex	0.518	0.096	1.098	-0.422	0.580	1.002
72	Kamp-Sin5Hex	-0.106	-0.133	0.055	-0.027	0.160	0.187
73	Kamp-Caf4Hex	0.064	0.394	0.690	0.330	0.626	0.296
74	Quer-Sin4Hex	0.045	-0.080	0.131	-0.124	0.086	0.211
75	Kamp-Fer5Hex	-0.099	-0.351	0.222	-0.252	0.321	0.573
76	Kamp3Hex II	0.220	0.358	0.611	0.138	0.391	0.253
77	Kamp-hFer3Hex	-0.032	-0.542	0.351	-0.510	0.383	0.893
78	Kamp-Sin4Hex	-0.219	0.030	0.218	0.250	0.437	0.188
79	Quer-Sin3Hex	0.105	-0.112	0.587	-0.217	0.483	0.699
80	Kamp-Caf3Hex	-0.056	-0.173	0.549	-0.117	0.605	0.722
81	Kamp-Fer4Hex	0.044	0.234	0.734	0.189	0.690	0.500
82	Kamp-Sin3Hex	0.129	0.338	0.906	0.209	0.777	0.569

83	Kamp-Fer3Hex	0.184	0.040	0.804	-0.144	0.620	0.765
84	Kamp-2hFer5Hex	0.192	-0.838	0.155	-1.030	-0.037	0.993
85	Kamp-hFer-Sin5Hex	-0.461	-0.775	-0.303	-0.315	0.157	0.472
86	Quer-2Sin5Hex	-0.582	-0.851	-0.756	-0.270	-0.174	0.095
87	Kamp-Sin3Hex-h	0.554	0.521	1.023	-0.032	0.469	0.501
88	Kamp-Fer3Hex-h	0.486	0.493	1.123	0.007	0.637	0.630
89	Kamp-Fer-hFer5Hex	-0.021	-0.554	0.028	-0.532	0.050	0.582
90	Kamp2Hex I	0.086	-0.024	0.665	-0.111	0.579	0.690
91	Kamp-2Sin5Hex	-0.411	-0.464	-0.354	-0.053	0.058	0.110
92	Quer2Hex	0.273	-0.156	0.723	-0.429	0.450	0.879
93	Kamp-Sin-hFer4Hex	0.009	-0.077	0.395	-0.087	0.385	0.472
94	Quer-2Sin4Hex	-0.201	-0.151	0.112	0.051	0.313	0.263
95	Kamp-Sin-Fer5Hex	-0.172	-0.454	-0.096	-0.282	0.075	0.358
96	Kamp-2Fer5Hex	-0.310	-0.849	0.820	-0.539	1.130	1.669
97	Kamp-2Sin4Hex	0.070	0.258	0.453	0.188	0.383	0.195
98	Kamp2Hex II	0.095	-0.059	1.014	-0.153	0.919	1.073
99	Kamp-hFer2Hex	-0.351	-0.243	0.995	0.108	1.346	1.238
100	Kamp-Sin2Hex	-0.787	-0.416	0.561	0.371	1.348	0.977
101	Kamp-Sin-Fer3Hex	0.555	0.583	0.956	0.027	0.400	0.373
102	QuerGluc	0.491	0.780	1.012	0.289	0.521	0.232
103	Kamp-Fer2Hex	-0.673	-0.382	0.954	0.291	1.628	1.336
104	Cy-2Sin3Hex	0.151	0.666	0.518	0.516	0.367	-0.149
105	Cy-Fer-Sin3Hex	0.258	0.397	0.518	0.139	0.260	0.121
106	Lar2Hex	-0.219	0.030	0.218	0.250	0.437	0.188
107	Pin2Hex	0.570	0.473	0.677	-0.096	0.107	0.203
108	Coum-Sin3Hex I	0.526	0.338	1.095	-0.188	0.569	0.757
109	Fer-Sin3Hex	0.169	-0.277	0.657	-0.446	0.488	0.934
110	Coum-Sin3Hex II	0.347	0.800	0.903	0.453	0.556	0.103
111	2Sin2Hex I	0.237	0.389	0.724	0.152	0.487	0.335
112	Sin-Fer2Hex I	0.165	0.475	0.715	0.311	0.551	0.240
113	Sin-hFer2Hex	0.638	0.191	1.221	-0.447	0.583	1.029
114	Sin-Fer-dhFer2Hex	0.617	0.393	1.206	-0.224	0.589	0.814
115	2Sin2Hex II	0.249	1.075	0.313	0.826	0.064	-0.762
116	2Fer-dhFer2Hex	0.470	0.414	1.301	-0.056	0.831	0.887
117	Sin-Fer2Hex II	0.067	1.060	0.499	0.993	0.432	-0.561
118	2Sin2Hex III	0.257	0.206	0.674	-0.051	0.417	0.468
119	Sin-Fer2Hex III	0.243	0.246	0.819	0.003	0.576	0.573
120	2Fer2Hex I	0.393	0.470	1.093	0.078	0.701	0.623
121	2Sin2Hex IV	0.017	0.710	0.268	0.694	0.251	-0.443
122	Sin-Fer2Hex IV	-0.204	0.648	0.374	0.852	0.578	-0.274
123	2Fer2Hex II	-0.315	0.815	0.216	1.130	0.531	-0.599
124	Sin-SinAld2Hex	0.443	0.465	1.286	0.022	0.843	0.821
125	3Sin2Hex I	0.328	0.338	0.818	0.009	0.490	0.480
126	2Sin-Fer2Hex I	0.234	0.393	1.048	0.159	0.814	0.654
127	Sin-2Fer2Hex I	0.176	0.485	1.189	0.309	1.013	0.704
128	2SinHex	-0.113	-0.549	0.866	-0.436	0.979	1.415
129	Sin-FerHex	0.070	-0.405	0.992	-0.475	0.922	1.397
130	3Sin2Hex II	0.229	0.898	0.558	0.669	0.328	-0.340
131	2Sin-Fer2Hex II	-0.061	0.662	0.471	0.723	0.532	-0.191

132	2FerHex	0.984	1.038	1.430	0.055	0.447	0.392
133	Sin-2Fer2Hex II	-0.329	0.831	0.392	1.160	0.721	-0.439
134	3Sin2Hex III	-0.097	1.145	-0.020	1.243	0.077	-1.166
135	2Sin-Fer2Hex III	-0.424	0.918	-0.194	1.342	0.230	-1.112
			**Red-leaved kale**				
1	SyrQA	1.257	2.012	1.286	0.755	0.029	-0.726
2	CafA2Hex	1.755	1.458	0.927	-0.297	-0.829	-0.532
3	PrcAHex	1.432	1.436	0.993	0.004	-0.439	-0.443
4	CoumA2Hex I	3.150	1.908	2.880	-1.241	-0.270	0.971
5	CoumA2Hex II	2.246	2.195	1.355	-0.051	-0.891	-0.840
6	GAHex	1.496	1.450	1.020	-0.046	-0.476	-0.430
7	SinA2Hex I	1.739	0.902	0.819	-0.837	-0.920	-0.083
8	CoumA2Hex III	2.332	2.015	1.311	-0.317	-1.021	-0.704
9	FerA2Hex I	1.352	1.186	0.995	-0.166	-0.357	-0.191
10	SinA2Hex II	1.243	1.464	1.055	0.221	-0.188	-0.409
11	CafAHex I	-0.273	0.331	-0.319	0.604	-0.046	-0.650
12	hFerAHex I	-1.551	-0.569	-1.539	0.982	0.012	-0.970
13	SinA2Pent	1.214	1.113	0.751	-0.100	-0.462	-0.362
14	CafAHex II	1.082	1.479	0.274	0.397	-0.808	-1.204
15	hFerAHex II	1.074	1.845	0.459	0.771	-0.615	-1.386
16	FerA2Hex II	0.832	0.920	0.676	0.088	-0.156	-0.244
17	SinA2Hex III	1.204	1.180	0.699	-0.024	-0.504	-0.481
18	FerAHex I	1.052	1.403	0.873	0.350	-0.180	-0.530
19	SinA2Hex IV	0.406	0.732	0.323	0.326	-0.083	-0.409
20	CoumAHex	0.966	1.385	0.703	0.419	-0.263	-0.682
21	SinAHex I	0.625	0.474	0.548	-0.151	-0.077	0.074
22	SinAHex II	1.720	1.641	1.094	-0.079	-0.625	-0.546
23	FerAHex II	1.365	1.496	0.754	0.130	-0.612	-0.742
24	CafA	0.698	1.897	0.822	1.199	0.125	-1.074
25	hFerA	0.330	3.231	0.840	2.901	0.510	-2.391
26	SinA	0.433	1.646	0.782	1.212	0.349	-0.864
27	CafQAHex I	1.930	1.851	1.356	-0.079	-0.574	-0.495
28	CoumQAHex	2.013	1.787	1.376	-0.226	-0.637	-0.411
29	CafQAHex II	1.413	1.341	1.014	-0.072	-0.400	-0.328
30	CafQA I	1.086	1.491	0.860	0.405	-0.226	-0.630
31	FerQAHex	1.027	1.091	1.062	0.064	0.035	-0.029
32	Sin-CafQAPentHex	0.892	0.540	0.329	-0.352	-0.563	-0.211
33	CoumQA I	1.766	1.709	1.325	-0.057	-0.440	-0.383
34	CafQA II	1.138	1.250	0.909	0.111	-0.229	-0.341
35	Sin-CafQAHex I	1.388	1.058	1.060	-0.331	-0.328	0.002
36	FerQA I	1.779	1.717	1.302	-0.061	-0.477	-0.415
37	CafQA III	1.251	1.818	1.281	0.567	0.030	-0.537
38	hFer-CafQAHex	1.727	2.439	1.297	0.712	-0.430	-1.142
39	CoumQA II	1.600	1.579	1.300	-0.021	-0.300	-0.279
40	2Sin-CoumQA2Hex	1.781	1.253	1.050	-0.528	-0.731	-0.204
41	Sin-2CoumQAHex	1.763	1.367	1.264	-0.396	-0.499	-0.103
42	FerQA II	1.463	1.542	1.202	0.080	-0.261	-0.340
43	CoumQA III	0.644	1.181	0.765	0.537	0.121	-0.417
44	2Sin-CafQA2Hex	2.669	3.702	2.148	1.032	-0.522	-1.554

45	FerQA III	0.989	1.327	1.010	0.338	0.021	-0.317
46	SyringAld-SinQAHex	1.066	1.287	1.390	0.221	0.324	0.103
47	Fer-CafQAHex I	1.488	2.043	1.095	0.554	-0.393	-0.948
48	Sin-CafQAHex II	0.484	1.373	0.238	0.890	-0.246	-1.136
49	Sin-hFer-CafQAHex	1.745	2.107	1.109	0.362	-0.636	-0.998
50	Fer-CafQAHex II	0.312	1.194	0.366	0.882	0.054	-0.828
51	2Sin-CafQAHex I	1.608	1.903	1.073	0.295	-0.535	-0.829
52	dhFer-2Sin-CoumQAHex	1.810	1.815	1.176	0.004	-0.635	-0.639
53	Fer-Sin-CafQAHex I	1.647	1.881	1.254	0.234	-0.393	-0.627
54	Fer-CafQA	1.163	1.743	0.183	0.579	-0.980	-1.559
55	2Sin-CafQAHex II	0.586	1.108	0.387	0.522	-0.199	-0.721
56	Fer-Sin-CafQAHex II	0.367	0.996	0.314	0.629	-0.052	-0.682
57	2Sin-CafQAHex III	0.121	0.924	0.062	0.803	-0.059	-0.862
58	Quer4Hex	0.817	0.795	0.736	-0.022	-0.081	-0.060
59	Quer3Hex	0.914	1.515	0.511	0.601	-0.403	-1.004
60	QuerRham3Hex	0.743	1.043	0.251	0.300	-0.493	-0.792
61	Kamp3Hex I	1.175	1.524	0.792	0.349	-0.383	-0.732
62	Irham3Hex	1.318	0.790	0.486	-0.528	-0.832	-0.304
63	Kamp-hFer5Hex	0.821	0.962	0.042	0.140	-0.779	-0.920
64	Quer-hFer4Hex	0.827	2.034	0.352	1.207	-0.474	-1.682
65	Quer-Caf4Hex	1.038	1.577	0.530	0.539	-0.508	-1.047
66	Kamp-Caf5Hex	0.872	0.668	0.191	-0.204	-0.681	-0.477
67	Quer-Sin5Hex	0.517	0.714	-0.020	0.197	-0.537	-0.734
68	Erio2Hex	-0.047	2.004	-0.012	2.051	0.035	-2.016
69	Kamp4Hex	1.031	1.455	0.501	0.424	-0.530	-0.954
70	Kamp-hFer4Hex	0.719	1.342	0.346	0.623	-0.372	-0.995
71	Quer-hFer3Hex	2.894	1.506	2.452	-1.388	-0.442	0.946
72	Kamp-Sin5Hex	0.637	0.808	-0.016	0.171	-0.653	-0.824
73	Kamp-Caf4Hex	1.009	1.598	0.687	0.589	-0.322	-0.911
74	Quer-Sin4Hex	0.372	0.685	-0.185	0.313	-0.557	-0.870
75	Kamp-Fer5Hex	0.261	0.345	-0.295	0.085	-0.556	-0.640
76	Kamp3Hex II	0.635	0.894	0.393	0.260	-0.242	-0.502
77	Kamp-hFer3Hex	0.267	0.617	0.160	0.351	-0.106	-0.457
78	Kamp-Sin4Hex	0.228	0.599	-0.471	0.371	-0.699	-1.070
79	Quer-Sin3Hex	0.814	0.991	0.169	0.177	-0.645	-0.822
80	Kamp-Caf3Hex	0.994	1.058	0.618	0.064	-0.376	-0.440
81	Kamp-Fer4Hex	0.868	1.359	0.418	0.490	-0.451	-0.941
82	Kamp-Sin3Hex	1.196	1.845	0.658	0.649	-0.539	-1.187
83	Kamp-Fer3Hex	1.195	1.555	0.823	0.360	-0.372	-0.732
84	Kamp-2hFer5Hex	0.155	0.535	-0.192	0.381	-0.346	-0.727
85	Kamp-hFer-Sin5Hex	0.161	0.281	-0.201	0.120	-0.362	-0.482
86	Quer-2Sin5Hex	0.025	-0.086	-0.227	-0.111	-0.253	-0.141
87	Kamp-Sin3Hex-h	0.984	2.382	0.585	1.398	-0.399	-1.797
88	Kamp-Fer3Hex-h	0.773	1.781	0.288	1.007	-0.485	-1.492
89	Kamp-Fer-hFer5Hex	0.226	0.319	-0.022	0.093	-0.248	-0.340
90	Kamp2Hex I	0.644	0.604	0.147	-0.040	-0.497	-0.457
91	Kamp-2Sin5Hex	0.208	0.464	-0.191	0.255	-0.400	-0.655
92	Quer2Hex	0.633	0.748	0.235	0.115	-0.399	-0.514
93	Kamp-Sin-hFer4Hex	0.517	1.431	0.478	0.913	-0.040	-0.953

94	Quer-2Sin4Hex	0.309	0.983	0.230	0.674	-0.079	-0.752
95	Kamp-Sin-Fer5Hex	0.224	0.266	-0.121	0.042	-0.346	-0.388
96	Kamp-2Fer5Hex	0.418	0.149	0.180	-0.269	-0.238	0.031
97	Kamp-2Sin4Hex	0.879	1.799	0.821	0.919	-0.058	-0.977
98	Kamp2Hex II	0.703	0.860	0.387	0.157	-0.316	-0.473
99	Kamp-hFer2Hex	0.674	0.937	0.349	0.264	-0.325	-0.589
100	Kamp-Sin2Hex	0.598	0.186	0.157	-0.412	-0.441	-0.029
101	Kamp-Sin-Fer3Hex	1.101	1.310	0.370	0.209	-0.731	-0.940
102	QuerGluc	0.601	0.462	1.287	-0.140	0.685	0.825
103	Kamp-Fer2Hex	0.323	0.263	0.106	-0.060	-0.217	-0.157
104	Cy-2Sin3Hex	1.151	1.536	0.449	0.385	-0.702	-1.087
105	Cy-Fer-Sin3Hex	0.995	1.328	0.380	0.333	-0.615	-0.948
106	Lar2Hex	0.228	0.599	-0.471	0.371	-0.699	-1.070
107	Pin2Hex	0.980	0.916	0.503	-0.064	-0.477	-0.413
108	Coum-Sin3Hex I	1.578	1.049	0.972	-0.529	-0.606	-0.077
109	Fer-Sin3Hex	1.680	0.960	0.636	-0.720	-1.044	-0.325
110	Coum-Sin3Hex II	0.977	0.625	0.593	-0.351	-0.384	-0.033
111	2Sin2Hex I	1.078	0.966	0.786	-0.112	-0.292	-0.180
112	Sin-Fer2Hex I	0.640	0.885	0.775	0.245	0.135	-0.109
113	Sin-hFer2Hex	1.516	2.089	1.006	0.573	-0.510	-1.083
114	Sin-Fer-dhFer2Hex	1.515	1.826	0.977	0.311	-0.537	-0.848
115	2Sin2Hex II	0.270	0.737	0.541	0.467	0.270	-0.196
116	2Fer-dhFer2Hex	1.434	1.752	1.320	0.319	-0.114	-0.432
117	Sin-Fer2Hex II	0.356	1.121	0.920	0.765	0.564	-0.200
118	2Sin2Hex III	0.987	1.236	0.592	0.249	-0.394	-0.644
119	Sin-Fer2Hex III	0.984	1.307	0.786	0.322	-0.198	-0.521
120	2Fer2Hex I	1.204	1.632	1.293	0.429	0.089	-0.340
121	2Sin2Hex IV	0.414	0.947	0.108	0.533	-0.306	-0.839
122	Sin-Fer2Hex IV	0.379	0.988	0.359	0.608	-0.021	-0.629
123	2Fer2Hex II	0.311	0.803	0.457	0.492	0.146	-0.346
124	Sin-SinAld2Hex	1.553	1.717	1.317	0.163	-0.236	-0.400
125	3Sin2Hex I	1.161	1.453	0.752	0.293	-0.409	-0.701
126	2Sin-Fer2Hex I	1.274	1.477	1.018	0.203	-0.256	-0.459
127	Sin-2Fer2Hex I	1.342	1.467	1.284	0.125	-0.058	-0.184
128	2SinHex	1.186	0.945	0.521	-0.240	-0.664	-0.424
129	Sin-FerHex	1.047	1.117	0.818	0.070	-0.229	-0.299
130	3Sin2Hex II	0.715	1.063	0.481	0.347	-0.234	-0.582
131	2Sin-Fer2Hex II	0.552	0.887	0.535	0.335	-0.017	-0.352
132	2FerHex	1.985	1.598	1.629	-0.386	-0.356	0.030
133	Sin-2Fer2Hex II	0.268	0.551	0.567	0.283	0.299	0.016
134	3Sin2Hex III	0.321	0.690	0.188	0.369	-0.133	-0.502
135	2Sin-Fer2Hex III	-0.050	0.478	0.194	0.528	0.245	-0.283

Table S6. Calibration parameters and analytical performance characteristics for the reference standards used for quantitative and semi-quantitative determination of selected phenolic compounds. Calibration curves were constructed using seven concentration levels with three replicate measurements at each level (n = 21). LOD and LOQ were calculated as 3.3σ/S and 10σ/S, respectively, where σ represents the standard error of the regression intercept and S represents the slope of the calibration curve. Calibration ranges are expressed in mg/mL. LOD and LOQ are reported both for the analyzed extracts (mg/mL) and on a dry-weight basis (mg/g DW), calculated using an extraction volume of 2.5 mL and a sample mass of 0.200 g.

Reference standard	Calibration range	Calibration equation	R²	Sy.x	SE intercept (σ)	LOD mg/mL	LOQ mg/mL	LOD mg/g dw	LOQ mg/g dw
Neochlorogenic acid	0.0100-0.2000	y = 19,924,869.18x + 44,640.38	0.99505	99,182.65	36,281.65	0.00601	0.01821	0.0751	0.2276
Chlorogenic acid	0.0099-0.1980	y = 23,617,305.13x + 39,126.68	0.99649	98,285.62	35,779.84	0.00500	0.01515	0.0625	0.1894
Sinapic acid	0.00565-0.1130	y = 38,058,848.63x + 67,517.07	0.99612	95,134.32	34,632.64	0.00300	0.00910	0.0375	0.1138
Kaempferol	0.00623-0.1246	y = 16,193,572.05x − 24,992.63	0.99693	39,639.59	14,430.37	0.00294	0.00891	0.0368	0.1114

Table S7. Contents of selected major phenolic compounds in green- and red-leaved kale subjected to different thermal processing and iodine treatments. Values are expressed as mean ± SD (independently processed portions derived from the same pooled plant material), in mg/g DW. CafQA I and CafQA II were quantified using authentic neochlorogenic acid and chlorogenic acid standards, respectively. Sinapic acid derivatives were semi-quantified as sinapic acid equivalents, whereas Kamp-hFer4Hex was expressed as kaempferol equivalents. Compounds 118 and 119 co-eluted and were therefore determined together as a single PDA peak. † At least one of the three independently processed portions was below the corresponding LOQ. Back-calculated values were retained for calculation of group means and statistical analyses. For compound 17, two replicates in boiled red-leaved Control samples and all three replicates in boiled red-leaved KIO_3 samples were below the LOD.

Treatment		17 SinA2Hex III	22 SinAHex II	30 CafQA I	34 CafQA II	70 Kamp-hFer4Hex	113 Sin-hFer2Hex	118+119 2Sin2Hex III + Sin-Fer2Hex III	126 2Sin-Fer2Hex I
					**Green-leaved kale**				
Raw	Ctrl	0.361 ±0.028	0.626 ±0.015	2.076 ±0.069	1.957 ±0.077	0.981 ±0.036	1.234 ±0.022	1.938 ±0.034	1.085 ±0.033
Blanched	Ctrl	0.171 ±0.012	0.349 ±0.013	1.089 ±0.039	1.201 ±0.022	0.7097 ±0.0091	0.582 ±0.037	0.7869 ±0.0048	0.425 ±0.015
	KIO_3	0.1163 ±0.0013	0.3327 ±0.0022	0.854 ±0.046	1.159 ±0.023	0.800 ±0.056	0.668 ±0.024	0.713 ±0.017	0.3407 ±0.0053
	DIQ	0.1536 ±0.0024	0.4131 ±0.0080	1.726 ±0.030	1.412 ±0.034	0.683 ±0.016	0.739 ±0.014	1.129 ±0.026	0.657 ±0.012
Boiled	Ctrl	0.1581 ±0.0075	0.337 ±0.031	1.172 ±0.053	1.320 ±0.054	0.761 ±0.019	0.494 ±0.025	0.801 ±0.023	0.475 ±0.011
	KIO_3	0.1887 ±0.0040	0.40427 ±0.00032	1.475 ±0.035	1.492 ±0.037	0.979 ±0.030	0.8151 ±0.0089	1.1671 ±0.0054	0.577 ±0.011
	DIQ	0.0996 ±0.0032 †	0.168 ±0.070	0.795 ±0.041	1.0808 ±0.0087	0.735 ±0.011	0.403 ±0.020	0.498 ±0.021	0.234 ±0.010
Steamed	Ctrl	0.2295 ±0.0067	0.455 ±0.047	1.769 ±0.031	1.738 ±0.052	0.869 ±0.029	0.841 ±0.035	1.083 ±0.016	0.597 ±0.016
	KIO_3	0.272 ±0.014	0.508 ±0.048	2.202 ±0.019	1.813 ±0.013	1.041 ±0.049	0.973 ±0.019	1.523 ±0.033	0.848 ±0.046
	DIQ	0.2666 ±0.0074	0.481 ±0.040	1.736 ±0.018	1.727 ±0.044	0.957 ±0.012	1.103 ±0.058	1.370 ±0.032	0.780 ±0.038
					**Red-leaved kale**				
Raw	Ctrl	0.2083 ±0.0054	0.6225 ±0.0084	1.0199 ±0.0057	0.456 ±0.054	0.510 ±0.018	0.755 ±0.018	1.945 ±0.020	1.152 ±0.022
Blanched	Ctrl	0.0888 ±0.011 †	0.253 ±0.0076	0.707 ±0.026	0.385 ±0.031	0.416 ±0.033	0.3827 ±0.0096	0.812 ±0.011	0.401 ±0.032
	KIO_3	0.157 ±0.011	0.468 ±0.041	1.213 ±0.090	0.446 ±0.065	0.4572 ±0.0073	0.559 ±0.046	1.25 ±0.13	0.589 ±0.036
	DIQ	0.123 ±0.015 †	0.371 ±0.032	1.048 ±0.085	0.690 ±0.068	0.539 ±0.014	0.589 ±0.014	1.004 ±0.082	0.555 ±0.053
Boiled	Ctrl	0.0371 ±0.011 †	0.144 ±0.073 †	0.448 ±0.076	0.474 ±0.078	0.335 ±0.028	0.152 ±0.025	0.378 ±0.069	0.226 ±0.049
	KIO_3	0.0346 ±0.0014 †	0.145 ±0.034 †	0.3284 ±0.0044	0.417 ±0.025	0.2599 ±0.0019	0.090 ±0.014 †	0.254 ±0.015	0.1448 ±0.0062
	DIQ	0.0783 ±0.0042 †	0.2264 ±0.0067	0.559 ±0.017	0.478 ±0.014	0.331 ±0.022	0.203 ±0.016	0.430 ±0.026	0.2606 ±0.0027
Steamed	Ctrl	0.2142 ±0.0041	0.547 ±0.071	1.110 ±0.023	0.706 ±0.014	0.527 ±0.011	0.635 ±0.016	1.399 ±0.074	0.937 ±0.053
	KIO_3	0.2093 ±0.0014	0.552 ±0.013	1.2171 ±0.0029	0.591 ±0.12	0.5575 ±0.0097	0.760 ±0.017	1.643 ±0.047	0.906 ±0.033
	DIQ	0.2035 ±0.0024	0.534 ±0.027	1.1217 ±0.0057	0.617 ±0.037	0.420 ±0.026	0.524 ±0.018	1.187 ±0.023	0.776 ±0.023

The sensitivity of the statistical conclusions to the treatment of measurements below the LOQ is presented in Table S8.

Table S8. Sensitivity analysis of processing-related statistical comparisons to the treatment of measurements below the limit of quantification (LOQ). Only cultivar × compound × iodine-treatment combinations containing at least one measurement below the corresponding LOQ are included. The primary analysis retained back-calculated concentrations. Sensitivity analyses were performed by replacing measurements below the LOQ with either LOQ/2 or LOQ, followed by one-way ANOVA comparing blanched, boiled, and steamed samples within each cultivar × compound × iodine-treatment combination. Significant pairwise differences identified by Tukey-adjusted post-hoc comparisons ($p < 0.05$) are indicated as B–Bo (Blanched vs Boiled), B–S (Blanched vs Steamed), and Bo–S (Boiled vs Steamed).

Cultivar	Compound	Iodine	Treatment of <LOQ values	One-way ANOVA p	Tukey post-hoc differences
Green	17	DIQ	Back-calculated	<0.0001	B–Bo, B–S, Bo–S
			LOQ/2	<0.0001	B–Bo, B–S, Bo–S
			LOQ	<0.0001	B–Bo, B–S, Bo–S
Red	17	Control	Back-calculated	<0.0001	B–Bo, B–S, Bo–S
			LOQ/2	<0.0001	B–S, Bo–S
			LOQ	<0.0001	B–S, Bo–S
Red	17	KIO_3	Back-calculated	<0.0001	B–Bo, B–S, Bo–S
			LOQ/2	<0.0001	B–Bo, B–S, Bo–S
			LOQ	<0.0001	B–Bo, B–S, Bo–S
Red	17	DIQ	Back-calculated	<0.0001	B–Bo, B–S, Bo–S
			LOQ/2	0.0010	B–S, Bo–S
			LOQ	<0.0001	B–S, Bo–S
Red	22	Control	Back-calculated	0.0004	B–S, Bo–S
			LOQ/2	0.0008	B–S, Bo–S
			LOQ	0.0003	B–S, Bo–S
Red	22	KIO_3	Back-calculated	<0.0001	B–Bo, B–S, Bo–S
			LOQ/2	<0.0001	B–Bo, Bo–S
			LOQ	<0.0001	B–Bo, B–S, Bo–S
Red	113	KIO_3	Back-calculated	<0.0001	B–Bo, B–S, Bo–S
			LOQ/2	<0.0001	B–Bo, B–S, Bo–S
			LOQ	<0.0001	B–Bo, B–S, Bo–S
